# Identification of rat lung – prominent genes by a parallel DNA microarray hybridization

**DOI:** 10.1186/1471-2164-7-47

**Published:** 2006-03-13

**Authors:** Zhongming Chen, Jiwang Chen, Tingting Weng, Nili Jin, Lin Liu

**Affiliations:** 1 Department of Physiological Sciences, Oklahoma State University, Stillwater, Oklahoma 74078, USA

## Abstract

**Background:**

The comparison of organ transcriptomes is an important strategy for understanding gene functions. In the present study, we attempted to identify lung-prominent genes by comparing the normal transcriptomes of rat lung, heart, kidney, liver, spleen, and brain. To increase the efficiency and reproducibility, we first developed a novel parallel hybridization system, in which 6 samples could be hybridized onto a single slide at the same time.

**Results:**

We identified the genes prominently expressed in the lung (147) or co-expressed in lung-heart (23), lung-liver (37), lung-spleen (203), and lung-kidney (98). The known functions of the lung-prominent genes mainly fell into 5 categories: ligand binding, signal transducer, cell communication, development, and metabolism. Real-time PCR confirmed 13 lung-prominent genes, including 5 genes that have not been investigated in the lung, vitamin D-dependent calcium binding protein (Calb3), mitogen activated protein kinase 13 (Mapk13), solute carrier family 29 transporters, member 1 (Slc29a1), corticotropin releasing hormone receptor (Crhr1), and lipocalin 2 (Lcn2).

**Conclusion:**

The lung-prominent genes identified in this study may provide an important clue for further investigation of pulmonary functions.

## Background

With the completion of genome projects of human and other model species, functional studies on a genomic scale are coming to a frontier. The investigation of transcriptome reveals gene expression of organs and cells from normal and diseased animals and humans. By comparing transcriptomes of multiple organs, physiological functions in different organs can be further explored. Identifying the genes expressed prominently in the lung may reveal its unique physiological functions in the respiratory system.

The expression of some individual genes in the lung and other organs may be found in literature and public databases. In literature, newly discovered genes have been tested in various organs at the mRNA level with Northern blotting and RT-PCR and at the protein level with Western blotting. In public databases, gene expression are compiled from literature, cDNA library (e.g. UniGene) and high throughput tools such as serial analysis gene expression (SAGE) and DNA microarray (e.g., GEO) [1]. Several studies using DNA microarray have been reported for profiling differential gene expression among normal human and mouse organs, but very little information is available for the rat [2-6].

Dual color hybridizations are commonly used for differential expression of thousands of genes between two samples [7]. For three or more samples, a reference or loop design has to be employed to adapt dual color hybridization [8,9]. In the reference design, several samples are hybridized onto different slides separately with a common reference, which is prepared by pooling all the samples or using genomic DNA [10]. In the loop design, samples are paired in a loop pattern for hybridization and each sample is hybridized twice. However, the efficiency and reproducibility of both designs are poor for the identification of organ-prominent genes. Only two samples are hybridized on one slide, and the hybridization on different slides is known to have high variations due to slide printing and hybridization conditions [7]. For instance, there are 15 pair-wise combinations among 6 distinct organs. Consequently, 15 co-hybridizations between samples are required for a single replication and 60 slides for an experiment with 4 biological replications.

To eliminate these problems, we developed a parallel hybridization system in which 6 samples can be hybridized onto one single slide. This technique simplifies the investigation of multiple samples, reduces experimental errors and improves experimental efficiency. Using this system, we investigated lung-prominent genes by comparing gene expression profiles among rat lung, heart, kidney, liver, spleen, and brain. The lung-prominent genes identified in the present study may provide a clue for further exploration of pulmonary functions.

## Results

### Reproducibility and efficiency of parallel hybridization

Our parallel hybridization system consists of three identical blocks: A, B, and C, on a single slide (Table 1). Each block contains ~10,000 50-mer oligonucleotides (6,221 known rat genes, 3,594 rat ESTs, and 169 Arabidopsis negative controls). Six labeled cDNA samples (3 Cy3 and 3 Alexa 647) were combined into 3 green-red pairs and hybridized onto each block of one slide. During the hybridization step, the blocks were separated by thermostatic tapes. The latter was removed during the washing and scanning steps. To examine whether there was cross-contamination among blocks, blocks A and C on the same slide were hybridized simultaneously for 3 days with Alexa 647-labeled lung cDNA. No signals were detected in block B (data not shown), indicating no cross contaminations among blocks.

**Table 1:** Slide layout and hybridization design

Slide	Block A		Block B		Block C	
	
	Green	Red	Green	Red	Green	Red
1	Lung	Heart	Liver	Brain	Kidney	Spleen
2	Heart	Kidney	Liver	Lung	Spleen	Brain
3	Kidney	Liver	Lung	Brain	Spleen	Heart
4	Heart	Brain	Kidney	Lung	Liver	Spleen
5	Heart	Liver	Lung	Spleen	Brain	Kidney

The array has three identical blocks, A, B, and C; each containing 9,984 spots representing 6,221 known rat genes, 3,594 ESTs, and 169 Arabidopsis negative controls. The three blocks are separated with thermal plastic rings. Three paired Cy3-(green) and Alexa 647 (red)-labeled cDNA samples were hybridized onto three blocks, A, B, and C of slide 1–5. Dye and sample assignments are random for each slide. Five slides represent technical replications.

**Figure 1:**
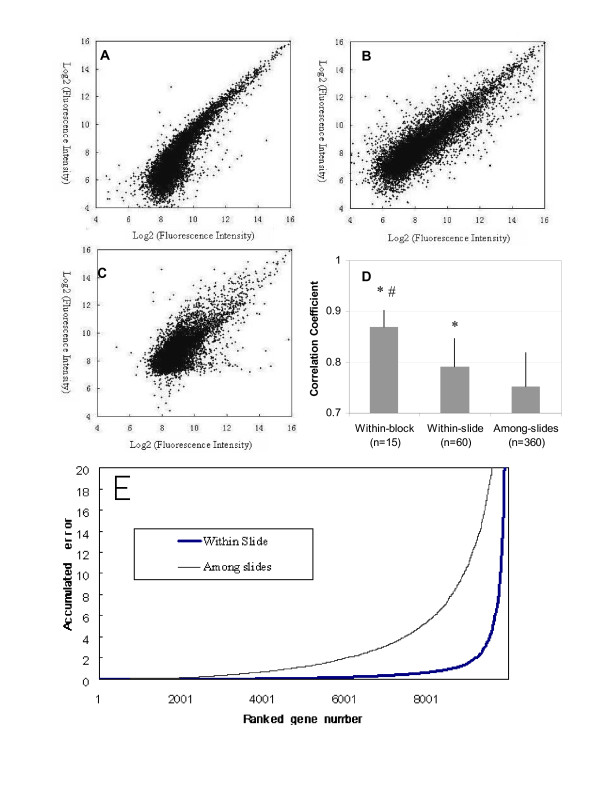
**Reproducibility of hybridizations**. (A-C):typical scatter plots of self-self hybridization of lung cDNAs between two channels within a block (within-block, panel A), two different blocks in one slide (within-slide, panel B), and among slides (among-slide, panel C), respectively. The cDNAs from an identical lung tissue were labeled with Cy3 or Alexa 647, and hybridized to each block of the slides. The numbers on x- and y-axis were background-subtracted fluorescence intensities of each spot with log2 transformation. (D) A comparison of correlation coefficients from replicated hybridizations. The results were expressed as means ± SE. *P < 0.01 v.s. among-slide; ^#^P < 0.01 v.s. within-slide. (E) Comparison of accumulated errors between within-slide and among-slide groups. For the within-slide group, the log ratios were from parallel hybridization on a single slide. For the among-slides, the log ratios were from different slides. The accumulated errors were calculated as described in *Materials and Methods*.

Self-self hybridizations were performed on three slides to assess the reproducibility of hybridizations using Cy3- and Alexa 647-labeled lung cDNA samples. We observed the highest correlation coefficient between two samples co-hybridized in one block (within-block group, Fig. 1A), and the lowest one between two samples hybridized in two different blocks on two separate slides (among-slide group, Fig. 1C). The within-slide group (two samples in two distinct blocks on one slide, Fig. 1B) possessed a significantly higher reproducibility than the among-slide group, but lower than the within-block group (Fig. 1D, p < 0.01). The lower reproducibility of the among-slide group may be due to the experimental variations among slides, such as hybridization temperature fluctuation, washing, and scanning. These conditions were identical for the within-block and within-slide groups, in which samples were hybridized in a single slide.

Next, we investigated the relative gene expression levels in 6 rat organs: lung, heart, kidney, brain, spleen, and liver. The hybridization of each organ was repeated 20 times: 4 biological replications (rats), each with 5 technical replications (slides). Six samples from each of four rats were split into 5 aliquots for hybridization on 5 slides. The labeling dyes, the sample pairing, and the hybridization blocks on a slide were randomly assigned for each biological replication. This minimized the variations among biological and technical replications, including animals, fluorescence dyes, sample combinations, blocks on a slide, slides, and experimental conditions (Table 1). Statistically, each slide was a random block containing 6 samples. There were 60 sample-sample hybridizations performed on 20 slides (60 Alexa 647-cDNA and 60 Cy3-cDNAs) in this experiment. To achieve similar statistical results, a traditional reference design requires 120 slides for co-hybridizations of sample and reference. Alternatively, in a loop design, 60 slides are required for co-hybridization of sample-sample.

The difference of fluorescence intensity between the parallel hybridization and traditional dual-color hybridization was evaluated. We first compared the difference of log ratios between the traditional and parallel hybridization systems by SAM [11]. The samples of lung and heart were used as an example. The log ratios of fluorescence intensity between lung and heart were normalized with the print-tip based LOWESS [7]. The traditional log ratios were from 4 slides, in which lung and heart were paired and co-hybridized onto the same block of each slide. The parallel log ratios were from 4 other slides, in which lung and heart were hybridized onto two different blocks of each slide. The 2-class SAM test identified no genes that showed a significant difference between the traditional co-hybridization group and the parallel hybridization group (false discovery ratio<0.047, q-value>0.05). Other organ pairs showed similar results. These results demonstrated that the log ratios of two samples from two different blocks in the parallel hybridization were not significantly different from that of the traditional two sample co-hybridization. Consequently, any two of the six samples hybridized onto one slide in the parallel hybridization can be directly compared as if these samples were pair-wise combined and co-hybridized onto one traditional slide.

We also tested the accumulated error of the log2 ratios among 6 organs. In a traditional loop design, the sum of log ratios along the loop should be zero, but frequently fluctuating. Therefore, the square sum of log ratios can be adapted to assess the accumulated error of each gene or the data fluctuation in one experiment. We selected one block from each of the six different slides and simulated the traditional loop design. The 6 blocks formed a loop as if they were 6 traditional co-hybridization slides. In another group, a loop was formed from a single parallel hybridization slide. The slides for both groups were randomly selected. The accumulated errors were calculated as described in the *Materials and Methods*, followed by being sorted ascendingly, and plotted against ranked genes. We found that 21% of the genes showed an accumulated error of >5 in the traditional hybridization group, but only 4% in the parallel hybridization group (Fig. 1E). A paired t-test of the accumulated errors between the two groups revealed that the fluctuation of the traditional co-hybridization was significantly higher than that of the parallel hybridization (p < 0.05).

### Prominent genes expressed in the lung

**Figure 2:**
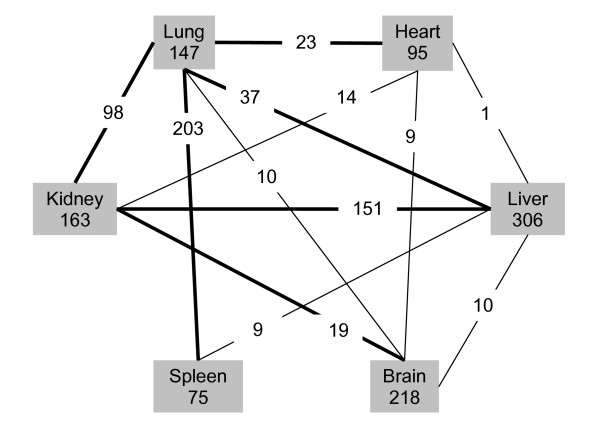
**Summary of differentially expressed genes among 6 organs**. The number under an organ represents the genes that are expressed significantly higher in the respective organ compared to other organs (p < 0.05). Similarly, the number between any two organs represents the genes that are expressed significantly higher in the two organs compared to other organs (p < 0.05). Thicker lines highlight a larger number of the genes co-expressed in the respective two organs.

**Figure 3:**
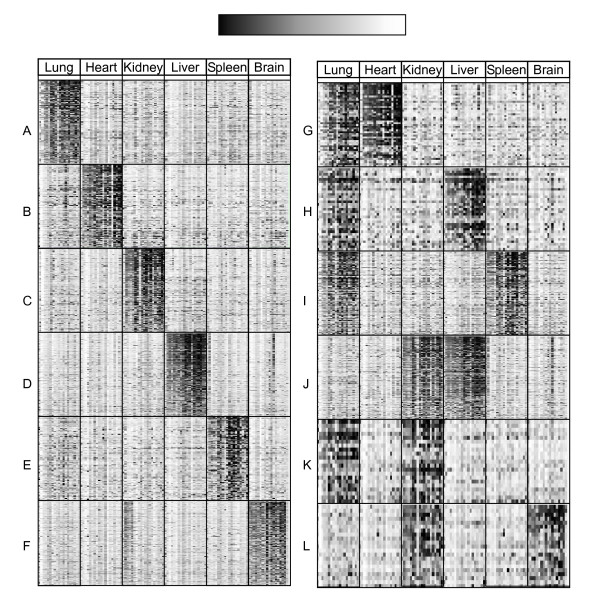
**Hot maps of Organ-prominent genes**. Left and right panels are the relative expression levels of genes differentially expressed in one and two organs, respectively. Each column represents 19 replicated hybridizations of each organ and each row shows the spot signals of the organ-prominent genes. The scale of normalized spot signals was indicated on the top of the graph. (A): lung: 166 genes; (B) heart: 100 genes; (C) kidney: 186 genes; (D) liver: 324 genes; (E) spleen: 88 genes; (F) brain: 225 genes; (G) lung-heart: 47 genes; (H) lung-liver: 33 genes; (I) lung-spleen: 95 genes; (J) kidney-liver: 174 genes; (K) lung-kidney: 21 genes; (E) kidney-brain: 21 genes.

Lung-prominent genes were identified through quality filter, statistics filter, and image confirmation. Several steps of data analysis were followed (see Materials and Methods for details): (i) After hybridization, we first checked the qualities of whole hybridization images and excluded the images from poor slides (one out of 20 slides was discarded); (ii) We filtered 2,829 low quality spots based on a mean quality index of <1 as our quality filter; (iii) Statistics test using SAM analysis revealed that the expression levels of 3,576 genes were significantly different among 6 organs (false-positive ratio <5%, and median false discovery ratio <0.05); (iv) In order to identify organ-prominent or co-expressed genes, the genes passed SAM test were further analyzed by multiple comparisons using Turkey's honestly significant difference (HSD) tests at an overall confidence level of 95%. Organ-prominent genes are defined as genes that are expressed significantly higher in one particular organ than any other organs (P < 0.05). Similarly, co-expressed genes are the genes that are expressed significantly higher in two organs than any other 4 organs (P < 0.05). There were some duplicated genes in single and two organ-prominent groups. The duplicated genes with a lower OSI were filtered. The duplication was due to the HSD-based multiple comparisons. For instance, endothelial cell growth factor protein precursor (VEGF, Genbank ID: NM_031836) was expressed significantly higher in the lung than other organs (p < 0.05, OSI for lung = 0.975). This gene was also co-expressed significantly higher in the lung and the liver than in other organs (p < 0.05, OSI for lung and heart = 0.778). In this case, we thus deleted this gene from the lung-liver group; (v) Finally, we further verified the genes identified above by directly comparing the results with spot images in a spreadsheet using the *RealSpot *software [12]. The visually inconsistent genes with spot images were filtered. The final genes were summarized in Fig. 2 and the hot maps of these genes were shown in Fig. 3. The liver showed the highest number of prominent genes (306 genes) and spleen the lowest (75 genes). The numbers of other organ-prominent genes were brain (218), kidney (163), lung (147), and heart (95). The lung had a high number of co-expressed genes with other organs: lung-spleen (203), lung-heart (23), lung-liver (37), lung-kidney (98), and lung-brain (10). The kidney also had a high number of co-expressed genes, kidney-liver (151) and kidney-brain (19). A list of all the organ-prominent genes is given in Additional file 1 (Supplementary Table E1, the excel format to show gene expression data) and Additional files 2 and 3 (Supplementary Table E2A and E2B, the PDF format to show spot images).

The prominent genes for one or two organs were further classified into 4 functional categories: function unclear, cellular location, molecular function, and biological process, using ontology annotations from Rat Genome Database  and Gene Ontology . The main functional categories of prominent genes were summarized in Additional file 4 (Supplementary Table E3) for one organ and in Additional file 5 (Supplementary Table E4) for two organs. The functions of the lung-prominent genes include ligand binding, signal transducer, cell communication, development, and metabolism. The cellular location was omitted since only a few genes were documented at the sub-cellular level. It is worthy to note that the functions of 60% or more genes we identified remain unclear in the present time.

### Real-time PCR verification

**Figure 4:**
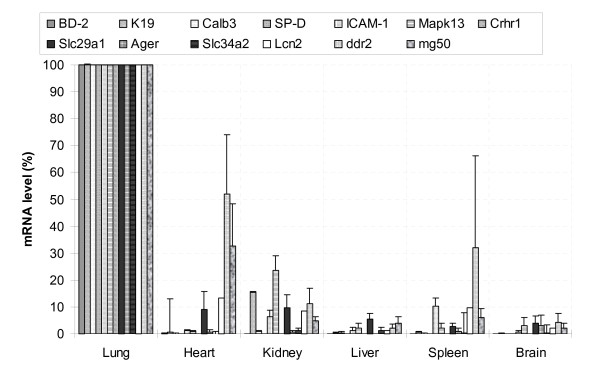
**Relative mRNA abundance of lung-prominent genes determined by relative real-time PCR**. The mRNAs from six organs were reverse-transcribed to cDNA and quantified by relative real-time PCR. All of the genes were run on the same plate with 18S rRNA as an endogenous reference. The results were expressed as % of lung. Data shown are means ± S.E. (n = 3 biological replications).The mRNA expression level of all the genes in the lung was significantly higher in other organs (P < 0.05).

Based on our research interests, we focused on lung-prominent genes for real-time PCR verification. We selected genes based on both mRNA abundance (signal intensity) and organ specificity index (OSI). OSI was defined as the correlation coefficient of expression levels between an interested gene and a putative gene that had 100% specificity (see *Materials and Methods*). The known lung marker genes have high OSIs, e.g. T1α, 0.996; SP-A, 0.993; SP-D, 0.993; SP-B, 0.933; CCSP, 0.972; and SP-C, 0.912 (Additional file 1). We chose 13 genes, which ranked in the top 30% in signal intensity (high expression level) and the top 10% in OSI (high specificity). In addition, we selected 3 genes that ranked below 30% in signal intensity (low expression level). Real-time PCR verified 13 genes that were expressed significantly higher in the lung than in other organs (Fig. 4). These genes include BD-2, K19, Calb3, SP-D, ICAM-1, Mapk13, Crhr1, Slc29a1, Ager, Slc34a2, Lcn2, Ddr2, and Mg50. Furthermore, the expression level for most of the genes in the lung was 10 times or more greater than that in other organs. The expression pattern of these genes was consistent with DNA microarray signals (Additional file 6). Three genes, Nup155, MMP9 and Sp4, did not show a significantly higher mRNA abundance in the lung when compared to other organs under our experiment conditions. This is due to high variations between samples.

## Discussion

In the current study, we developed a parallel hybridization, in which 6 samples can be hybridized onto one single slide. This method provides higher reproducibility and efficiency than the standard co-hybridization, and should be suitable for experiments investigating multiple biological samples. Using this system, we identified genes prominently expressed in one or two organs of the rat lung, heart, kidney, liver, spleen, and brain. Thirteen out of 16 selected lung-prominent genes were verified by real-time PCR. The genes identified in present study may be useful for further functional investigation in the lung or other organs.

The organ-prominent genes we identified were directly based on statistical comparisons of normalized spot signals. These genes were further ranked by organ specificity index (OSI). The "standard" DNA microarray data process extracts fluorescence intensities of both channels from hybridization images, and calculates and normalizes ratios for further statistical analysis. Our method is different from the "standard" analysis in several ways: (i) we linearly transformed all of the spot signals from each channel of hybridization images into a 0–1,000 scale, which made different channels and slides comparable. Unlike the ratio normalization, we retained relative expression levels in each channel. This is especially useful for multiple sample comparisons; (ii) Gene classification was based on multiple comparison. Differentially expressed genes among the 6 organs were identified from SAM test, followed by multiple comparison using Tukey's HSD; and (iii) we ranked the genes by organ specificity index (OSI), higher OSI, more specific a gene in one or two organs. In this investigation, we selected lung-prominent genes for verification based on the combination of OSI and normalized spot intensity. We chose the genes ranked in the top 10% in OSI and the top 30% in spot intensity, which ensures both the lung-specificity and the gene expression level.

Recently, several studies have compared gene expression profiles in human and mouse [2-6]. Only one report was done on rats with a focus on the brain using commercial Affymetrix chips (7,000 known genes and 1,000 EST) [6]. In this data set, the lung and liver were not included and only two replications for the spleen, heart, and kidney were used. In comparison, 2,426 genes out of the 3,576 differential genes (current study, without image-filter) were found to be common with the Walker's study [6]. The correlation coefficient of relative expression between the two data sets was around 0.4 for heart, kidney, or spleen. The low quantitative correlation may be due to the differences between Affymetrix and our in-house microarray platforms such as glass slide/silicon wafer, two/one channel, and 50-mer oligonucleotide/25-mer oligonucleotide set. However, the two data sets showed a consistent gene expression pattern among heart, kidney, and spleen, when we manually compared differential expression of the genes with top OSI for each pattern.

We also compared our dataset with the published datasets from other species. In the Novartis GNF dataset, transcriptomes of mouse organs were compared, each organ with duplicated single channel hybridizations [5]. Of the 147 lung-specific genes in our dataset, 102 were found in the mouse microarray dataset (totally 31,770 genes). Based on OSI>0.75, calculated from their dataset, we found that 36 lung-prominent genes are common with our dataset. Six of them were on the list of our 13 real-time PCR-verified lung-prominent genes, including Ager, K19, SP-D, ICAM-1, Slc34a2, and Lcn2. Another verified gene, MAPK13 was not in the 36 genes. Its signals were less than 50 in all of the mouse organs.

We further compared our dataset with available human datasets. The dataset  and  listed 43 human lung-specific genes. Many known lung-specific genes such as T1α, caveolin and CCSP were not on this list. Among 43 genes on the list, 18 genes were found in our list of lung-prominent genes, including known lung-specific genes, surfactant proteins, ager and a verified gene, Slc34a2. Similarly, in another human tissue dataset (PubmedID: **15774023**), 50 lung-specific genes were identified based on one human lung tissue hybridization. Once again surfactant proteins and ager were not in list. The only common gene between the list and our lung-prominent genes was caveolin. Finally, when comparing our rat dataset (10 K genes) and the Novartis GNF dataset human (10 K genes) datasets, we found 368 common genes between the datasets. Only 2 common genes, MAPK13 and latent transforming growth factor beta binding protein 2 (LTBP2), appeared to be lung-prominent based on the OSI. There were more common genes between the rat and the mouse datasets than these between the rat and the human datasets.

The published dataset was based on one or two hybridization of normal lung tissue. Our lung-prominent genes were based on 20 replicated DNA microarray hybridizations (4 biological and 5 technical replications). We believe that our gene lists were statistically confident and had a lower false-positive or false-negative genes.

The 13 lung-prominent genes we verified by real-time PCR have various functions, including pulmonary defenses, ion/solute transport, hormone receptor, differentiation, oxidant response and tumorgenesis. Five of them are defense genes. BD-2 (β-defensin 2) is a cationic peptide with a broad-spectrum antimicrobial activity and contributes to innate immunity in the lung [13]. It is expressed in the airway epithelia [14]. BD-2 was increased in the patients with inflammation and infections [15,16]. SP-D (surfactant protein D) is highly expressed in alveolar epithelial type II cells and plays a pivotal role in cell defense against microbes [17,18]. For instance, it has been reported that SP-D inhibited the proliferation of bacteria by increasing the permeability of the microbial cell membrane. ICAM-1 (intercellular adhesion molecule 1) is a cell adhesion molecule and a ligand for leukocyte adhesion molecule LFA-1. ICAM-1 also participates in the inflammatory response to lipopolysaccharide-induced lung injury by interacting mainly with neutrophils [19]. Lipcocalin 2 (also known as α_2u_-globulin-related protein, X13295) is a member of lipocalin protein family composed of small secreted proteins that have the ability to bind to small hydrophobic ligands [20]. Lipocalin 2 expression in the lung is markedly increased in acute lung injury caused by diesel exhaust particles and lipopolysaccharide [21]. Mapk13 (mitogen activated protein kinase 13) plays a role in stress and inflammatory responses via the MAPK cascade signaling pathway. Mapk13 is predominantly expressed in the lung although a small amount of Mapk 13 is also present in kidney [22], which is consistent with our results (Fig. 4).

Three of the identified lung prominent genes are ion/solute transporters. Calb3 (Calbindin 3), a vitamin D-dependent Ca^2+ ^binding protein, was previously studied in the intestine, uterus, placenta, and lung epithelium [23]. It is a Ca^2+ ^transporter and regulates Ca^2+ ^homeostasis. Slc29a1 (solute carrier family 29 transporter, member 1) is an equilibrative nitrobenzylthioinosine-sensitive nucleoside transporter (ENT1), which transports nucleosides into or out of the cells in a Na^+^-independent manner [24]. Northern blot analysis has shown that Slc29a1 is highly expressed in the lung and testes [25]. It plays a role in nucleotide biosynthesis and cellular signaling. Slc34a2 (solute carrier family 34 sodium phosphate, member 2) is a sodium dependent phosphate transporter. It has been shown that Slc34a2 was predominantly expressed in the lung and in situ hybridization revealed that it is localized in alveolar type II cells [26]. Slc34a2 provides inorganic phosphate for the synthesis of lung surfactant.

Crhr1 (corticotropin releasing hormone receptor 1) is a receptor that binds corticotropin-releasing hormone. The mice null for the CRFR1 gene died within 48 hours after birth because of a pronounced lung dysplasia [27]. Interestingly, variation of Crhr1 was associated with improved function in the asthma patients who were treated with inhaled corticosteroids [28]. K19 (keratin 19) is expressed in epithelial cells, involved in testicular differentiation and lung cancer [29,30]. Ager (advanced glycosylation end product-specific receptor) is a member of the immunoglobin superfamily and is involved in oxidant response. It is specifically expressed in alveolar epithelial type I cells [31]. Lung type I cells are squamous, covering >90% of alveolar surface, and, thus, are easily damaged by oxidants. Ager may protect lung type I cells from oxidative injury.

The two lung-prominent genes with lower mRNA abundance, Ddr2 and Mg50, may be involved in human tumorgenesis , and the regulation of collagen remodeling in the lung .

The functions of 13 verified genes as well as some highly abundant co-expressed genes in the lung and another organ were summarized in Table 2. These co-expressed genes were previously studied in the lung or another organ. The most prominent genes expressed in the lung were relevant to pulmonary protection, including oxidant response, injury and repair, inflammatory, cell defense, and immune response. These genes also contribute to organ construction such as lung veins, energy supply, and epithelial tight junction. Some of these genes may be important for cell proliferation, such as anp and nf2. Two genes, anp and aqp5 may play a role in asthma and edema, respectively. The function of cd37 is currently unclear in any of the organs. Its prominent and specific expression in the lung may imply its important role for lung function. Cd37 may participate in cell proliferation in the lung based on the studies from other members of this gene family. Similarly, cathepsin Y may play a role of surfactant protein processing or apoptosis considering its endopeptidase activity in the spleen and the functions of cathepsin D and H in the lung. These hypothesized functions may serve as a starting point for further functional studies in the respective organs.

**Table 2:** Gene functions in the lung and 2^nd ^organ

Gene	Function in lung	2^nd ^organ (location)	Function in 2^nd ^organ
Ager	Oxidant response	(AEC I)*	
ICAM-1	AEC-leukocyte adhesion	(AEC)	
K19	Cell differentiation	(AEC)	
SP-D, BD-2	Defense, surfactant	(AEC II)	
slc42a2	*Surfactant synthesis?*	(AEC II)	
Calb3	Ca^2+ ^homeostasis		
Mapk13	Inflammatory response		
Slc29a1	*Ion transporter?*		
Lcn2	*Apoptosis?*		
Crhr1	*Hormone receptor?*		
Ddr2	Collagen remodeling		
Mg50	*Tumor pathogenesis?*		
Tnni2, tni3	Lung veins [35]	Heart	Muscle contrast [36;37]
Cox6a2, Cox8h	*Energy supply?*	Heart	Muscle energy supply [38] [39]
Anp	*Asthma? *[40]	Heart	Proliferation control [41]
Aqp5	*Edema?*	Liver	Fluid homeostasis [42]
Ces3, gpt	Injury and repair [43] [44]	Liver	Injury [45]
Cyp2615	Oxidantive stress [46]	Liver	*Xenobiotic metabolism?*
Cldn3	Epithelia barrier [47]	Liver	Paracellular permeability [48]
S100a18	Cell migration [49;50]	Spleen	*Cell motility?*
Iga, Igm [51] [52]	Immune response	Spleen	Immune response
Cd37 [53]	*Proliferation?*	Spleen	*Proliferation?*
Cathepsin Y	*Surfactant process? *[54] [55]	Spleen	Endopeptidase [56;57]
Fas, Alp	AEC II injury [58]	Kidney	Renal injury [59] [60]
Tpa66	Inflammatory [61]	Kidney	Anti-arterial thrombosis [62]
Nf2 [63]	Tumor supression	Kidney	Tumor suppression

* AEC I and II: Alveolar epithelial type I and II cells. See the main text for more references.

? Hypothesized function

The parallel hybridization system has several advantages over the traditional two-color hybridization. First, in this hybridization system 6 paired and dual-color labeled samples were hybridized onto one slide and scanned under identical conditions. The homogenous conditions on one slide improved the reproducibility and decreased the variation, especially accumulated experimental errors. The latter is problematic in microarray experiments involving a series of samples such as a time course study. Second, any two of the six samples in a parallel hybridization can be directly compared, whereas only two paired samples can be directly compared in the traditional two-color hybridization of a reference or loop design. This increases the experimental efficiency and reduces the number of slides and the amount of RNAs in a whole experiment. In the parallel hybridization system, only one slide is needed for six samples. In contrast, 6 slides are required for a reference or loop design of six samples in the traditional two-color co-hybridization. The RNA amount is reduced to half that of the traditional hybridization. This is because each sample needs to hybridize twice with neighboring samples in the loop design or hybridize to a common reference consisting of all the samples in the reference design. Multiple-color hybridization on one slide could be developed for three or more samples labeled with distinct fluorescence dyes. However, the potential cross-talk among fluorescence dyes and the need for multiple lasers of a scanner limit its application.

The organ-prominent genes in the current study were identified from 6 organs. Some of them may be expressed higher in other tissues outside the 6 organs we monitored. This limitation may be overcome by further improvement of the parallel hybridization system. One possibility is to include one common control organ (e.g. lung) in all of the parallel hybridization slides. Although it reduces the efficiency, the transcriptomes of more than 6 organs can be directly compared. Another possibility is the potential technical improvement of spot printing and sample arrangement, which may result in more than 6 samples on one parallel slide. In the present study, we printed 10 K rat genes in triplicate on three blocks on one slide. Each block contains 16 sub-arrays (4.5 × 4.5 mm) consisting of 625 genes. Therefore, 6 samples can be hybridized to 10 K genes in this system. If we print 625 genes onto 48 sub-arrays in replicate, 96 dual-color labeled samples can be hybridized on one slide. Furthermore, if we increase the printing resolution from 160 to 80 microns, we can print 2,500 spots on one sub-array. Consequently, 96 samples can be hybridized to one slide containing 2,500 genes. Another improvement may be the separation of the slide regions. We used thermostatic tapes to divide 3 blocks, which may not be appropriate for more samples. The chambered coverslips of 24 or 48 wells such as CultureWell™ coverslip system or array of arrays glass wafer [32] may be adapted for this purpose.

## Methods

### Microarray preparation

The DNA microarray slides used in this study were in-house printed on epoxy-coated glass slides with 50-mer aminated oligonucleotides, Pan Rat 10 K Oligonucleotide Set (MWG Biotech Inc., High Point, NC). It contains 6,221 known rat genes, 3,594 rat ESTs, and 169 Arabidopsis negative controls. The oligonucleotides were suspended in 3× SSC at 25 μM and printed on epoxy-coated slides (CEL Associates, Pearland, Texas) with an OmniGrid 100 arrayer (GeneMachine, San Carlos, CA) per manufacturer's instructions. Each oligonucleotide was spotted in triplicate on three identical 18 × 18 mm blocks: A, B, and C (Table 1). The total spots on one slide were 30,000 including 186 blank spots. The spot-spot distance was 180 μm and the space between blocks was 4 mm. The printed slides were incubated in 65% humidity overnight at room temperature. The slides were then dried and stored in room temperature. Prior to hybridization, the slides were washed one time with 0.2% SDS, four times with water, and dried by centrifugation. The 3 blocks on a slide were separated by two 2 × 25 × 1 mm thermostatic transparent tape stripes during the hybridization. The stripes were removed after hybridization to wash and scann slides.

### Sample collection and hybridization

Six organs, the lung, heart, kidney, liver, spleen, and brain of male Sprague-Dawley rats (200 g, Charles River Laboratories, Inc., Wilmington, MA) were dissected. The organs were briefly washed with deionized water and immediately homogenized in 10 ml TRI reagents (Molecular Research Center, Cincinnati, OH). Total RNA was subsequently extracted according to the manufacturer's protocol. RNA quality and quantity were assessed by spectrophotometer (NanoDrop Technologies, Inc, Rockland, DE) and agarose gel electrophoresis. Total RNA samples were aliquoted (20 μg each) for cDNA synthesis and 2-step microarray hybridization with 3DNA 50 Expression kit (Genisphere Inc., Hatfield, PA). Briefly, total RNA was reverse-transcribed with Cy3- or Alexa 647-specific primers. The cDNA products were purified with the Microcom YM-30 columns (Millipore, Billerica, MA) and mixed with 2× formamide hybridization buffer (50% formamide, 6× SSC, 0.2% SDS). The DNA microarray slides were hybridized with the cDNA samples at 42°C for 48 hours. The slides were washed and re-hybridized with Cy3- and Alexa 647-specific capture reagents at 42°C for 2 hours. In our experiments, the concentration of purified cDNA samples were normalized to 0.5 – 0.6 μg/μl before hybridization. The cDNA aliquots from 6 organs of the same rat were randomly paired and independently hybridized onto one of 3 blocks on a glass slide. Each sample was repeated 20 times: 4 biological replications and 5 technical replications. The arrangement of samples, fluorescence dyes, and blocks for one of the biological replications is shown in Table 1. The other 3 biological replications were similarly arranged in a style of random block design. Each hybridized slide was scanned twice by a laser confocal scanner, ScanArray Express (PerkinElmer Life and Analytical Sciences, Boston, MA). The first scanning was used for quantification and performed with 90% laser power and 70~80% PMT so that about 5% spots were saturated. The second scanning was used for spot alignment and was carried out with 90% laser power and 95% PMT. Hybridization images were analyzed with GenePix pro 4 (Axon Instruments, Inc. Union City, CA).

### Data analysis

#### Hybridization reproducibility

The reproducibility was assessed by Pearson correlation coefficients of spot signals from self-self hybridizations. The spot signals were background-subtracted fluorescence intensity extracted from hybridization images by GenePix. To estimate the variations among 6 paired organs, accumulated errors of log ratios were calculated. The log ratios between two samples were assessed from the respective spot signals, normalized by local weighted scatter plot smooth (LOWESS) based on print-tip. The accumulated error of ratios of each gene was assessed as (1)

e=(log⁡'s1s2+log⁡'s2s3+log⁡'s3s4+log⁡'s4s5+log⁡'s5s6+log⁡'s6s1)2     (1)
 MathType@MTEF@5@5@+=feaafiart1ev1aaatCvAUfKttLearuWrP9MDH5MBPbIqV92AaeXatLxBI9gBaebbnrfifHhDYfgasaacH8akY=wiFfYdH8Gipec8Eeeu0xXdbba9frFj0=OqFfea0dXdd9vqai=hGuQ8kuc9pgc9s8qqaq=dirpe0xb9q8qiLsFr0=vr0=vr0dc8meaabaqaciaacaGaaeqabaqabeGadaaakeaacqWGLbqzcqGH9aqpdaqadaqaaiGbcYgaSjabc+gaVjabcEgaNjabcEcaNmaalaaabaGaem4CamNaeGymaedabaGaem4CamNaeGOmaidaaiabgUcaRiGbcYgaSjabc+gaVjabcEgaNjabcEcaNmaalaaabaGaem4CamNaeGOmaidabaGaem4CamNaeG4mamdaaiabgUcaRiGbcYgaSjabc+gaVjabcEgaNjabcEcaNmaalaaabaGaem4CamNaeG4mamdabaGaem4CamNaeGinaqdaaiabgUcaRiGbcYgaSjabc+gaVjabcEgaNjabcEcaNmaalaaabaGaem4CamNaeGinaqdabaGaem4CamNaeGynaudaaiabgUcaRiGbcYgaSjabc+gaVjabcEgaNjabcEcaNmaalaaabaGaem4CamNaeGynaudabaGaem4CamNaeGOnaydaaiabgUcaRiGbcYgaSjabc+gaVjabcEgaNjabcEcaNmaalaaabaGaem4CamNaeGOnaydabaGaem4CamNaeGymaedaaaGaayjkaiaawMcaamaaCaaaleqabaGaeGOmaidaaOGaaCzcaiaaxMaadaqadaqaaiabigdaXaGaayjkaiaawMcaaaaa@74A6@

Where e is the accumulated error, and log⁡'sisj
 MathType@MTEF@5@5@+=feaafiart1ev1aaatCvAUfKttLearuWrP9MDH5MBPbIqV92AaeXatLxBI9gBaebbnrfifHhDYfgasaacH8akY=wiFfYdH8Gipec8Eeeu0xXdbba9frFj0=OqFfea0dXdd9vqai=hGuQ8kuc9pgc9s8qqaq=dirpe0xb9q8qiLsFr0=vr0=vr0dc8meaabaqaciaacaGaaeqabaqabeGadaaakeaacyGGSbaBcqGGVbWBcqGGNbWzcqGGNaWjdaWcaaqaaiabdohaZjabdMgaPbqaaiabdohaZjabdQgaQbaaaaa@3746@ are normalized log ratios between samples s1~s6, the 6 organs arranged in a loop design. The e was calculated in 2 groups, within-slide group and among-slide group. The log ratios of within-slide group were obtained from one slide with 6 samples, and those of among-slide group were from 6 slides comprised of a loop design for 6 samples.

#### Identification of lung-prominent genes

To identify differentially expressed genes among 6 organs, we first globally normalized 16-bit mean fluorescence intensity of each gene from original images using the software *RealSpot *developed in our laboratory [12] (freely available for download for academic usage, ). The global normalization converted the weakest 5% fluorescence intensities to 0 (background) and the strongest 5% fluorescence intensities to 1,000 (saturated spots, reflecting normally scanned images). The other fluorescence intensities were scaled to the range of 0 to 1,000. This transformation makes different slides and different channels comparable. It is similar to Affymetrix single channel data normalization. The transformed images and intensities were used for data quality filters, statistics tests, and direct confirmation of the data analysis results with spot images.

For spot quality evaluation, a quality index (QI) was assigned to each spot based on signal intensity and signal-to-noise ratio. QI 0–4 indicate empty, weak, middle, strong, and saturated spots, respectively. By default, QI 0 and 4 were assigned to the empty and saturated spots, whose intensities were less than 30% and greater than 95%, respectively. QI 1–3 was calculated, based on the intensity of spot signals, as:QIij=round(Iij−I0I1−I0∗4)
 MathType@MTEF@5@5@+=feaafiart1ev1aaatCvAUfKttLearuWrP9MDH5MBPbIqV92AaeXatLxBI9gBaebbnrfifHhDYfgasaacH8akY=wiFfYdH8Gipec8Eeeu0xXdbba9frFj0=OqFfea0dXdd9vqai=hGuQ8kuc9pgc9s8qqaq=dirpe0xb9q8qiLsFr0=vr0=vr0dc8meaabaqaciaacaGaaeqabaqabeGadaaakeaacqWGrbqucqWGjbqsdaWgaaWcbaGaemyAaKMaemOAaOgabeaakiabg2da9iabdkhaYjabd+gaVjabdwha1jabd6gaUjabdsgaKjabcIcaOmaalaaabaGaemysaK0aaSbaaSqaaiabdMgaPjabdQgaQbqabaGccqGHsislcqWGjbqsdaWgaaWcbaGaeGimaadabeaaaOqaaiabdMeajnaaBaaaleaacqaIXaqmaeqaaOGaeyOeI0IaemysaK0aaSbaaSqaaiabicdaWaqabaaaaOGaey4fIOIaeGinaqJaeiykaKcaaa@4A2C@, where QI_ij _is the quality index of spot j on slide i and *I*_ij _the intensity of the spot j on slide i. By default, *I*_0 _is the intensity at 30^th ^percentile, and *I*_1 _at 95^th ^percentile of the plot (intensity vs gene rank percentage) of the slide image. A QI of 5 was assigned to a contaminated or bad spot based on signal background ratio (SBR). By default, any spots with a SBR of <2.0 were given a QI of 5. A mean quality index was calculated from the replicated spots of a gene from multiple slides, excluding bad spots (QI = 5). Data were filtered if a mean quality index was 1.0 or less.

For the genes that passed the quality index filter, statistical tests were performed. The genes with a significantly differential expression among 6 organs for at least one organ-pair were identified by a software package, *SAM*, (Significant Analysis of Microarray, ) [11]. The median false discovery ratio (FDR) cutoff for a multiple class response test by SAM was set to 5%. The genes with a minimal FDR (q-value) of >5% were discarded. The genes that passed the SAM test were further classified into organ-prominent genes or co-expressed genes in two organs by pair-wise multiple comparisons with Tukey's honestly significant difference (HSD) at an overall confidence level of 95%. Organ-prominent genes were defined as the genes that were expressed significantly higher in one particular organ than in other organs (p < 0.05). Similarly, co-expressed genes in two organs were defined as the genes that were expressed in the two organs than the other 4 organs.

To determine the relative specificity of a gene among organs, an organ specificity index (OSI) was defined as the correlation coefficient of gene expression levels between a gene and a putative gene. The expression levels of a putative gene were 1,000 in prominent organs and 0 in other organs. For example, the expression level of a putative gene prominent in the lung will be (from left to right are lung, heart, kidney, liver, spleen, and brain) 1,000, 0, 0, 0, 0, 0. The OSI is calculated as

OSI=∑i=1n(Xi∗Pi)−∑i=1nXi∗∑i=1nPin∑i=1n(Xi2)−(∑i=1nXi)2n∑i=1n(Pi2)−(∑i=1nPi)2n     (2)
 MathType@MTEF@5@5@+=feaafiart1ev1aaatCvAUfKttLearuWrP9MDH5MBPbIqV92AaeXatLxBI9gBaebbnrfifHhDYfgasaacH8akY=wiFfYdH8Gipec8Eeeu0xXdbba9frFj0=OqFfea0dXdd9vqai=hGuQ8kuc9pgc9s8qqaq=dirpe0xb9q8qiLsFr0=vr0=vr0dc8meaabaqaciaacaGaaeqabaqabeGadaaakeaacqWGpbWtcqWGtbWucqWGjbqscqGH9aqpdaWcaaqaamaaqahabaGaeiikaGIaemiwaGLaemyAaKMaey4fIOIaemiuaaLaemyAaKMaeiykaKIaeyOeI0YaaSaaaeaadaaeWbqaaiabdIfayjabdMgaPjabgEHiQmaaqahabaGaemiuaaLaemyAaKgaleaacqWGPbqAcqGH9aqpcqaIXaqmaeaacqWGUbGBa0GaeyyeIuoaaSqaaiabdMgaPjabg2da9iabigdaXaqaaiabd6gaUbqdcqGHris5aaGcbaGaemOBa4gaaaWcbaGaemyAaKMaeyypa0JaeGymaedabaGaemOBa4ganiabggHiLdaakeaadaGcaaqaamaaqahabaGaeiikaGIaemiwaGLaemyAaK2aaWbaaSqabeaacqaIYaGmaaGccqGGPaqkcqGHsisldaWcaaqaaiabcIcaOmaaqahabaGaemiwaGLaemyAaKMaeiykaKYaaWbaaSqabeaacqaIYaGmaaaabaGaemyAaKMaeyypa0JaeGymaedabaGaemOBa4ganiabggHiLdaakeaacqWGUbGBaaaaleaacqWGPbqAcqGH9aqpcqaIXaqmaeaacqWGUbGBa0GaeyyeIuoaaSqabaGcdaGcaaqaamaaqahabaGaeiikaGIaemiuaaLaemyAaK2aaWbaaSqabeaacqaIYaGmaaGccqGGPaqkcqGHsisldaWcaaqaaiabcIcaOmaaqahabaGaemiuaaLaemyAaKMaeiykaKYaaWbaaSqabeaacqaIYaGmaaaabaGaemyAaKMaeyypa0JaeGymaedabaGaemOBa4ganiabggHiLdaakeaacqWGUbGBaaaaleaacqWGPbqAcqGH9aqpcqaIXaqmaeaacqWGUbGBa0GaeyyeIuoaaSqabaaaaOGaaCzcaiaaxMaadaqadaqaaiabikdaYaGaayjkaiaawMcaaaaa@9084@

Where Xi and Pi are the mean gene expression levels of each organ of a gene and the putative gene, respectively, in organ i. N is the total number of organs (n = 6 in this study). A higher correlation coefficient indicates a higher tendency of a gene for expression in a particular organ.

Finally, the gene expression data were directly compared with the respective spot images. The spot images of the genes in each sorted data set were searched and organized by *RealSpot*. The genes with visual consistence between differential gene expression and spot images were marked as highly prominent genes for the organ(s). The functional categories of these highly prominent genes were assessed based on gene ontology annotation from Rat Genome Database gene association file (RGD, ) and gene ontology definitions (GO, ).

### Real-time PCR

Selected lung-specific genes were validated by SYBR Green I based real-time PCR (QIAGEN, Foster City, CA) as previously described [33]. Total RNA (5 μg) was reverse-transcribed into cDNA with 0.2 μg/μl dT_17_, 0.3 μg/μl random hexamer primer, and MMLV reverse transcriptase (Invitrogen Inc., Carlsbad, CA). The primer pairs were as follows ("_F": forward, "_R": reverse): beta defensin-2, BD-2_F, AAT CAC ATG CCT GAC CAA AGGA; BD-2_R, GGA GCA AAT TCT GTT CAT CCCA; keratin19, K19_F, CCA GGT CGC TGT CCA CAC TAC; K19_R, CCT TCC AGG GCA GCT TTC AT; vitamin D-dependent calcium-binding protein, Calb3_F, CAG CAC TCA CTG ACA GCA AGCA, Calb3_R, TCC TCC TTG GAC AGC TGG TTT; surfactant protein D, SP-D_F, TTC TCT CCA TGC TTG TCC TGC T, SP-D_R, GAC TAG GGT GCA CGT GTT GGT T; intercellular adhesion molecule 1, ICAM-1_F, GGA GTC TCA TGC CCG TGA AAT, ICAM-1_R, GTG CCT ACC CTC CCA CAA CA; mitogen activated protein kinase 13, Mapk13_F, CCC AGC AGC CAT TTG ATG AT, Mapk13_R, CAC TGC AGC TTC ATC CCA CTT; corticotropin releasing hormone receptor, Crhr1_F, GGT CTC CAG GGT CGT CTT CAT C, Crhr1_R, ACG CCA CCT CTT CCG GAT AG; solute carrier family 29 transporters, member 1, Slc29a1_F, GGA CAA TGG TCT CTG ACG GAC A; Slc29a1_R, CCT GGA ACA GGC ACA GAA GAA A; advanced glycosylation end product-specific receptor, Ager_F, TCC GGT GTC GGG CAA CTA, Ager_R, GGG ACA TTG GCT GTG AGT TCAG; solute carrier family 34 sodium phosphate, member 2, Slc34a2_F, GCC CAT AGG TGT GAG CCT TTC, Slc34a2_R, CCC CAT TCA CTC CAT CCT AGG A; lipocalin 2, Lcn2_F, TCT GGG CCT CAA GGA TAA CAAC, Lcn2_R, AGA CAG GTG GGA CCT GAA CCA; matrix metalloproteinase 9, MMP9_F, TGG GCA TTA GGG ACA GAG GAAT, MMP9_R, GGG CTG TTT CCC CTG TGA GT; nucleoporin 155kd, Nup155_F, AAG TGG ATC AAA ACC GAG TTCG, Nup155_R, TCG CTG CTG CAG TGA AAT TTC; discoidin domain receptor family, member 2, Ddr2_F, AAC CAA GCA CCG ACC ATC CTT, Ddr2_R, ATG TGG CTG AGC GGT AGG TCT T; trans-acting transcription factor 4, Sp4_F, TTG TCA CAG TTG CCG CCA TT, Sp4_R, TGA CCA GCC CAT TTC CAG ATT T; melanoma-associated antigen, Mg50_F, TGC CAC ATC AGT CAC CCA TGA, Mg50_R, AGC CGA GAC TCC AGG CTG TTT A;18S rRNA_F: TCC CAG TAA GTG CGG GTC ATA, 18s rRNA_R: CGA GGG CCT CAC TAA ACC ATC. The real-time PCR thermal conditions for all 14 genes listed above were 95°C 15 min, followed by 40 cycles of 95°C for 30 sec, 60°C for 30 sec, 72°C for 30 sec, and 77°C for 35 sec. To eliminate experimental variations, all genes were amplified in the same plate, each with 6 organ cDNA samples from one rat (totally 84 wells for organ samples, other wells for negative controls). Three plates were used for the three biological replications. Data were analyzed using relative real-time PCR quantification based on the delta delta Ct method [34]. The endogenous reference gene was 18S rRNA, and the control organ was lung. One-way ANOVA tests were performed for statistical significance (p < 0.05).

## Authors' contributions

ZC performed the microarray experiments and data analysis and drafted the manuscript. JC and TW participated in the DNA microarray hybridization. NJ contributed to real time PCR. LL conceived of the study, and participated in its design and coordination and helped to draft the manuscript. All authors read and approved the final manuscript.

## Supplementary Material

Additional File 1 Supplementary Table E1, Organ-prominent genes in Excel format. Click here for file

Additional File 2 Supplementary Table E2A, One organ-prominent genes in PDF format with spot images. Click here for file

Additional File 3 Supplementary Table E2B, Two organ-prominent genes in PDF format with spot images. Click here for file

Additional File 4 Supplementary Table E3, Main functional categories of one organ-prominent genes. Click here for file

Additional File 5 Supplementary Table E4, Main functional categories of two organ-prominent genes. Click here for file

Additional File 6 Table 2: DNA microarray signal intensities and spot images of 13 verified genes Click here for file
