# Supplementary material for: Identification of rat lung – prominent genes by a parallel DNA microarray hybridization
Source: BMC Genomics. 2006 Mar 13;7:47. doi: 10.1186/1471-2164-7-47 (PMC1523215; doi:10.1186/1471-2164-7-47)
Supplement: Additional File 2 — Supplementary Table E2A, One organ-prominent genes in PDF format with spot images. [file 1471-2164-7-47-S2.pdf]

Meaning of columns:

Gene ID: Genbank accession number;

GeneName: name and common symbol of a gene;

Signals: mean scaled fluorescence intensity of brain, heart, kidney, lung, and spleen;

The error bars were based on standard deviation.

OSI: Organ specific index (see text for detail)

Contents

Organ	Page
Brain	2
Heart	12
Kidney	17
Liver	25
Lung	39
Spleen	46

GeneID	GeneName	Signals	Brain	Heart	Kidney	Liver	Lung	Spleen	OSI
X13804	heavy neurofilament polypeptide 854 aa								1.00
NM_013045	tenascin-r restrictin, janusin, j1-160/180 tnr; neural recognition								1.00
NM_017195	growth accentuating protein 43 gap43; gap-43; growth-associated;								1.00
NM_019133_1	synapsin i; syn1								1.00
NM_017327	guanine nucleotide-binding protein alpha subunit; gtp-binding; ratbpgtpc								1.00
L29545	sex-determining region y protein sry1; sry3; sry2								0.99
NM_019128	alpha-internexin alpha-1.6; internexin, alpha inexa								0.99
NM_019284	chondroitin sulfate proteoglycan 5 neuroglycan c cspg5; caleb isoform								0.99
NM_019175	myelencephalon specific protease; protease, serine, 9 prss9								0.99
NM_012526	chromogranin b, parathyroid secretory protein chgb; b								0.99
NM_012659	somatostatin precursor; pre-prosomatostatin;								0.99
NM_012686	neural visinin-like protein 1 vsnl1; nvp								0.99
NM_012625	melanin-concentrating hormone precursor; melanin concentrating								0.99
NM_017010	glutamate receptor, ionotropic, n-methyl d-aspartate 1; grin1								0.99
mwgrat10K#8445	expression: brain; strains: wistar_kyoto; similar to pir								0.99
mwgrat10K#7042	expression: brain; strains: shrsp sprague_dawley wistar_kyoto;								0.99
mwgrat10K#6932	expression: brain; strains: shrsp sprague_dawley wistar_kyoto;								0.99
mwgrat10K#9270	expression: brain; strains: shrsp; similar to pir nf00518941 shd protein								0.99
mwgrat10K#9229	expression: brain; strains: shrsp; similar to pir nf00536642 tubulin								0.99
mwgrat10K#8470	expression: brain; strains: wistar_kyoto; similar to pir								0.99
U47331	metabotropic glutamate receptor 4b								0.99
NM_031974	clathryn light chain lca1; clathrin clta								0.99
NM_031782	vesicular inhibitory amino acid transporter viaat; gaba vgat								0.99

GeneID	GeneName	Signals	Brain	Heart	Kidney	Liver	Lung	Spleen	OSI
NM_021841	gamma-aminobutyric acid gaba-a receptor, subunit alpha 6 gabra6;	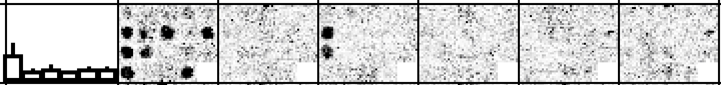							0.99
NM_057201	g protein-coupled receptor 37 endothelin receptor type b-like gpr37;	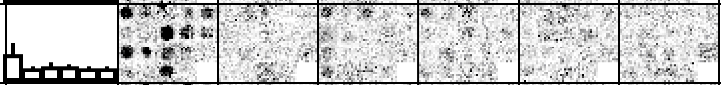							0.99
NM_053859	solute carrier family 17 sodium-dependent inorganic	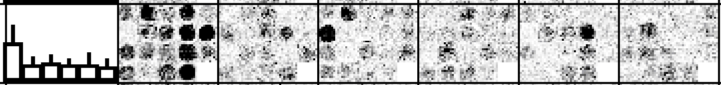							0.99
NM_053856	secretogranin iii scg3	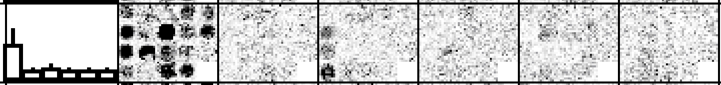							0.99
D17764	phosphoneuroprotein 14	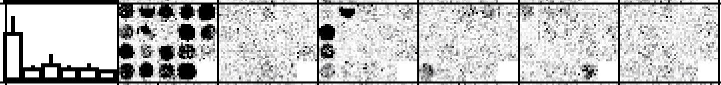							0.99
AF208125	gonadotropin-regulated long chain acyl-coa synthetase; gr-lacs	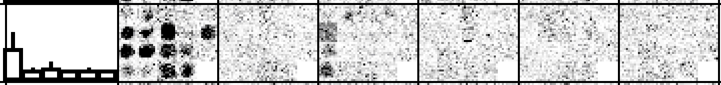							0.99
AY045577	ephrin a3	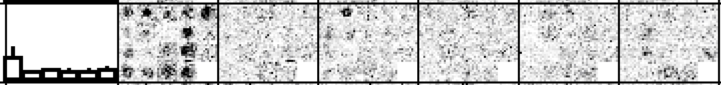							0.99
AB040468	exchange factor for arf6 efa6	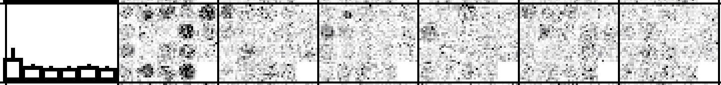							0.99
AF044201	neural membrane protein 35	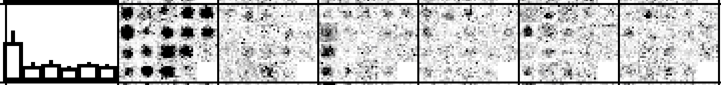							0.99
AF085195	endothelial nitric oxide synthase nosiii	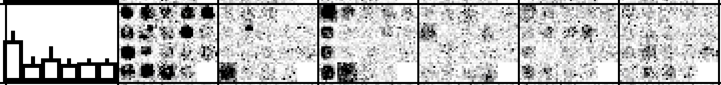							0.99
NM_053511	neural f box protein nfb42 fbxo2	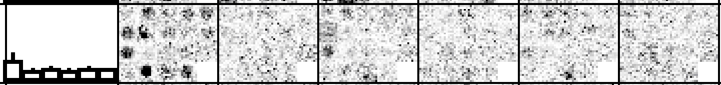							0.98
NM_032083	chimerin chimaerin 1 chn1; n-chimaerin	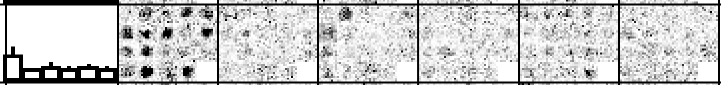							0.98
NM_053835_1	clathrin, light polypeptide lcb cltb; clathryn chain lcb2	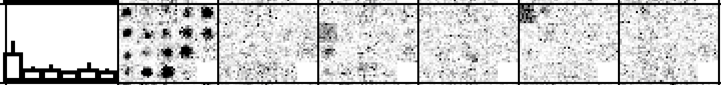							0.98
NM_053779	serine or cysteine proteinase inhibitor, clade i neuroserpin,	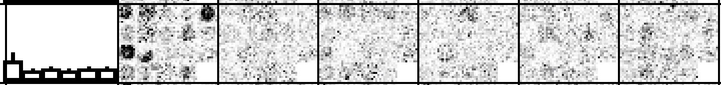							0.98
NM_053543	neurochondrin ncdn-pending; norbin	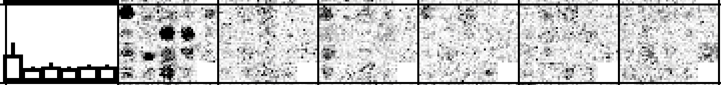							0.98
NM_021595	ninjurin 2 ninj2; ninjurin2	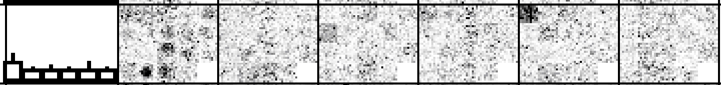							0.98
NM_019161	short type pb-cadherin; cadherin 22 cdh22	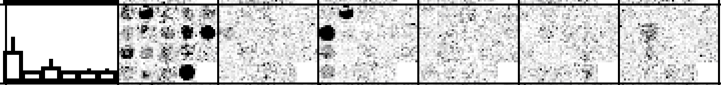							0.98
AF318578	reg i binding protein i; rbp1	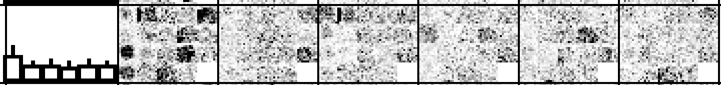							0.98
NM_021758	lin-7-a veli1a	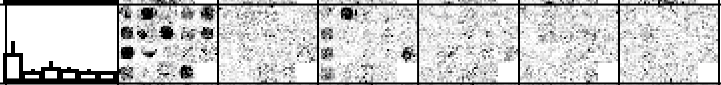							0.98
NM_022668	myelin/oligodendrocyte glycoprotein mog; myelin oligodendrocyte	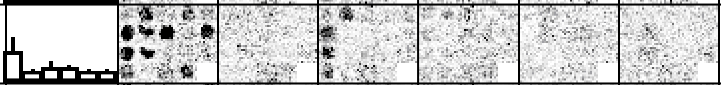							0.98
NM_022499	parvalbumin calcium binding protein pvalb	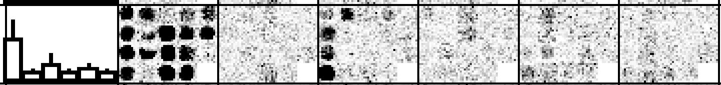							0.98
NM_022008	fxyd domain-containing ion transport regulator 7; fxyd7	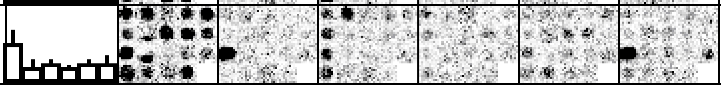							0.98
mwgrat10K#8443	expression: brain; strains: wistar_kyoto; similar to	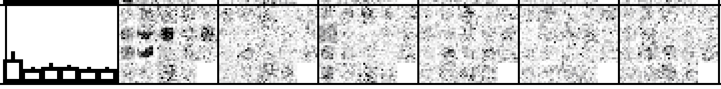							0.98

GeneID	GeneName	Signals	Brain	Heart	Kidney	Liver	Lung	Spleen	OSI
mwgrat10K#8041	expression: brain; strains: shrsp sprague_dawley; similar to	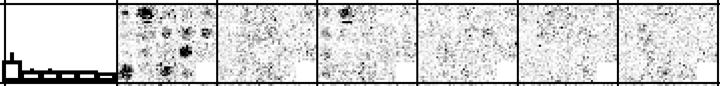							0.98
mwgrat10K#7980	expression: brain; strains: shrsp sprague_dawley; similar to	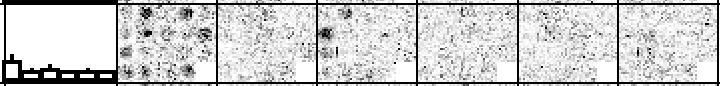							0.98
AB073714	bone morphogenetic protein type ii receptor; bmpr-ii	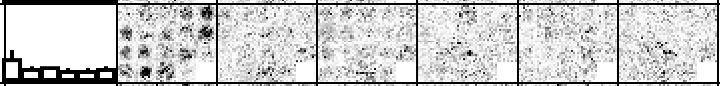							0.98
mwgrat10K#8995	expression: brain; strains: sprague_dawley; similar to	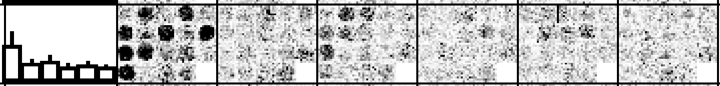							0.98
mwgrat10K#8909	expression: brain; strains: sprague_dawley; similar to pir	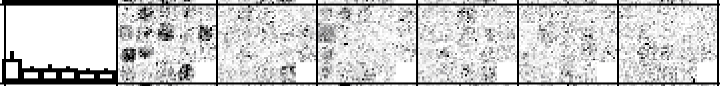							0.98
mwgrat10K#8563	expression: brain; strains: wistar_kyoto; similar to pir	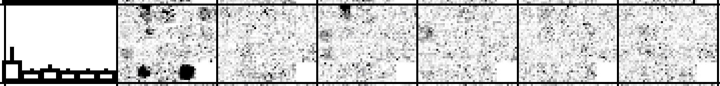							0.98
mwgrat10K#7958	expression: brain; strains: shrsp sprague_dawley; similar to pir	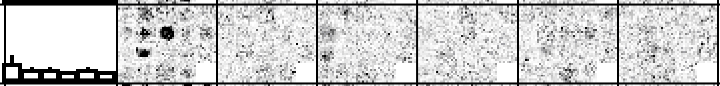							0.98
AF022088	guanine nucleotide binding protein gamma 3 subunit	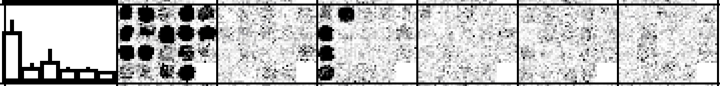							0.98
U78304	w307	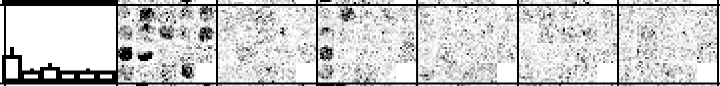							0.98
AF026530_1	stathmin-like-protein splice variant rb3''; rb3; rb3'	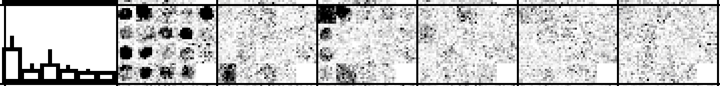							0.98
mwgrat10K#6936	expression: brain; strains: shrsp sprague_dawley wistar_kyoto;	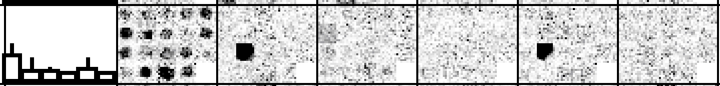							0.98
mwgrat10K#7956	expression: brain; strains: shrsp sprague_dawley; similar to	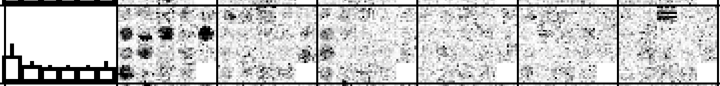							0.98
mwgrat10K#7662	expression: brain; strains: shrsp sprague_dawley wistar_kyoto;	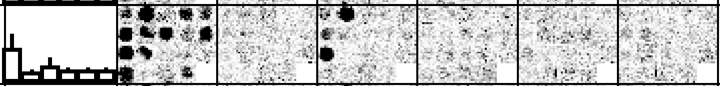							0.98
mwgrat10K#7120	expression: brain; strains: shrsp sprague_dawley wistar_kyoto;	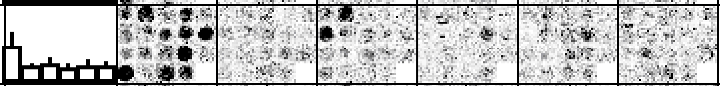							0.98
D37934	5e5 antigen	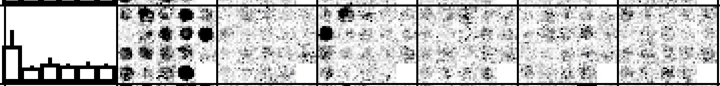							0.98
NM_017190	brain-specific neuronal polypeptide 1b236; protein; myelin-associated	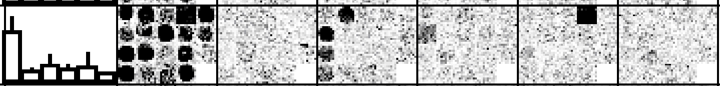							0.98
D10041	phosphatidylinositol 4-kinase phosphatidylinositol 4-kinase, pi	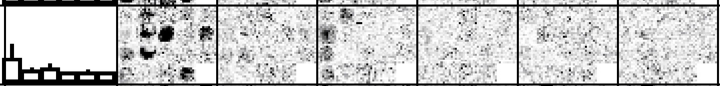							0.98
NM_024140	neurogranin protein kinase c substrate, rc3 nrgn; neurogranin/rc3	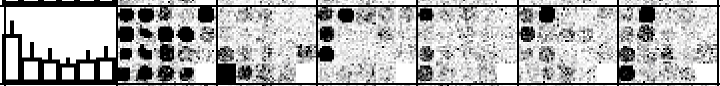							0.97
mwgrat10K#7180	expression: brain; strains: shrsp sprague_dawley wistar_kyoto;	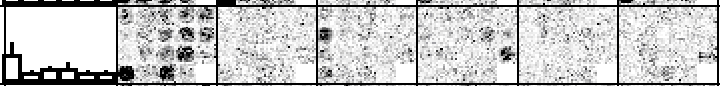							0.97
mwgrat10K#7262	expression: brain; strains: shrsp sprague_dawley wistar_kyoto;	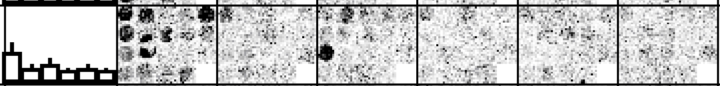							0.97
NM_024128	brain specific mrna b; clone p1a75; bsmrb	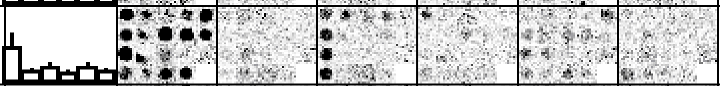							0.97
AJ315761	caldendrin, isoform s1; caldendrin	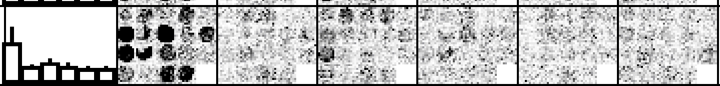							0.97
NM_022669	secretogranin ii precursor aa -30 to 589; scg2	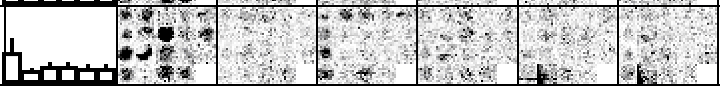							0.97

GeneID	GeneName	Signals	Brain	Heart	Kidney	Liver	Lung	Spleen	OSI
mwgrat10K#7965	expression: brain; strains: shrsp; similar to gbp|x61435|x61435_1								0.97
mwgrat10K#7952	expression: brain; strains: shrsp sprague_dawley; similar to pir								0.97
NM_031028	gamma-aminobutyric acid gaba b receptor, 1 gabbr1; gabab receptor								0.97
Y09000	dendrin								0.97
U11418	nmdar1 glutamate receptor subunit								0.97
X67241	p140 ras-grf								0.97
X89638	myelin-associated/oligodendrocytic basic protein; mobp-99								0.97
mwgrat10K#6632	expression: brain; strains: sprague_dawley wistar_kyoto;								0.97
NM_013002	neuron specific protein pep-19 purkinje cell protein 4 pcp4;								0.97
mwgrat10K#7017	expression: brain; strains: shrsp sprague_dawley wistar_kyoto;								0.97
NM_013414	precursor polypeptide; osteocalin; bgp; osteocalcin								0.97
NM_053441	blood-brain barrier specific anion transporter loc84511; bsat1								0.97
mwgrat10K#9707	expression: heart; strains: shrsp; mwg own new gene sequence								0.97
NM_021748	n-ethylmaleimide sensitive factor erg1; nsf								0.97
mwgrat10K#8996	expression: brain; strains: sprague_dawley; similar to pir								0.97
mwgrat10K#8498	expression: brain; strains: wistar_kyoto; similar to pir								0.97
AB051807	per1 interacting protein of the suprachiamatic nucleus; pips								0.97
mwgrat10K#7067	expression: brain; strains: shrsp sprague_dawley wistar_kyoto;								0.96
mwgrat10K#8509	expression: brain; strains: wistar_kyoto; similar to pir								0.96
mwgrat10K#7974	expression: kidney brain; strains: shrsp; similar to								0.96
NM_024346	scgn10 like-protein sclip; scg10-like-protein								0.96
NM_022217	amphiphysin amph1								0.96
AJ132897	myelin basic protein mbp								0.96

GeneID	GeneName	Signals	Brain	Heart	Kidney	Liver	Lung	Spleen	OSI
mwgrat10K# 6907	expression: liver brain; strains: sprague_dawley wistar_kyoto;								0.96
U18771	rab26								0.96
mwgrat10K# 6886	expression: brain; strains: shrsp sprague_dawley wistar_kyoto;								0.96
AF110797_ 2	gabab receptor subtype 1c form a gababr1; 1a; 1b; b; gaba type 1f;								0.96
AF101041	snrpn upstream reading frame protein snurf								0.96
AF196201	t-cell receptor v delta 2								0.96
NM_080482	deleted in bladder cancer chromosome region candidate 1;								0.96
mwgrat10K# 7967	expression: brain; strains: shrsp; similar to gbp|ay027918|ay027918_1								0.96
X52817	c1-13 gene product aa 1-267								0.96
mwgrat10K# 8285	expression: brain; strains: shrsp sprague_dawley wistar_kyoto; mwg								0.96
D86556	protein kinase								0.95
AF322216	inhibin binding protein long isoform								0.95
NM_032061	contactin associated protein 1 cntnap1; paranodin; caspr								0.95
NM_057190 _2	nasal embryonic lhrh factor nelf; jacob protein jac								0.95
NM_016992	arginine vasopressin diabetes insipidus, same as di conflicting								0.95
NM_013128	carboxypeptidase h precursor ec 3.4.17.10; e cpe; aa 1-476								0.95
NM_080478	amyloid beta a4 precursor protein-binding, family b, member 1								0.95
mwgrat10K# 7234	expression: liver brain; strains: wistar_kyoto; similar to								0.95
mwgrat10K# 8318	expression: kidney brain; strains: shrsp wistar_kyoto; mwg own new								0.95
mwgrat10K# 6394	expression: brain; strains: shrsp sprague_dawley wistar_kyoto;								0.95
mwgrat10K# 7097	expression: brain; strains: sprague_dawley wistar_kyoto;								0.95
mwgrat10K# 9321	expression: brain; strains: shrsp; similar to gbp|bc008881|bc008881_1								0.94
mwgrat10K# 6368	expression: brain; strains: sprague_dawley wistar_kyoto;								0.94

GeneID	GeneName	Signals	Brain	Heart	Kidney	Liver	Lung	Spleen	OSI
NM_053613	reticulon 4 receptor rtn4r; nogo	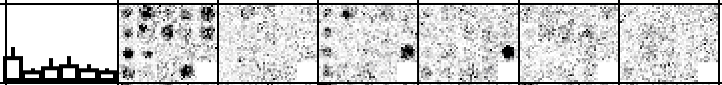							0.94
NM_053457	claudin-11 cldn11	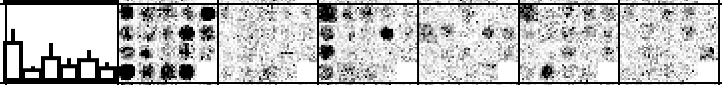							0.94
NM_013038	syntaxin binding protein 1 stxbp1; munc18-1; n-sec1; sec1 homolog	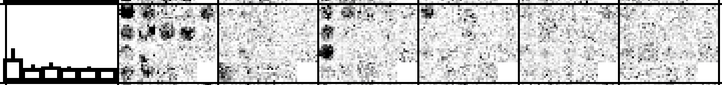							0.94
NM_022249	etoile, sam68-like protein slm-2 etle	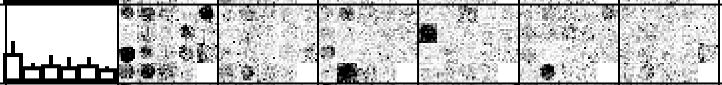							0.94
NM_030991	synaptosomal-associated protein, 25 kda snap25; snap-25a; snap-25b;	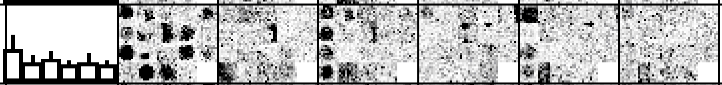							0.94
NM_031783_1	light molecular-weight neurofilament nf-l; neurofilament, polypeptide nfl	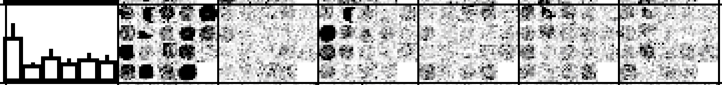							0.94
mwgrat10K#9705	expression: brain; strains: shrsp; mwg own new gene sequence	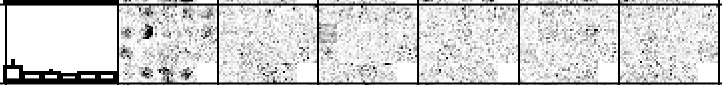							0.94
NM_021859	non-receptor protein kinase protein batk	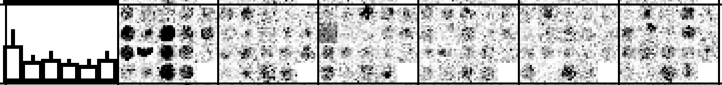							0.94
AB011529	megf3	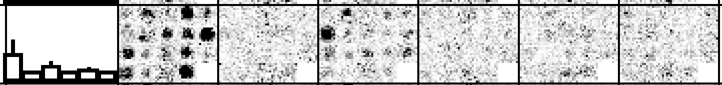							0.93
AJ314857_1	lysosomal atpase; atp6g	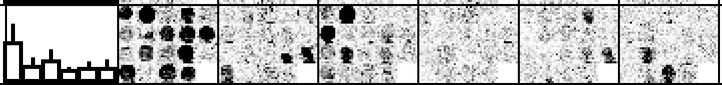							0.93
NM_012507	atpase, na transporting, beta polypeptide 2 atp1b2; na, k	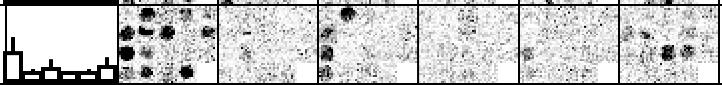							0.93
mwgrat10K#6873	expression: kidney heart brain; strains: shrsp sprague_dawley	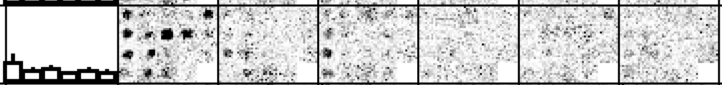							0.93
NM_031630	dead aspartate-glutamate-alanine-aspartat	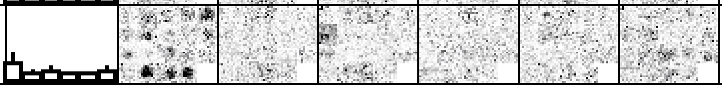							0.93
NM_053573	neuronal olfactomedin-related er localized protein d2sut1e;	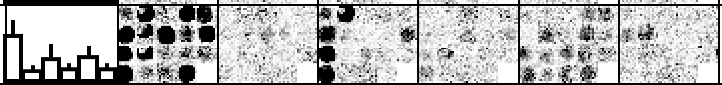							0.93
mwgrat10K#9251	expression: brain; strains: shrsp; similar to gbp|ak005398|ak005398_1	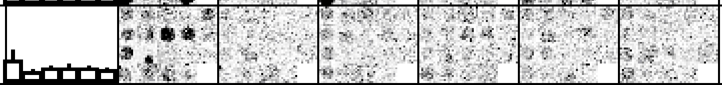							0.93
U78517	camp-regulated guanine nucleotide exchange factor ii camp-gefii	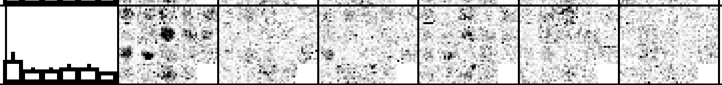							0.93
NM_024163	brain-enriched guanylate kinase-associated protein 1; 2;	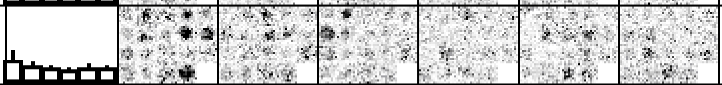							0.93
U86635	glutathione s-transferase m5	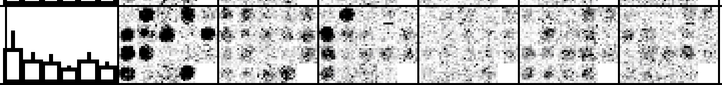							0.92
NM_012505	na and k atpase, alpha catalytic subunit precursor; na transporting,	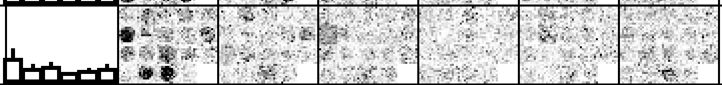							0.92
mwgrat10K#7020	expression: heart brain; strains: shrsp sprague_dawley wistar_kyoto;	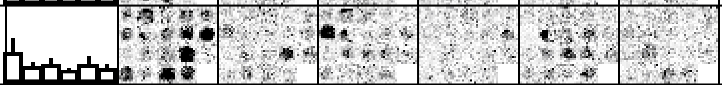							0.92
mwgrat10K#8025	expression: brain; strains: shrsp; similar to gbp|d29951|d29951_1	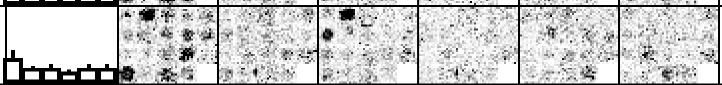							0.92
NM_012506	atpase, na transporting, alpha 3 subunit atp1a3; na,k+atpase	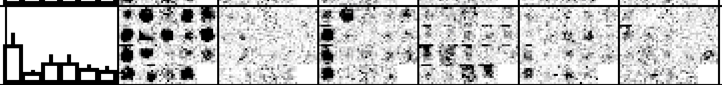							0.92
mwgrat10K#6423	expression: brain; strains: wistar_kyoto; similar to	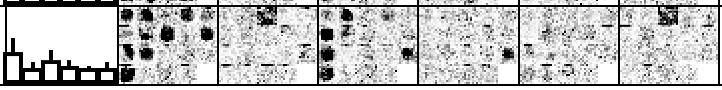							0.92

GeneID	GeneName	Signals	Brain	Heart	Kidney	Liver	Lung	Spleen	OSI
NM_057196	brain-specific angiogenesis inhibitor 1-associated protein 2; baiap2	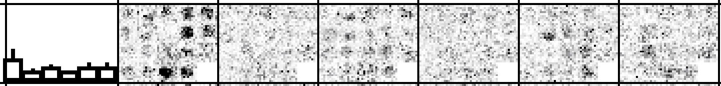							0.92
NM_032060	complement component 3a receptor 1 c3ar1; anaphylatoxin c3a	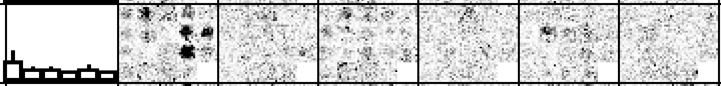							0.92
mwgrat10K#7786	expression: brain; strains: sprague_dawley; similar to pir	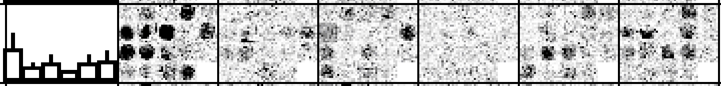							0.91
mwgrat10K#6578	expression: brain heart; strains: shrsp sprague_dawley; similar to	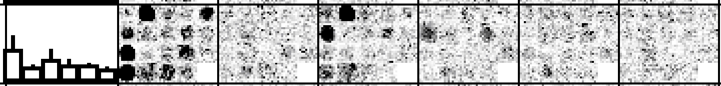							0.91
mwgrat10K#7775	expression: brain; strains: sprague_dawley; similar to	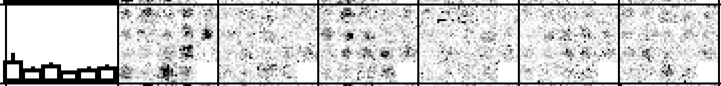							0.91
NM_017139	preproenkephalin 2 penk2; enkephalin enk; proenkephalin	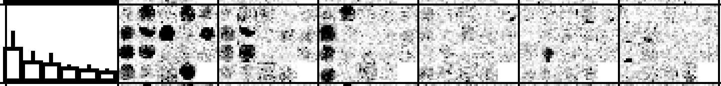							0.91
NM_053440	superiorcervical ganglia, neural specific 10 scgn10; scg10	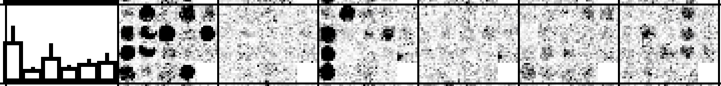							0.91
NM_017204	microtubule-associated protein 6 mtap6; stop	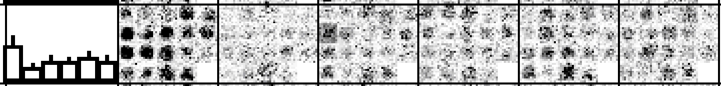							0.91
NM_019225_1	glutamate transporter glutamate transporter, glut-1; glast-1a; solute	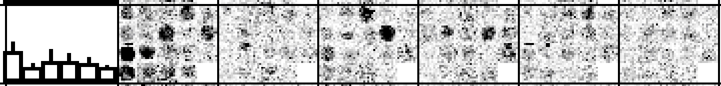							0.91
U49058	ra4	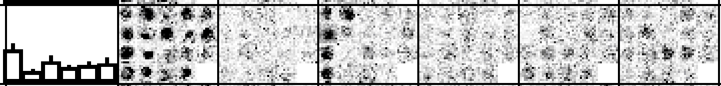							0.91
mwgrat10K#6882	expression: liver heart brain; strains: shrsp sprague_dawley wistar_kyoto;	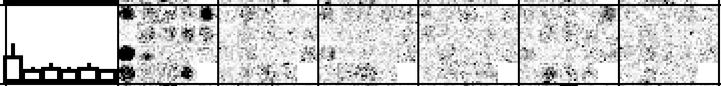							0.90
NM_053722	clip-associating protein 2 clasp2	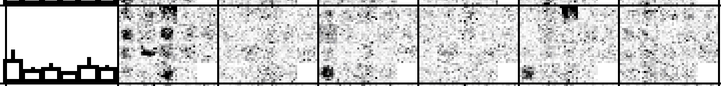							0.90
NM_017042	calcineurin subunit a beta ppp3cb; a-beta	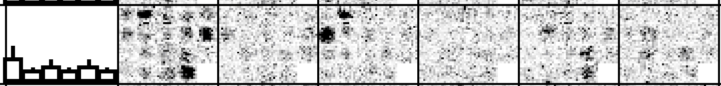							0.90
AF205635	cell division cycle 42 cdc42	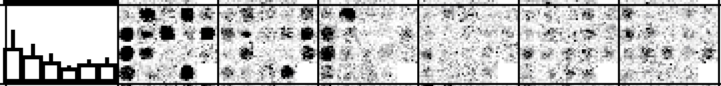							0.90
NM_017252	pou domain, class 3, transcription factor 4 pou3f4; rhs2 iii protein;	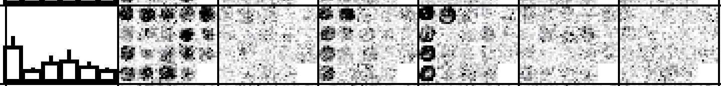							0.90
NM_021853	potassium channel subunit slack slack	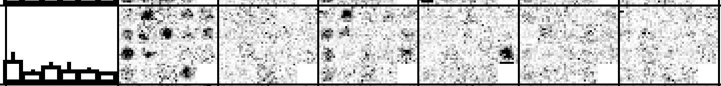							0.89
NM_017262	glutamate receptor, ionotropic, kainate 5 grik5; receptor ka2 subunit	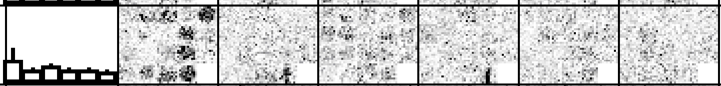							0.89
AF001953	g protein beta 5 subunit	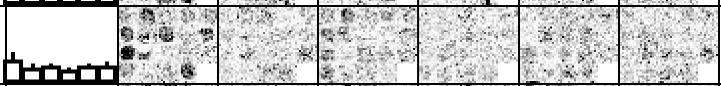							0.89
NM_022864	complexin i cplx1; synaphin 2	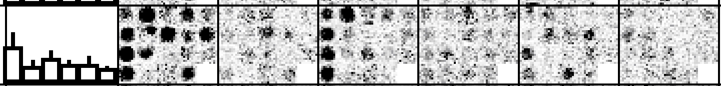							0.89
mwgrat10K#7233	expression: brain; strains: shrsp sprague_dawley wistar_kyoto;	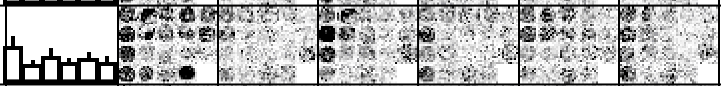							0.89
NM_017155	adenosine a1 receptor adora1; 326 as	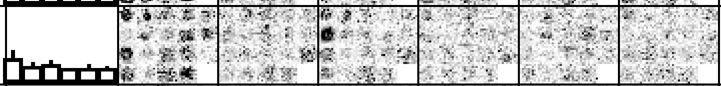							0.89
M17784_1	protease nexin pn-1; gdn precursor	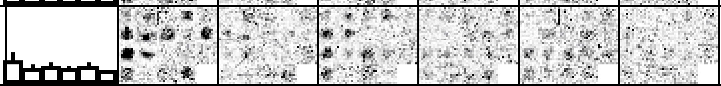							0.88
mwgrat10K#6622	expression: kidney heart; strains: shrsp wistar_kyoto; similar to pir	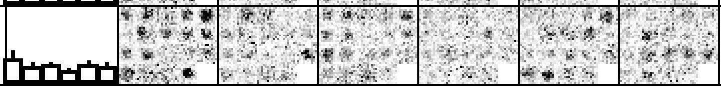							0.88

GeneID	GeneName	Signals	Brain	Heart	Kidney	Liver	Lung	Spleen	OSI
NM_013122	insulin-like growth factor binding protein precursor; insulin	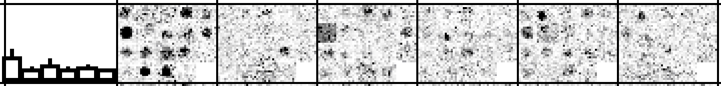							0.88
mwgrat10K#7773	expression: brain; strains: shrsp sprague_dawley; similar to	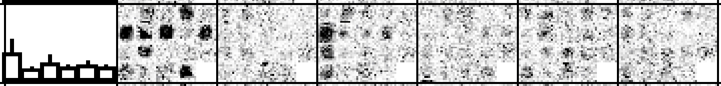							0.88
NM_022865	gephyrin geph	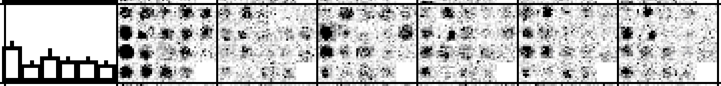							0.88
NM_030873	profilin iia pfn2; ii	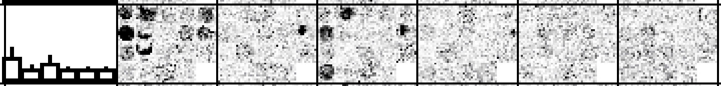							0.88
mwgrat10K#9370	expression: brain; strains: shrsp; similar to pir nf00516024 probable	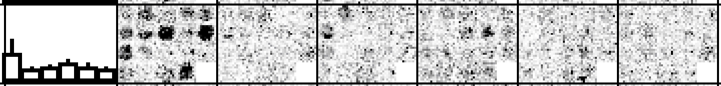							0.88
NM_054003	beta-1,3-glucuronyltransferase 1 glucuronosyltransferase p b3gat1;	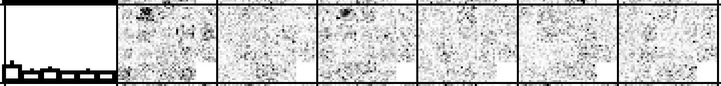							0.88
AF288611	guanylate-cyclase regulatory protein gcrp	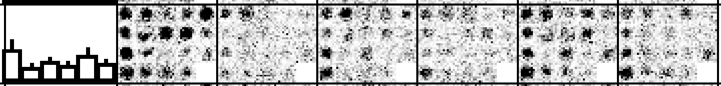							0.87
mwgrat10K#8955	expression: brain; strains: sprague_dawley; similar to	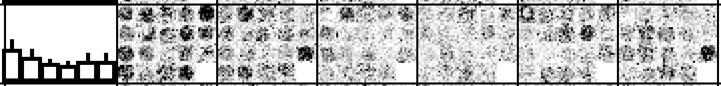							0.87
AJ271834	protein phosphatase 1b2 53 kda isoform ppm1b2	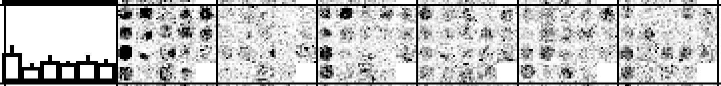							0.87
NM_017009	glial fibrillary acidic protein alpha gfap	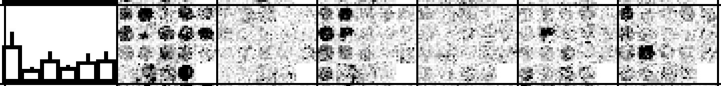							0.87
NM_053931	peanut drosophila -like 1 pnutl1; cdcrel-1a cdcrel-1/pnutl1	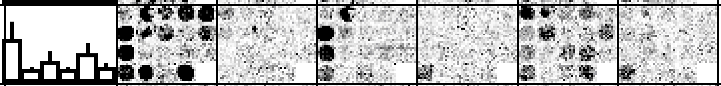							0.87
NM_017029	neurofilament protein, middle polypeptide nefm; nf-m protein	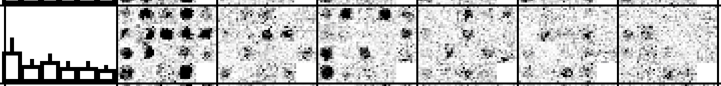							0.87
L25387	phosphofructokinase c pfk-c	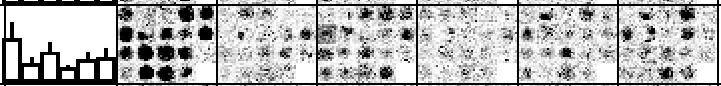							0.86
NM_022209	brbeta b-regulatory subunit of protein phosphatase 2a loc60660	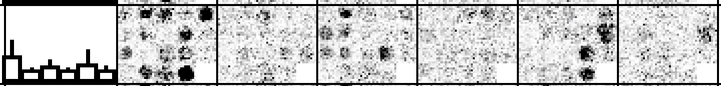							0.86
mwgrat10K#9284	expression: brain; strains: shrsp; similar to pir nf00514283 rho-related	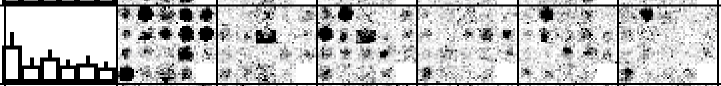							0.86
NM_023960	calcium activated potassium channel beta 4 subunit kcnmb4;	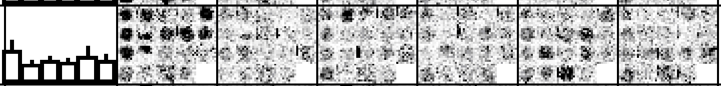							0.86
AF348446	fibroblast growth factor homologous factor 1b; fhf1b	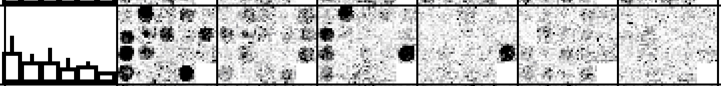							0.86
NM_031728	synaptosomal-associated protein, 91 kda snap91; assembly protein 180	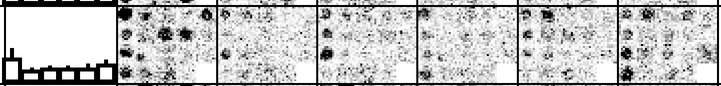							0.86
NM_013191	s100 calcium-binding protein, beta neural s100b; protein; s-100	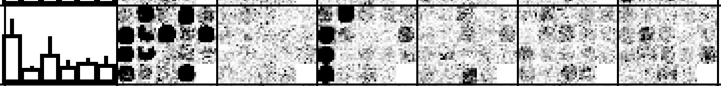							0.86
AF009603	sh3p4	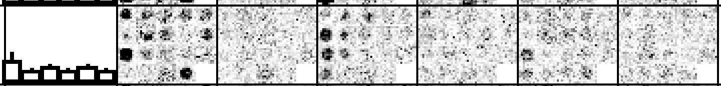							0.86
X59737	ubiquitous mitochondrial creatine kinase	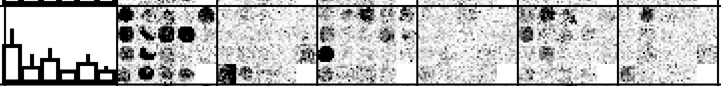							0.86
mwgrat10K#9223	expression: brain; strains: shrsp; similar to pir nf00531703 cyfip2 -	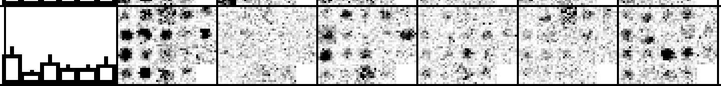							0.85
NM_017237	ubiquitin carboxy-terminal hydrolase l1 uchl1; carboxyl-terminal pgp9.5	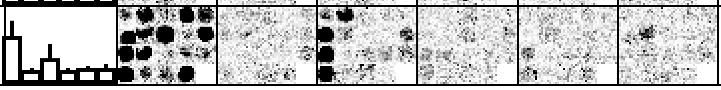							0.85

GeneID	GeneName	Signals	Brain	Heart	Kidney	Liver	Lung	Spleen	OSI
NM_017122	neuron specific calcium-binding protein p23k molecule l1; hippocalcin								0.84
NM_019278	regulated endocrine-specific protein 18 resp18; neuroendocrine-specific								0.84
NM_019350	synaptotagmin 5 syt5; v								0.84
NM_017197	apoptosis-related rna binding protein napor-3; etr-r3a etr-r3; etr-r3b;								0.84
mwgrat10K#8532	expression: brain; strains: wistar_kyoto; similar to								0.83
NM_017253	branched chain aminotransferase 1, cytosolic bcat1; branch bcatc								0.83
NM_030990	proteolipid protein; pelizaeus-merzbacher disease,								0.83
mwgrat10K#9226	expression: brain; strains: shrsp; similar to pir nf00520191 rap2								0.83
mwgrat10K#8943	expression: brain; strains: sprague_dawley; similar to pir								0.83
NM_013175	secretory granule neuroendocrine, protein 1 7b2 protein sgne1;								0.82
NM_053878	complexin 2 cplx2; synaphin 1; ii								0.82
mwgrat10K#6950	expression: brain; strains: shrsp sprague_dawley wistar_kyoto;								0.82
NM_031066	protein kinase c-binding protein zeta1 fez1; zygini								0.81
NM_022695	ntr2 receptor ntsr2								0.81
NM_024141	nadh/nadph thyroid oxidase thox2 thox2								0.81
mwgrat10K#9031	expression: brain; strains: sprague_dawley; similar to pir								0.80
U17603	rs-rex-s								0.80
mwgrat10K#8283	expression: heart brain; strains: shrsp sprague_dawley wistar_kyoto;								0.80
NM_012829	cck precursor aa -28 to 87; preprocholecystokinin;								0.80
NM_031024_1	drebrin a dbn1; e								0.80
mwgrat10K#9267	expression: brain; strains: shrsp; similar to pir nf00989417 unnamed								0.80
AF141863	mt2 melatonin receptor mel-1b								0.80
NM_031676	neuronal protein np25-pending; np25								0.79

GeneID	GeneName	Signals	Brain	Heart	Kidney	Liver	Lung	Spleen	OSI
mwgrat10K#8462	expression: brain; strains: wistar_kyoto; similar to pir	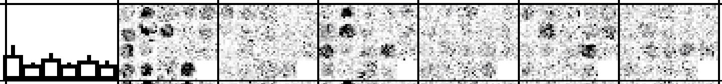							0.79
X79881	aggrecan like protein/ brevican; core protein	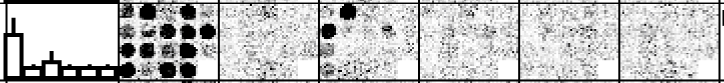							0.77
mwgrat10K#9307	expression: brain; strains: sprague_dawley; similar to	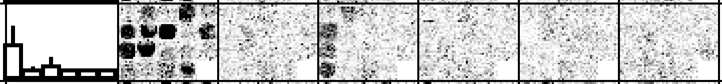							0.76
NM_017026	myelin basic protein mbp	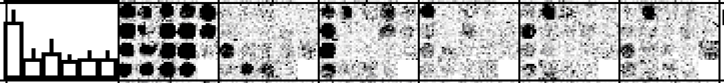							0.76
NM_031641	sulfotransferase-related protein sultx3-pending; nervous system	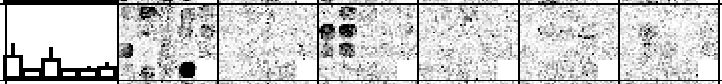							0.76
mwgrat10K#8550	expression: brain; strains: wistar_kyoto; similar to pir	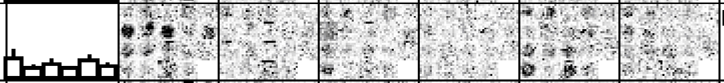							0.76
NM_012777	apolipoprotein d apo d; apod	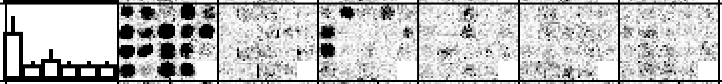							0.75
mwgrat10K#8481	expression: brain; strains: wistar_kyoto; similar to pir	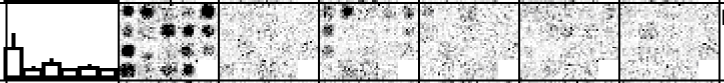							0.75
mwgrat10K#8286	expression: liver brain; strains: sprague_dawley wistar_kyoto; mwg	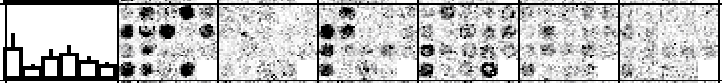							0.75
Y17048	caldendrin	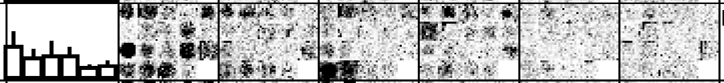							0.75
NM_017110	cocaine and amphetamine regulated transcript cart; protein	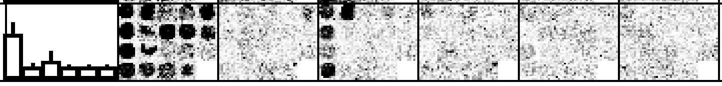							0.74

GeneID	GeneName	Signals	Brain	Heart	Kidney	Liver	Lung	Spleen	OSI
mwgrat10K# 8196	expression: heart; strains: shrsp wistar_kyoto; similar to pir								1.00
mwgrat10K# 6163	expression: heart; strains: sprague_dawley wistar_kyoto;								1.00
NM_017034	pim-1 oncogene pim1								1.00
X60658	potential ligand-binding protein rya3								1.00
mwgrat10K# 6331	expression: heart; strains: shrsp sprague_dawley wistar_kyoto;								0.99
mwgrat10K# 6169	expression: heart; strains: shrsp sprague_dawley wistar_kyoto;								0.99
NM_031677	four and a half lim domains 2 fhl2; dral								0.99
NM_031545	atrial natriuretic peptide iso-anp; bnp; factor; brain nppb								0.99
NM_080399	smhs1 protein; smhs1								0.99
NM_053880 _1	dynein, cytoplasmic, intermediate polypeptide 2 dnci2; cytoplasmic								0.99
mwgrat10K# 9534	expression: heart; strains: shrsp; similar to pir nf00523295								0.99
mwgrat10K# 9489	expression: heart; strains: shrsp; similar to gbp|af237628|af237628_1								0.99
mwgrat10K# 9713	expression: heart; strains: shrsp; mwg own new gene sequence								0.99
mwgrat10K# 9709	expression: heart; strains: shrsp; mwg own new gene sequence								0.99
mwgrat10K# 9124	expression: heart; strains: sprague_dawley; similar to pir								0.99
mwgrat10K# 6700	expression: heart brain; strains: shrsp sprague_dawley wistar_kyoto;								0.99
mwgrat10K# 6463	expression: heart; strains: shrsp sprague_dawley wistar_kyoto;								0.99
mwgrat10K# 8569	expression: heart; strains: sprague_dawley; similar to								0.99
mwgrat10K# 8075	expression: heart; strains: shrsp; similar to gbp|ak019393|ak019393_1								0.99
mwgrat10K# 6638	expression: kidney heart brain; strains: shrsp sprague_dawley								0.98
NM_053826	pyruvate dehydrogenase kinase, isoenzyme 1 pdk1; kinase pdh								0.98
S74398	l-type ca2 channel alpha 1 subunit								0.98
NM_021590	aryl-hydrocarbon interacting protein-like 1 aipl1								0.98

GeneID	GeneName	Signals	Brain	Heart	Kidney	Liver	Lung	Spleen	OSI
NM_013200_1	carnitine palmitoyltransferase 1 beta, muscle isoform cpt1b; i cpti; like								0.98
NM_053395	small muscle protein, x-linked; smpx								0.98
AJ243193	cardiovascular heat shock protein; cvhsp								0.98
AJ001290	sodium myo-inositol transporter								0.98
mwgrat10K#6323	expression: heart; strains: sprague_dawley wistar_kyoto;								0.97
M59211	k channel protein kshiiia3; potassium kv3.2b								0.97
X07314	cardiac myosin light chain 2; mlc2 aa 1-166								0.97
mwgrat10K#6375	expression: heart; strains: sprague_dawley wistar_kyoto;								0.97
AJ291433	mono; adp-ribosyltransferase; art3								0.97
NM_017263	glutamate receptor subunit 4c glutamate receptor subunit 4c,								0.97
mwgrat10K#8013	expression: kidney; strains: shrsp; similar to gbp|ak006287|ak006287_1								0.97
NM_013021	peripherin retinal degradation slow rds; gene producct aa 1-346								0.97
mwgrat10K#6379	expression: heart; strains: shrsp sprague_dawley wistar_kyoto;								0.96
AB049626	h-caldesmon								0.96
AF159245	cytochrome p450 cyp2b21								0.96
mwgrat10K#6585	expression: heart; strains: wistar_kyoto; similar to								0.96
mwgrat10K#6860	expression: liver heart; strains: shrsp sprague_dawley; similar to pir								0.96
mwgrat10K#6431	expression: heart; strains: shrsp sprague_dawley; similar to pir								0.96
mwgrat10K#8103	expression: kidney; strains: shrsp; similar to gbp|ak010263|ak010263_1								0.96
mwgrat10K#6399	expression: heart; strains: sprague_dawley wistar_kyoto;								0.96
NM_022382	myomegalin loc64183								0.96
NM_012590	inhibin alpha-subunit precursor; inhibin, alpha inha								0.95
mwgrat10K#6512	expression: heart; strains: shrsp sprague_dawley; similar to								0.95

GeneID	GeneName	Signals	Brain	Heart	Kidney	Liver	Lung	Spleen	OSI
AY059414	concentrative na+nucleoside cotransporter protein; cnt3								0.94
mwgrat10K# 8242	expression: heart; strains: shrsp sprague_dawley wistar_kyoto;								0.94
L47281	alpha-3 type iv collagen col4a3								0.94
L20998	t cell receptor								0.94
mwgrat10K# 8571	expression: heart; strains: wistar_kyoto; similar to pir								0.94
mwgrat10K# 9501	expression: heart; strains: shrsp; similar to pir nf00525260 tescalcin								0.94
mwgrat10K# 8723	expression: liver; strains: wistar_kyoto; similar to								0.94
mwgrat10K# 6231	expression: brain; strains: shrsp wistar_kyoto; similar to								0.93
mwgrat10K# 9110	expression: heart; strains: sprague_dawley; similar to								0.93
mwgrat10K# 8239	expression: kidney heart; strains: shrsp sprague_dawley wistar_kyoto;								0.93
L41684	orf								0.92
M18135_1	minor striated-muscle alpha tropomyosin; isoform; non-muscle;								0.92
mwgrat10K# 6185	expression: heart; strains: shrsp sprague_dawley wistar_kyoto;								0.92
AF053360	nonmuscle tropomyosin 5 tpm5								0.91
mwgrat10K# 6722	expression: kidney heart; strains: shrsp sprague_dawley wistar_kyoto;								0.90
NM_019168	arginase type ii arg2								0.90
NM_017240	myosin heavy chain, cardiac muscle, fetal myh7; chain 5; beta aa 1-1935								0.89
X59736	sarcomeric mitochondrial creatine kinase								0.89
NM_013220	cardiac ankyrin repeat protein alrp; carp								0.89
mwgrat10K# 6494	expression: liver heart; strains: shrsp sprague_dawley wistar_kyoto;								0.89
mwgrat10K# 8477	expression: brain; strains: wistar_kyoto; similar to								0.89
mwgrat10K# 9510	expression: heart; strains: shrsp; similar to gbp|af248643|af248643_1								0.88
NM_012629	prolactin; prl								0.88

GeneID	GeneName	Signals	Brain	Heart	Kidney	Liver	Lung	Spleen	OSI
mwgrat10K#9490	expression: heart; strains: shrsp; similar to gbp|af248643|af248643_1	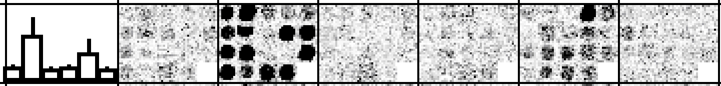							0.88
NM_012949	muscle specific enolase beta beta enolose two splicing products eno3;	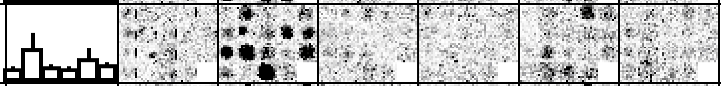							0.88
X03032	phosphorylase aa 760-840; glycogen	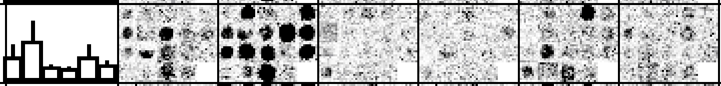							0.87
mwgrat10K#9504	expression: heart; strains: shrsp; similar to gbp|m29793|m29793_1	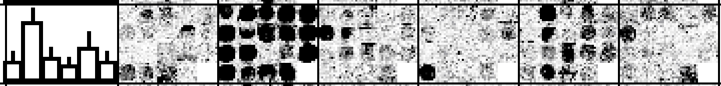							0.87
NM_057144	cysteine-rich protein 3 csrp3; muscle lim mlp	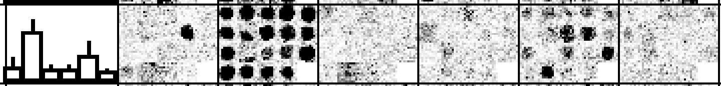							0.87
S70803	clone p10.15 product	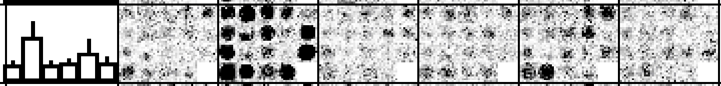							0.87
X74294	alpha 7c integrin alpha 7 integrin	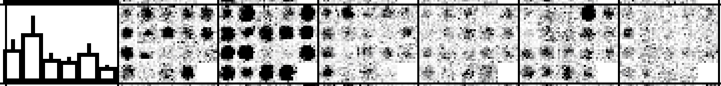							0.86
mwgrat10K#9452	expression: heart; strains: shrsp; similar to gbp|ak005020|ak005020_1	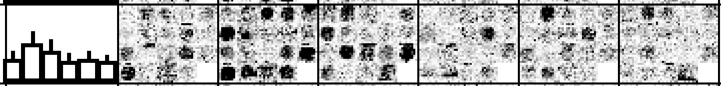							0.86
mwgrat10K#6184	expression: brain heart; strains: sprague_dawley wistar_kyoto;	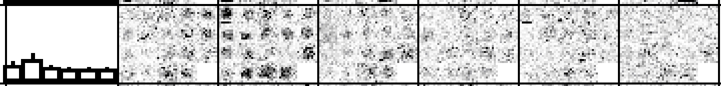							0.86
mwgrat10K#6138	expression: heart brain; strains: shrsp sprague_dawley; similar to pir	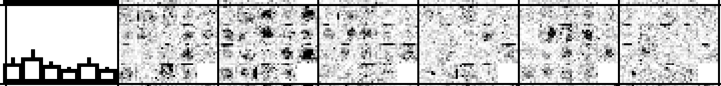							0.84
mwgrat10K#9480	expression: heart; strains: wistar_kyoto; similar to pir	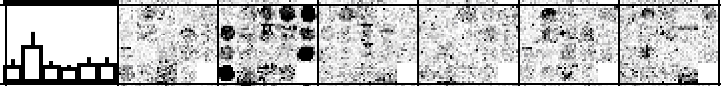							0.83
NM_017131	cardiac calsequestrin; 2 casq2	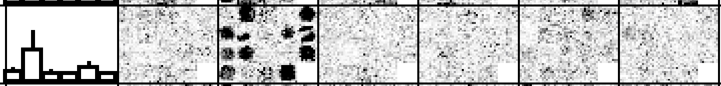							0.82
X78985	cd5 glycoprotein cd5	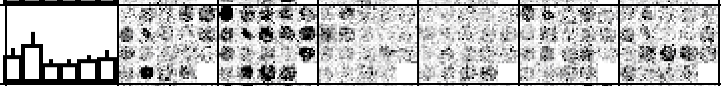							0.82
mwgrat10K#8202	expression: heart; strains: shrsp sprague_dawley wistar_kyoto;	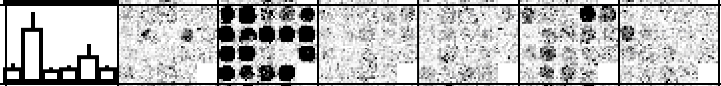							0.82
NM_012676	troponin t, cardiac tnnt2; t	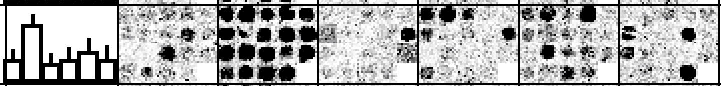							0.81
NM_017241	glutamate receptor, ionotropic, kainate 1 grik1; receptor subunit 5-1	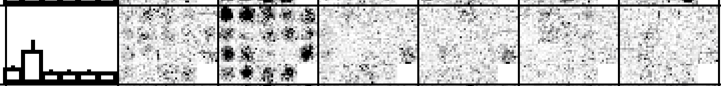							0.80
mwgrat10K#8260	expression: heart; strains: shrsp sprague_dawley wistar_kyoto; mwg	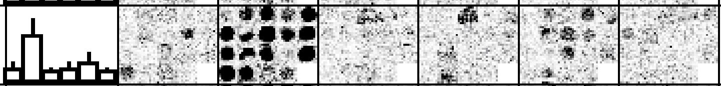							0.80
NM_022707	phospholamban phospholamban, pln; plm	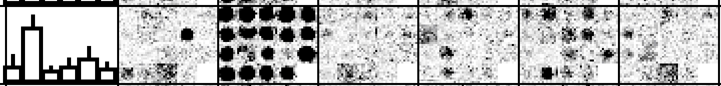							0.79
mwgrat10K#8203	expression: heart; strains: shrsp sprague_dawley wistar_kyoto;	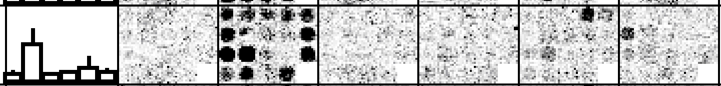							0.79
mwgrat10K#8167	expression: heart; strains: shrsp sprague_dawley wistar_kyoto;	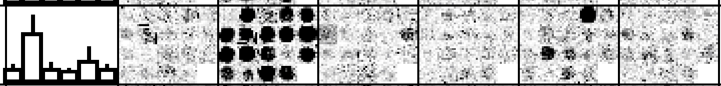							0.78
NM_031056	matrix metalloproteinase 14, membrane-inserted mmp14; mt-mmp	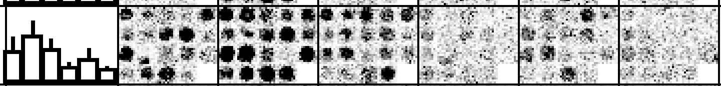							0.77
mwgrat10K#8066	expression: heart kidney; strains: shrsp; similar to pir nf00142326	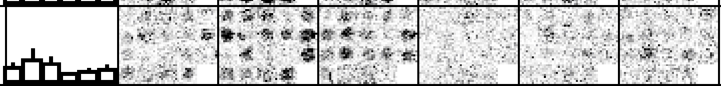							0.76
X65036	integrin alpha chain	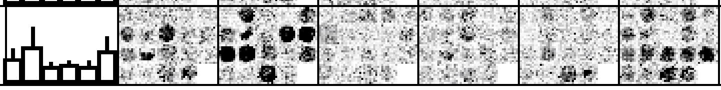							0.76

GeneID	GeneName	Signals	Brain	Heart	Kidney	Liver	Lung	Spleen	OSI
mwgrat10K#8618	expression: heart; strains: wistar_kyoto; similar to	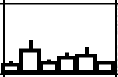	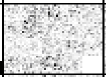	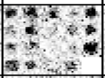	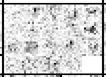	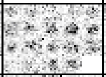	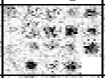	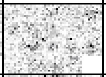	0.74
mwgrat10K#8832	expression: kidney; strains: wistar_kyoto; similar to	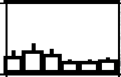	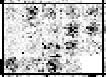	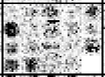	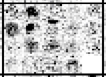	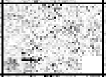	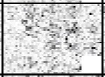	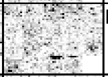	0.71
mwgrat10K#8567	expression: heart; strains: wistar_kyoto; similar to pir	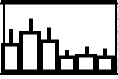	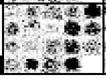	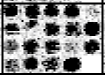	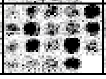	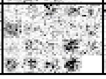	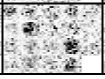	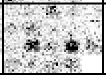	0.69

GeneID	GeneName	Signals	Brain	Heart	Kidney	Liver	Lung	Spleen	OSI
NM_017082	tamm-horsfall protein tamm-horsfall protein, thp; uromodulin; urmodulin	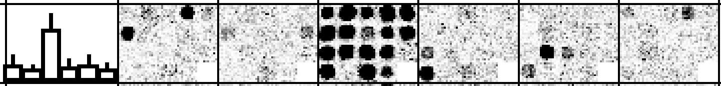							1.00
NM_013183	meprin beta-subunit; 1 beta mep1b	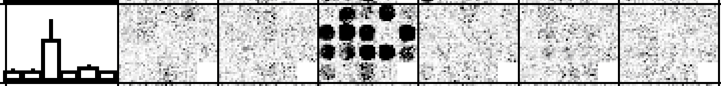							1.00
NM_019345	thiazide-sensitive sodium-chloride cotransporter; solute carrier family	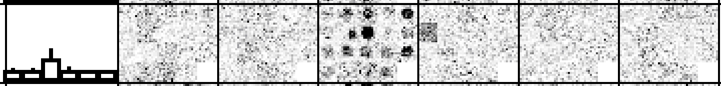							1.00
NM_031044	histamine n-methyltransferase hnmt	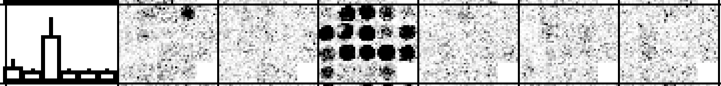							1.00
NM_020976	kidney-specific membrane protein nx-17	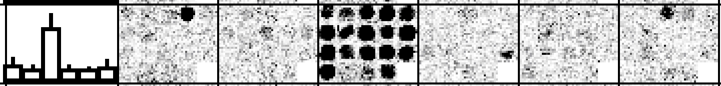							1.00
NM_013097	deoxyribonuclease i variant 1; 2; dnase1	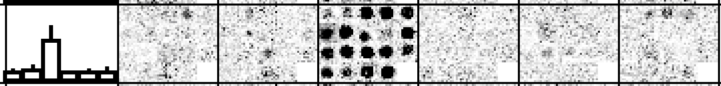							1.00
M32757	inhibin beta-b-subunit precursor	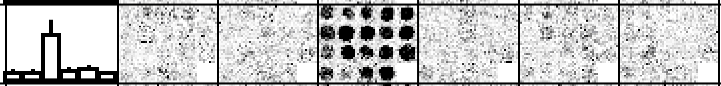							1.00
M26534	kallikrein-like serine protease rskg-50; kallikrein	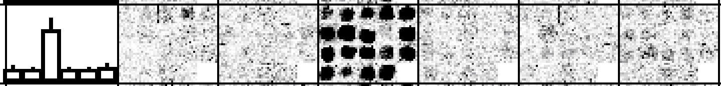							1.00
NM_012842_1	preproepidermal growth factor; epidermal egf	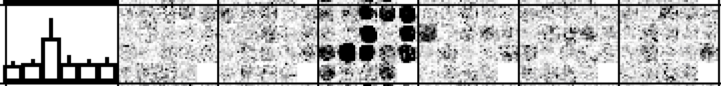							1.00
NM_012909	adh water channel; complete cds; aquaporin 2 aqp2	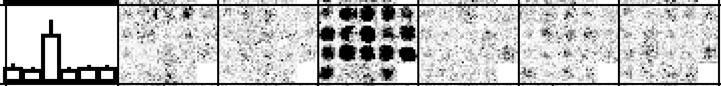							1.00
NM_012888_1	thyroid stimulating hormone receptor precursor; tshr	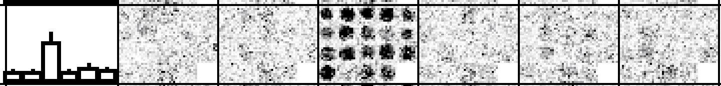							1.00
NM_031584_1	solute carrier family 22, member 2; slc22a2	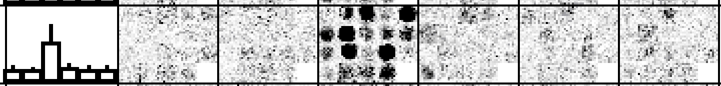							1.00
mwgrat10K#8903	expression: kidney; strains: wistar_kyoto; similar to pir	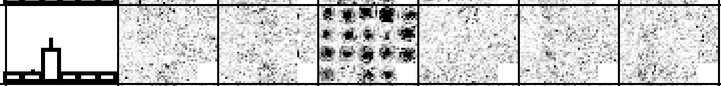							1.00
mwgrat10K#8855	expression: kidney; strains: wistar_kyoto; similar to	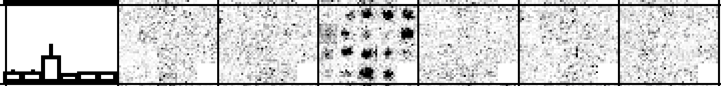							1.00
mwgrat10K#9557	expression: kidney; strains: shrsp; similar to gbp|ak002365|ak002365_1	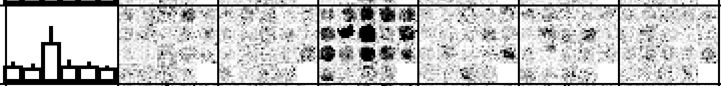							1.00
mwgrat10K#9619	expression: kidney; strains: shrsp; similar to gbp|ak002365|ak002365_1	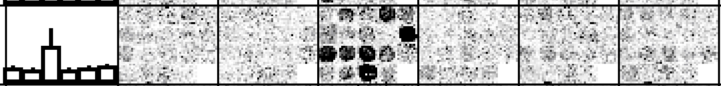							1.00
mwgrat10K#9574	expression: kidney; strains: shrsp; similar to gbp|ax191622|ax191622_1	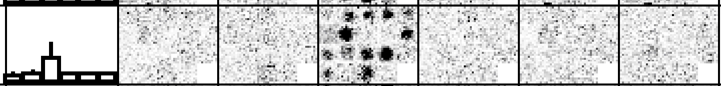							1.00
mwgrat10K#7555	expression: kidney; strains: shrsp wistar_kyoto; similar to pir	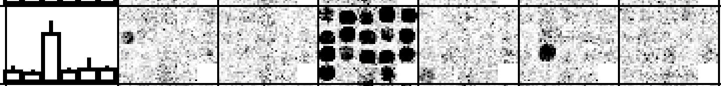							1.00
NM_053929	solute carrier family 7 cationic amino acid transporter, y system, member	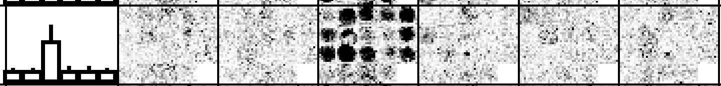							1.00
NM_053840	put. ggt light subunit 380-568 protein sequence is in conflict with the	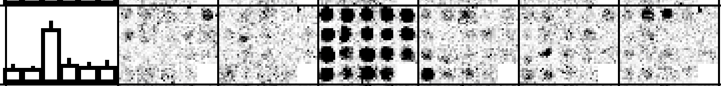							1.00
U51898	ca2+independent phospholipase a2	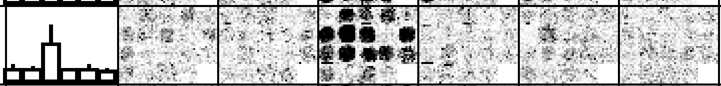							1.00
mwgrat10K#7541	expression: kidney; strains: wistar_kyoto; similar to	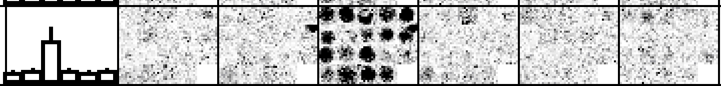							1.00
mwgrat10K#7295	expression: heart; strains: wistar_kyoto; similar to pir	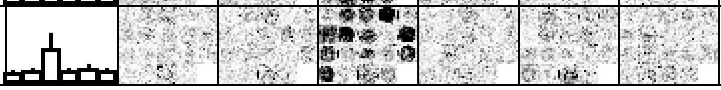							1.00

GeneID	GeneName	Signals	Brain	Heart	Kidney	Liver	Lung	Spleen	OSI
AF187814	putative n-acetyltransferase cml3 cml3								1.00
AF221952	mu-protocadherin								1.00
AB051836	organic anion transporter 5								1.00
M19648	kallikrein kal; t-kininogenase								1.00
AF219904	folate binding protein								0.99
mwgrat10K#7712	expression: liver kidney; strains: shrsp sprague_dawley wistar_kyoto;								0.99
NM_052802	kidney androgen-regulated protein kap								0.99
NM_031982	vanilloid receptor type 1 like protein 1; vr1l1								0.99
NM_031712_1	pdz domain-containing protein; domain containing 1 pdzk1								0.99
NM_022590	low affinity na-dependent glucose transporter sglt2 sglt2;								0.99
mwgrat10K#8904	expression: kidney; strains: wistar_kyoto; similar to								0.99
AF402772	membrane-associated protein map17								0.99
AF374406	epsilon-trimethyllysine 2-oxoglutarate dioxygenase tmlh;								0.99
NM_022388	corticosteroid-induced protein precursor fxyd4; transmembrane 3d								0.99
AF053317	organic anion transporting polypeptide oatp5								0.99
NM_053332	cubilin intrinsic factor-cobalamin receptor cubn; factor-b12 precursor								0.99
mwgrat10K#7321	expression: liver kidney; strains: shrsp wistar_kyoto; similar to								0.99
AF159103	tnf-stimulated gene 6 protein								0.99
mwgrat10K#6992	expression: brain kidney; strains: shrsp wistar_kyoto; similar to								0.99
mwgrat10K#7578	expression: kidney; strains: shrsp wistar_kyoto; similar to								0.99
mwgrat10K#8115	expression: kidney; strains: wistar_kyoto; similar to pir								0.99
AF110025	rst transporter homolog								0.99
mwgrat10K#8623	expression: kidney heart; strains: shrsp sprague_dawley wistar_kyoto;								0.99

GeneID	GeneName	Signals	Brain	Heart	Kidney	Liver	Lung	Spleen	OSI
mwgrat10K#7592	expression: kidney; strains: shrsp wistar_kyoto; similar to								0.99
NM_053626	d-amino acid oxidase dao1; d-amino-acid								0.99
mwgrat10K#8156	expression: liver kidney; strains: shrsp wistar_kyoto; similar to pir								0.99
Y09945	putative integral membrane transport protein ust1r								0.99
U64689	zyginii								0.99
NM_019211	calcium and dag-regulated guanine nucleotide exchange factor ii; ras								0.99
NM_013042	intestinal trefoil factor intestinal trefoil factor, itf; precursor; protein; tff3;								0.99
D26111	chloride channel putative; clc-k2s; clc-k2l								0.99
NM_017216	solute carrier family 3, member 1 slc3a1; l-type neutral amino acid								0.99
mwgrat10K#9635	expression: kidney; strains: shrsp; similar to gbp|bc011540|bc011540_1								0.99
NM_013030	solute carrier family 17 sodium/hydrogen exchanger,								0.98
mwgrat10K#6625	expression: heart kidney brain; strains: shrsp wistar_kyoto; similar								0.98
mwgrat10K#6775	expression: liver kidney; strains: shrsp; similar to pir nf00510497								0.98
AF202049	nm23-r7								0.98
NM_013118	guanylate cyclase activator 2 guanylin guca2a								0.98
mwgrat10K#8305	expression: kidney; strains: shrsp wistar_kyoto; mwg own new gene								0.98
S74029	kynurenine aminotransferase kynurenine aminotransferase,								0.98
mwgrat10K#9721	expression: kidney; strains: shrsp; mwg own new gene sequence								0.98
mwgrat10K#9648	expression: kidney; strains: shrsp; similar to gbp|ak014025|ak014025_1								0.98
mwgrat10K#7593	expression: liver kidney; strains: shrsp sprague_dawley wistar_kyoto;								0.98
mwgrat10K#7728	expression: kidney; strains: shrsp wistar_kyoto; similar to								0.98
mwgrat10K#7680	expression: kidney; strains: shrsp wistar_kyoto; similar to								0.98
J02941	preprorenin ec 3.4.99.19; renin aa 1-702								0.98

GeneID	GeneName	Signals	Brain	Heart	Kidney	Liver	Lung	Spleen	OSI
mwgrat10K# 6926	expression: kidney; strains: shrsp wistar_kyoto; similar to	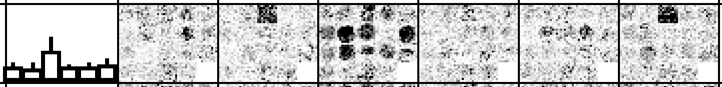							0.98
mwgrat10K# 7172	expression: brain; strains: sprague_dawley wistar_kyoto;	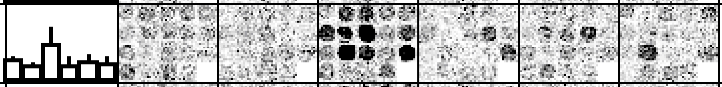							0.98
mwgrat10K# 7559	expression: kidney; strains: shrsp wistar_kyoto; similar to pir	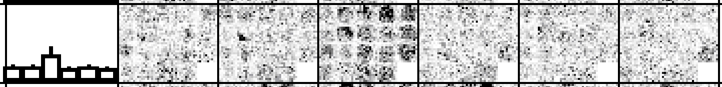							0.98
mwgrat10K# 7320	expression: liver kidney; strains: shrsp sprague_dawley wistar_kyoto;	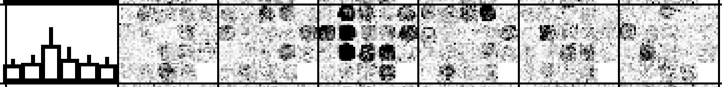							0.98
NM_031746 _1	solute carrier family 13; sodium-dependent dicarboxylate	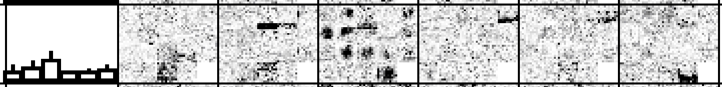							0.98
NM_022224	resiniferatoxin-binding, phosphotriesterase-related protein	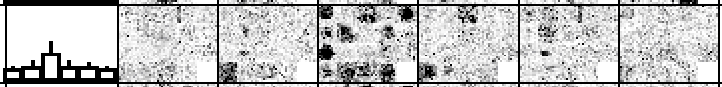							0.98
AF062389	kidney-specific protein ks	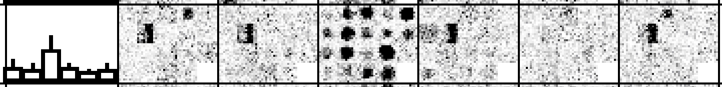							0.98
mwgrat10K# 8835	expression: kidney; strains: wistar_kyoto; similar to	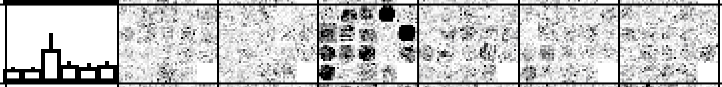							0.98
mwgrat10K# 9157	expression: liver; strains: sprague_dawley; similar to	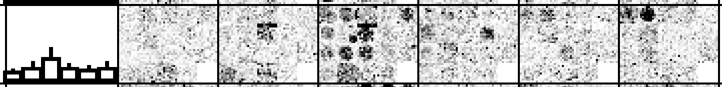							0.97
NM_031523	nerve growth factor, gamma polypeptide ngfg; kallikrein	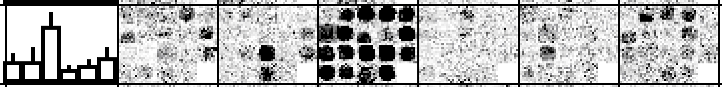							0.97
mwgrat10K# 9637	expression: kidney; strains: shrsp; similar to gbp|ak020131|ak020131_1	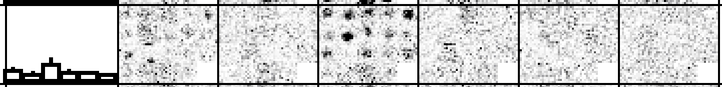							0.97
mwgrat10K# 8144	expression: liver brain; strains: sprague_dawley wistar_kyoto;	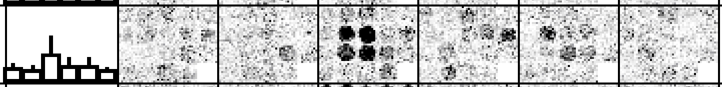							0.97
J05181	gamma-glutamylcysteine synthetase	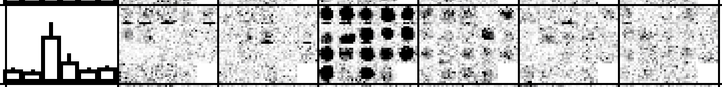							0.97
NM_021583	inducible prostaglandin e synthase ipges; pig12; pges; pge; ptges	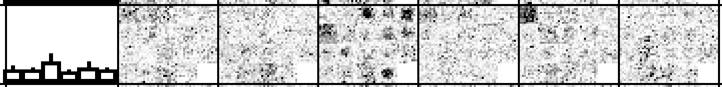							0.97
NM_019214	pendrin pds	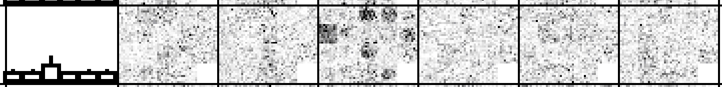							0.97
mwgrat10K# 7928	expression: liver kidney brain; strains: shrsp sprague_dawley;	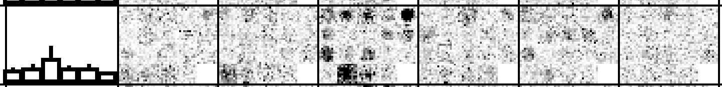							0.97
NM_030837	kidney specific organic anion transporter slc21a4; transporter-k2	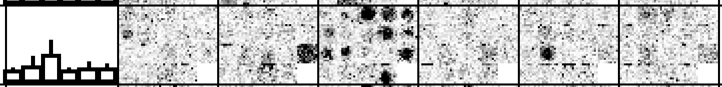							0.96
NM_022251	aminopeptidase a enpep	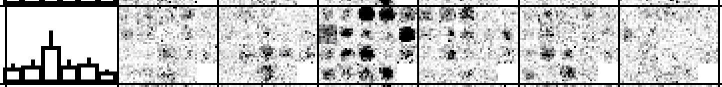							0.96
mwgrat10K# 7278	expression: brain kidney; strains: shrsp wistar_kyoto; similar to	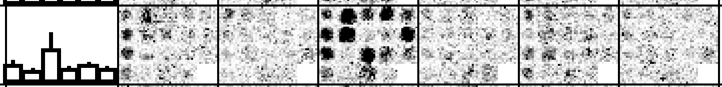							0.96
mwgrat10K# 8788	expression: kidney; strains: wistar_kyoto; similar to	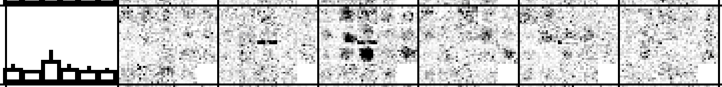							0.96
mwgrat10K# 7609	expression: kidney; strains: shrsp wistar_kyoto; similar to	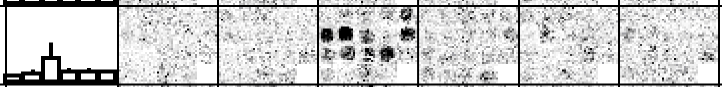							0.96
U39555	eaac1	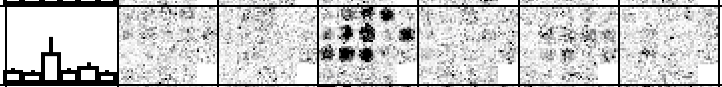							0.96
mwgrat10K# 8764	expression: kidney; strains: wistar_kyoto; similar to	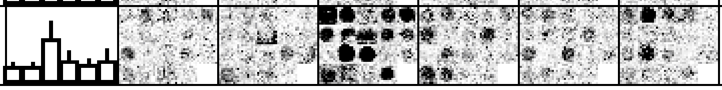							0.96

GeneID	GeneName	Signals	Brain	Heart	Kidney	Liver	Lung	Spleen	OSI
NM_019134	solute carrier family 12, member 1 bumetanide-sensitive								0.96
NM_012789	dipeptidyl peptidase 4; dpp4								0.95
AJ000555	interleukin-3 receptor beta-subunit ril-3r<beta>								0.95
mwgrat10K#7249	expression: brain kidney; strains: shrsp wistar_kyoto; similar to								0.95
AF155822	kinesin heavy chain								0.95
mwgrat10K#8885	expression: kidney; strains: wistar_kyoto; similar to								0.95
mwgrat10K#7586	expression: kidney; strains: shrsp wistar_kyoto; similar to pir								0.95
mwgrat10K#7986	expression: brain kidney; strains: shrsp; similar to								0.95
NM_017021	interleukin 9 receptor il9r; gfi-2								0.95
S76742	neurotransmitter transporter rb21a								0.95
NM_053314	potassium inwardly-rectifying channel, subfamily j, member 16								0.95
mwgrat10K#7352	expression: liver; strains: wistar_kyoto; similar to								0.95
mwgrat10K#7633	expression: kidney; strains: shrsp wistar_kyoto; similar to								0.94
M31051	heymann nephritis antigen gp330; 45kda protein								0.94
NM_017305	glutamate-cysteine ligase, modifier subunit gclm;								0.94
AF187100	putative n-acetyltransferase cml5 cml5								0.94
AJ238391	sulfotransferase k1 sultk1; k2 sultk2								0.94
AB000717	non-hepatic-type s-adenosylmethionine synthetase;								0.94
NM_031588_1	glial growth factor beta 1a nrg1; smdf neuregulin; alpha 2a; 1; neu								0.94
AJ300163	uncoupling protein ucp-4, isoform b ucp-4								0.94
NM_024399	aspartoacylase aspa								0.94
mwgrat10K#7207	expression: liver kidney; strains: shrsp sprague_dawley wistar_kyoto;								0.94
mwgrat10K#8092	expression: kidney; strains: shrsp; similar to gbp|bc012407|bc012407_1								0.93

GeneID	GeneName	Signals	Brain	Heart	Kidney	Liver	Lung	Spleen	OSI
mwgrat10K#7615	expression: kidney brain; strains: shrsp sprague_dawley wistar_kyoto;								0.93
NM_030998	anti-mullerian hormone type 2 receptor amhr2; mis ii ratmrii; put.								0.93
NM_012960	gamma-glutamyl hydrolase precursor; ggh								0.93
NM_013103	transcription factor 2, hepatic lf-b3 variant hepatic nuclear factor tcf2; 1								0.92
NM_013085	urinary plasminogen activator, urokinase plau; urikinase-type								0.92
mwgrat10K#7545	expression: kidney; strains: shrsp wistar_kyoto; similar to								0.92
mwgrat10K#9613	expression: kidney; strains: shrsp; similar to gbp|u73199|u73199_1								0.92
mwgrat10K#8036	expression: kidney brain; strains: shrsp; similar to								0.91
J02997	bile canaliculus-specific membrane glycoprotein precursor								0.91
mwgrat10K#6702	expression: kidney heart; strains: shrsp wistar_kyoto; similar to								0.91
mwgrat10K#6865	expression: heart; strains: shrsp sprague_dawley wistar_kyoto;								0.91
mwgrat10K#8223	expression: brain; strains: shrsp; similar to gbp|ak006147|ak006147_1								0.90
mwgrat10K#5757	expression: kidney heart brain; strains: shrsp sprague_dawley								0.90
mwgrat10K#7602	expression: kidney; strains: shrsp wistar_kyoto; similar to								0.90
NM_031341	solute carrier family 7 cationic amino acid transporter, y??, member 7								0.90
mwgrat10K#7524	expression: kidney; strains: wistar_kyoto; similar to								0.89
NM_024159_1	doc-2 p82 isoform dab2; p59								0.89
AF044574	putative peroxisomal 2,4-dienoyl-coa reductase dcr-akl								0.89
NM_017129	cardiotrophin 1 ctf1; cardiotrophin-1								0.89
mwgrat10K#7572	expression: liver kidney heart; strains: shrsp sprague_dawley								0.89
NM_053307	methionine sulfoxide reductase a; msra								0.88
mwgrat10K#8895	expression: kidney; strains: wistar_kyoto; similar to								0.88
NM_031036	heterotrimeric guanine nucleotide-binding protein alpha q								0.88

GeneID	GeneName	Signals	Brain	Heart	Kidney	Liver	Lung	Spleen	OSI
mwgrat10K# 7893	expression: liver kidney brain; strains: shrsp sprague_dawley;								0.87
mwgrat10K# 8102	expression: kidney; strains: shrsp; similar to pir nf00077973 cytosolic								0.87
mwgrat10K# 9263	expression: brain; strains: shrsp; similar to gbp|ak006147|ak006147_1								0.87
S62903	genetic hypertension component sa								0.87
NM_012756	mannose 6-phosphate/insulin-like growth factor ii receptor m6p/igf2r;								0.86
mwgrat10K# 9681	expression: kidney; strains: wistar_kyoto; mwg own new gene								0.84
NM_016999	cytochrome p450, subfamily ivb, polypeptide 1 see also d5m4rp1								0.84
mwgrat10K# 8990	expression: brain; strains: sprague_dawley; similar to pir								0.83
mwgrat10K# 9573	expression: kidney; strains: shrsp; similar to gbp|bc011417|bc011417_1								0.83
NM_019908	hypothetical protein loc56728 loc56728; unknown; kidney-specific								0.82
mwgrat10K# 8098	expression: kidney; strains: shrsp; similar to gbp|ak020131|ak020131_1								0.81
mwgrat10K# 8811	expression: kidney; strains: wistar_kyoto; similar to								0.81
U08031	sodium dependent sulfate transporter; na/sulfate cotransporter								0.81
NM_017135	adenylate kinase 4 ak4								0.80
NM_012770 _1	guanylate cyclase, soluble, beta 2; gtp pyrophosphate - lyase; gucy1b2								0.80
mwgrat10K# 8315	expression: kidney brain; strains: sprague_dawley wistar_kyoto; mwg								0.78
NM_017081	hydroxysteroid dehydrogenase, 11 beta type 2 hsd11b2;								0.78
NM_022238	tap-like abc transporter abcb9; tapl								0.78
X13722	ldl-receptor precursor aa -21 to 879								0.78
mwgrat10K# 8527	expression: brain; strains: wistar_kyoto; similar to								0.77
mwgrat10K# 8808	expression: kidney; strains: wistar_kyoto; similar to								0.77
NM_017349	sodium/potassium-transporting atpase gamma chain; na,k-atpase								0.75
S48142_1	t-kininogenase; kallikrein								0.75

GeneID	GeneName	Signals	Brain	Heart	Kidney	Liver	Lung	Spleen	OSI
NM_012881	sialoprotein osteopontin spp1; precursor	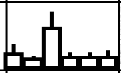	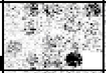	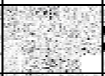	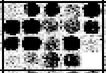	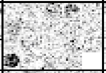	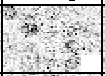	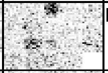	0.73
mwgrat10K#9605	expression: kidney; strains: shrsp; similar to gbp|bc003800|bc003800_1	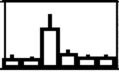	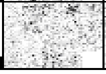	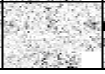	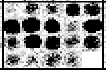	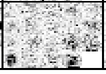	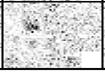	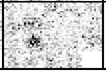	0.72

GeneID	GeneName	Signals	Brain	Heart	Kidney	Liver	Lung	Spleen	OSI
NM_022220	l-gulono-gamma-lactone oxidase precursor; gulo	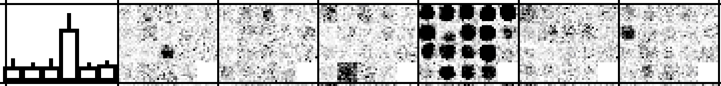							1.00
NM_023988	aj18 protein aj18	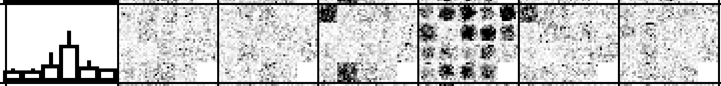							1.00
NM_031344	delta-6 fatty acid desaturase fads2	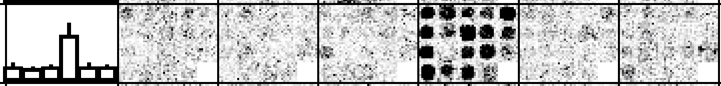							1.00
NM_020538	arylacetamide deacetylase loc57300	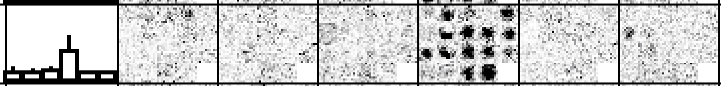							1.00
NM_019346	solute carrier family 14, member 1 slc14a1; urea transporter ut3; ut11	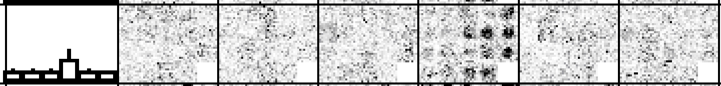							1.00
NM_019369	inter-alpha-inhibitor h4 heavy chain itih4	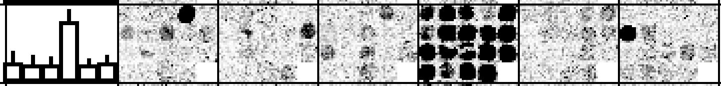							1.00
NM_020089	dnaj-like protein loc56764	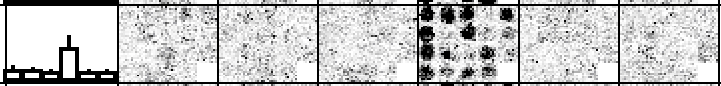							1.00
NM_031531	contrapsin-like protease inhibitor related protein cpi-23; serine spin2c;	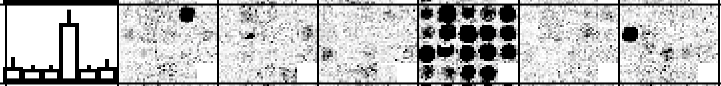							1.00
NM_053617	carboxypeptidase b2 plasma cpb2; pre-procarboxypeptidase r	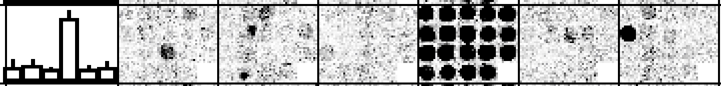							1.00
NM_053753	carbohydrate-binding receptor; kupffer cell; kclr	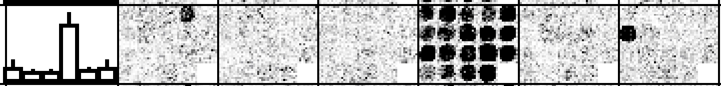							1.00
NM_057146	complement component c9 precursor; 9	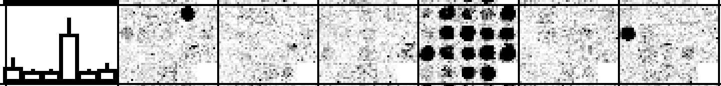							1.00
NM_053379	doublecortin dcx	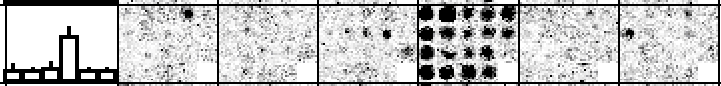							1.00
NM_031533	androsterone udp-glucuronosyltransferase ugt2b2;	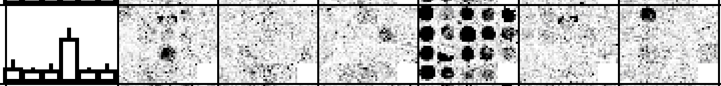							1.00
NM_031565	carboxylesterase es-3 egasyn; 1 ces1	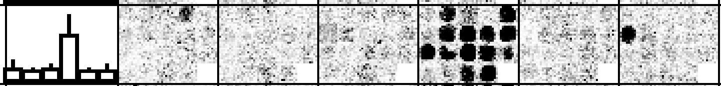							1.00
NM_053288	alpha1-acid glycoprotein precursor; alpha-1 acid; alpha-1-acid;	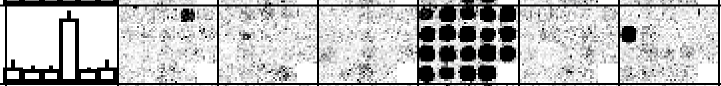							1.00
NM_019280	gap junction membrane channel protein alpha 5 gja5; connexin 40;	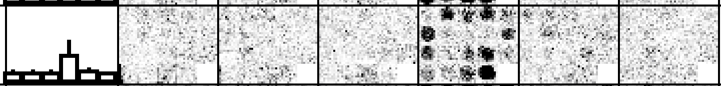							1.00
NM_012738	preproapolipoprotein a-i; preapolipoprotein; apolipoprotein	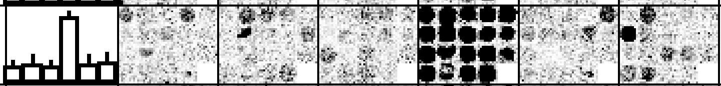							1.00
NM_012826	zn-alpha2-glycoprotein precursor; zn - alpha2 glycoprotein azgp1;	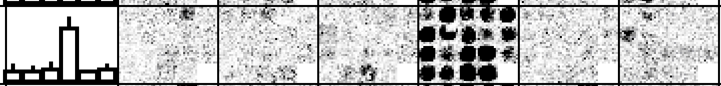							1.00
NM_013003	phosphatidylethanolamine n-methyltransferase pemt	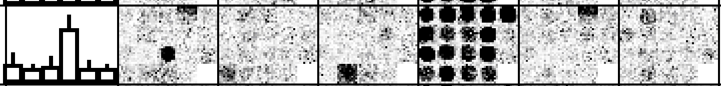							1.00
NM_012737	apolipoprotein a-iv prepeptide; preapolipoprotein; c-iv apoa4	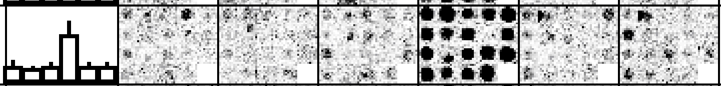							1.00
NM_012599	mannose binding protein a, serum mbpa; mannose-binding; a mbp	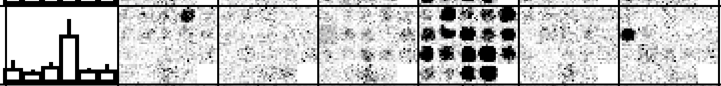							1.00
NM_012668	tyrosine aminotransferase ec 2.6.1.5; tat; aa 1-454	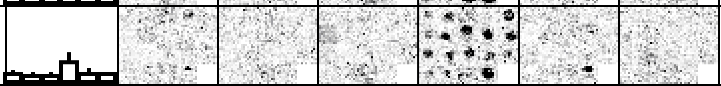							1.00
NM_012725	plasma kallikrein klk3; precursor; rpk; pk	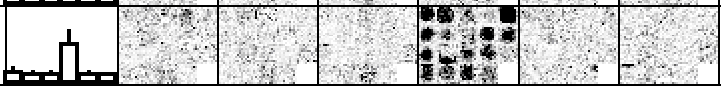							1.00

GeneID	GeneName	Signals	Brain	Heart	Kidney	Liver	Lung	Spleen	OSI
NM_013119	sodium channel, voltage-gated, type iii, alpha polypeptide scn3a; channel								1.00
NM_017134	liver arginase e.c. 3.5.3.1; 1, arg1								1.00
NM_017158	cytochrome p450, 2c39 cyp2c39; p450; p450f								1.00
NM_017300	bile acid-coenzyme a dehydrogenase: amino acid								1.00
NM_017072	carboamyl-phosphate synthetase 1 cps1; carbamyl phosphate cps								1.00
NM_016995	complement component 4 binding protein, beta c4bpb; c4bp chain,								1.00
NM_017019	interleukin-1 alpha il-1 alpha; 24 proil-1; interleukin 1 il1a								1.00
NM_017047	solute carrier family 10 sodium/bile acid cotransporter family, member 1								1.00
mwgrat10K#7315	expression: liver; strains: shrsp sprague_dawley wistar_kyoto;								1.00
mwgrat10K#7338	expression: liver; strains: shrsp wistar_kyoto; similar to pir								1.00
mwgrat10K#7431	expression: liver; strains: shrsp sprague_dawley wistar_kyoto;								1.00
mwgrat10K#6848	expression: liver; strains: shrsp sprague_dawley wistar_kyoto;								1.00
mwgrat10K#6761	expression: liver; strains: shrsp sprague_dawley wistar_kyoto;								1.00
mwgrat10K#6765	expression: liver; strains: wistar_kyoto; similar to								1.00
mwgrat10K#6796	expression: liver; strains: shrsp sprague_dawley wistar_kyoto;								1.00
mwgrat10K#7514	expression: liver; strains: sprague_dawley wistar_kyoto;								1.00
mwgrat10K#9179	expression: liver; strains: sprague_dawley; similar to								1.00
mwgrat10K#9322	expression: brain; strains: shrsp; similar to pir nf00138209 kiaa1164								1.00
mwgrat10K#9664	expression: liver; strains: shrsp; mwg own new gene sequence								1.00
mwgrat10K#8708	expression: liver; strains: wistar_kyoto; similar to								1.00
mwgrat10K#8370	expression: liver; strains: shrsp; similar to pir nf00506480								1.00
mwgrat10K#8407	expression: liver; strains: shrsp; similar to gbp|ab060274|ab060274_1								1.00
mwgrat10K#8435	expression: liver; strains: wistar_kyoto; similar to pir								1.00

GeneID	GeneName	Signals	Brain	Heart	Kidney	Liver	Lung	Spleen	OSI
mwgrat10K#6757	expression: liver; strains: shrsp sprague_dawley wistar_kyoto;								1.00
U39206	cytochrome p450 4f4 cyp4f4								1.00
U94856	paraoxonase								1.00
X06108	insulin-like growth factor precursor; igf; igf-i aa 1-159								1.00
U33501	retinol dehydrogenase type iii; i								1.00
NM_080576	apolipoprotein a-v apoa-v; apoa5								1.00
S82911	rhox protein rhox								1.00
U21954	ehk-3, full length form								1.00
X15734	s-adenosylmethionine synthetase								1.00
Y18572	mannose-binding protein associated serine protease-2 masp-2; mannose								1.00
mwgrat10K#6404	expression: brain heart kidney; strains: shrsp wistar_kyoto; similar								1.00
mwgrat10K#6405	expression: liver heart; strains: shrsp sprague_dawley wistar_kyoto;								1.00
X86561_1	fibrinogen alpha-e subunit								1.00
X17621	put. rck2 protein aa 1-530								1.00
X69834	serine protease inhibitor 2.4								1.00
X81301	variable region-alpha rva19								1.00
NM_012541	cytochrome p450, subfamily i aromatic compound-inducible,								1.00
M33329	hydroxysteroid sulfotransferase a; sta								1.00
M24396	urate oxidase 2; ec 1.7.3.3								1.00
M17592	pre-pro-insulin-like growth factor i								1.00
M35086	udp-glucuronosyltransferase ec 2.4.1.17; udp								1.00
NM_012503	asialoglycoprotein receptor 1 hepatic lectin asgr1								1.00
AF295535	amino acid transport system a3 ata3								1.00

GeneID	GeneName	Signals / Brain / Heart / Kidney / Liver / Lung / Spleen	OSI
M35602	beta-fibrinogen	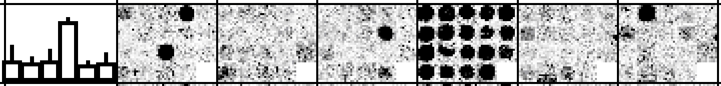	1.00
M13646	testosterone-6beta-hydroxylase 6 beta-a cyp3a2; cytochrome p450;	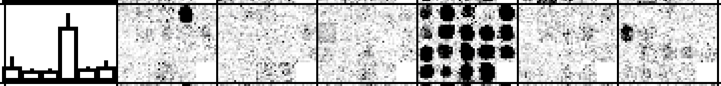	1.00
D30620	carboxylesterase precursor ec 3.1.1.1; carboxyesterase e1; serum	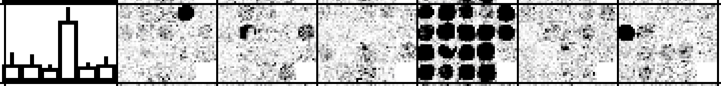	1.00
AJ007485	hypothetical protein	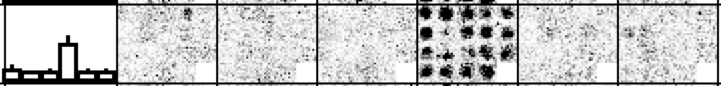	1.00
AF380198	trace amine receptor 10; ta10	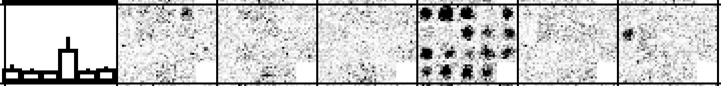	1.00
D50580	carboxylesterase precursor	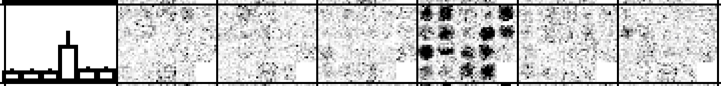	1.00
AB057450	alpha-amylase amy1	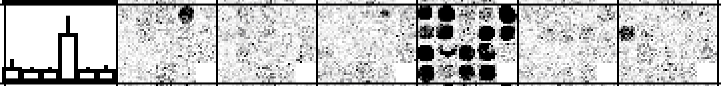	1.00
M11227	apolipoprotein b pi	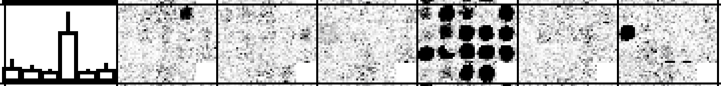	1.00
J02585	stearyl-coa desaturase	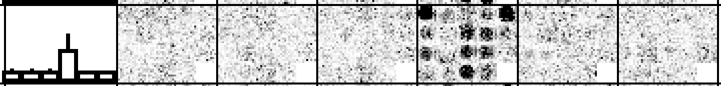	1.00
NM_053019	vasopressin receptor v1a; avpr1a	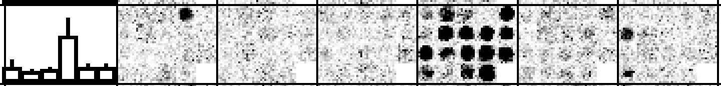	0.99
L13207_1	hnf-3/fork-head homolog-7	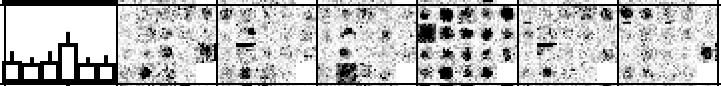	0.99
NM_053318	hemopexin hpxn	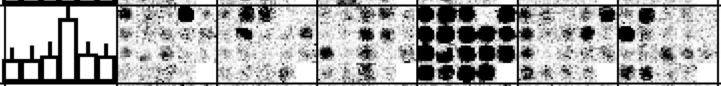	0.99
mwgrat10K# 8414	expression: liver; strains: shrsp; similar to pir nf00515379	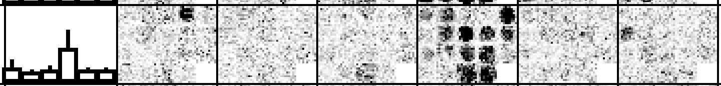	0.99
AF091577	olfactory receptor	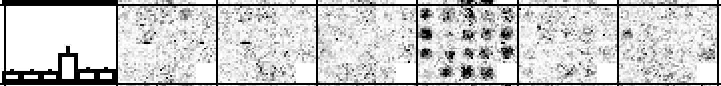	0.99
NM_031815	activin beta e inhbe	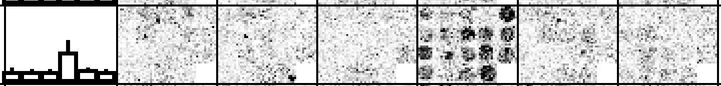	0.99
NM_053405	potassium channel, subfamily k, member 9 task-3 kcnk9; channel	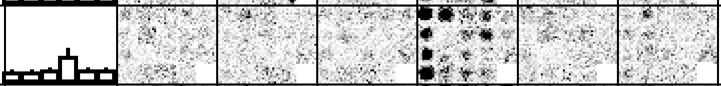	0.99
mwgrat10K# 6784	expression: liver heart; strains: shrsp sprague_dawley wistar_kyoto;	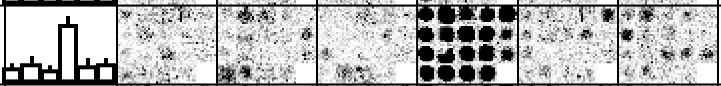	0.99
mwgrat10K# 6781	expression: liver; strains: shrsp sprague_dawley wistar_kyoto;	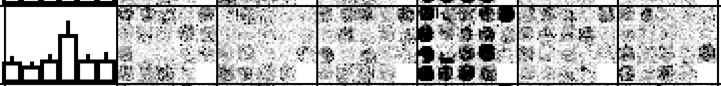	0.99
mwgrat10K# 7382	expression: liver; strains: sprague_dawley wistar_kyoto;	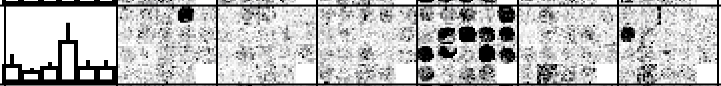	0.99
mwgrat10K# 7914	expression: liver; strains: shrsp sprague_dawley; similar to pir	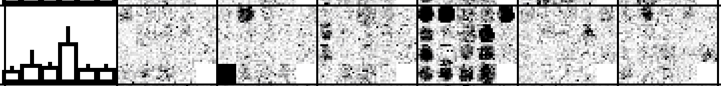	0.99
NM_053785	transmembrane 4 superfamily member 4 tm4sf4; tetraspan protein	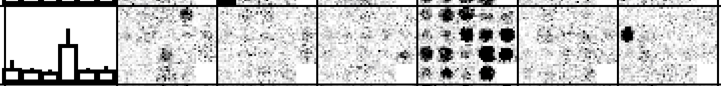	0.99
mwgrat10K# 7437	expression: liver; strains: shrsp sprague_dawley wistar_kyoto;	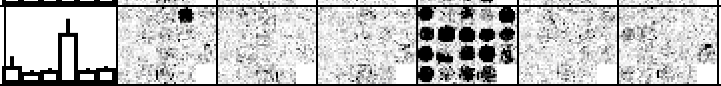	0.99
mwgrat10K# 6802	expression: liver; strains: shrsp sprague_dawley wistar_kyoto;	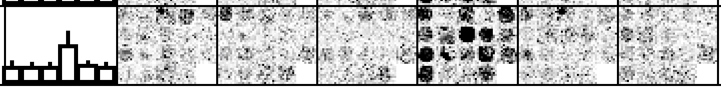	0.99

GeneID	GeneName	Signals	Brain	Heart	Kidney	Liver	Lung	Spleen	OSI
NM_022924	preprothrombin aa -43 to 574; thrombin; coagulation factor ii f2								0.99
NM_022929	potassium channel interacting protein 1 kchip1; kcnip1								0.99
NM_022960	neutral solute channel aquaporin 9 aqp9								0.99
NM_022234	putative acid-sensing ion channel asic4; spasic protein								0.99
NM_022303	caspase recruitment domain protein 9 loc64171								0.99
NM_022403	tryptophan-2,3-dioxygenase tdo2								0.99
NM_023091	gamma-aminobutyric acid; gaba-a receptor, subunit epsilon; gabre								0.99
NM_030850	betaine homocysteine methyltransferase bhmt;								0.99
mwgrat10K#<br>6822	expression: liver brain; strains: shrsp sprague_dawley wistar_kyoto;								0.99
NM_012584	hydroxy-delta-5-steroid dehydrogenase, 3 beta- and steroid								0.99
NM_023103	alpha 1 -inhibitor 3, variant i mug1; alpha-1-inhibitor iii precursor;								0.99
NM_024143	bile acid coa ligase loc79111								0.99
NM_024391	17-beta hydroxysteroid dehydrogenase type 2 hsd17b2								0.99
AJ320522	complement inhibitory factor h; fh								0.99
X79991	cytochrome p450iii cyp3; p450 6beta-2								0.99
AJ277423	mannose-binding protein associated serine protease-1 masp-1								0.99
X15741	rat polymeric immunoglobulin receptor aa -18 to 751								0.99
AY012054	zinc finger protein zfx zfx								0.99
mwgrat10K#<br>6763	expression: liver; strains: shrsp sprague_dawley wistar_kyoto;								0.99
AF194029	histidine-rich glycoprotein 1 rnhrg1; 2 rnhrg2; hrg								0.99
NM_012556	fatty acid binding protein 1, liver fabp1; fabp; sterol carrier								0.99
AF380186	trace amine receptor 1; ta1								0.99
mwgrat10K#<br>6762	expression: liver; strains: shrsp wistar_kyoto; similar to								0.99

GeneID	GeneName	Signals	Brain	Heart	Kidney	Liver	Lung	Spleen	OSI
X95189	trihydroxycoprostanoyl-coa oxidase thcox	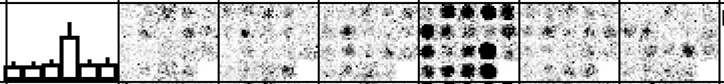							0.99
NM_012559	fibrinogen, gamma polypeptide fgg; gamma-fibrinogen	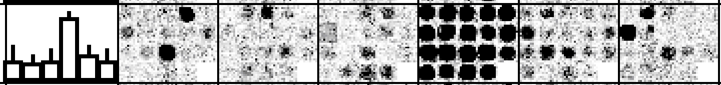							0.99
mwgrat10K#6420	expression: liver heart brain kidney; strains: shrsp sprague_dawley	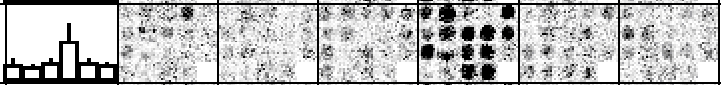							0.99
X15679	preprotrypsinogen iv aa -15 to 232	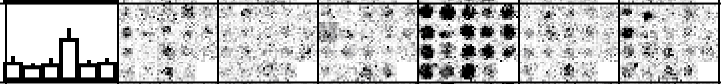							0.99
D17309	delta-4-3-ketosteroid 5-beta-reductase; delta	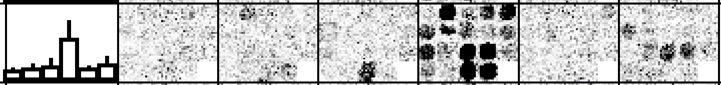							0.99
U39450	vasopressin v1a receptor	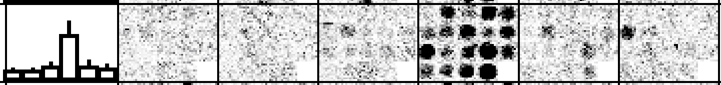							0.99
U51017	kallistatin	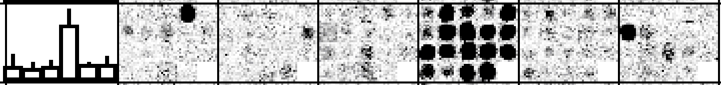							0.99
NM_012579	h1 histone family, member t testis-specific h1t; variant	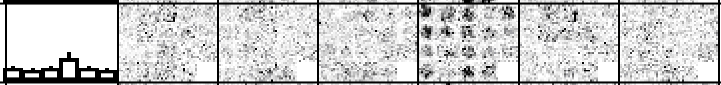							0.99
U17375	cytochrome p-450c27/25	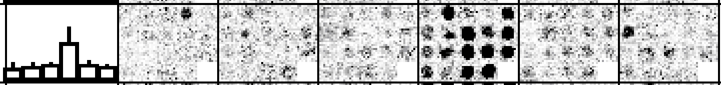							0.99
AF347056	glycoprotein tec-21	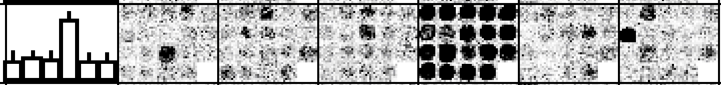							0.99
U55765	rasp1	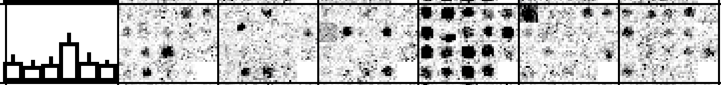							0.99
D12678	insulin receptor-related receptor	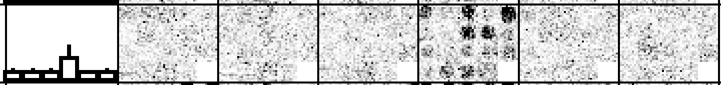							0.99
X13149	contrapsin-like protease inhibitor related protein cpi-23; serine 1;	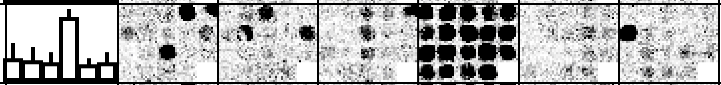							0.99
X15551	beta-2 glycoprotein i aa 1-297	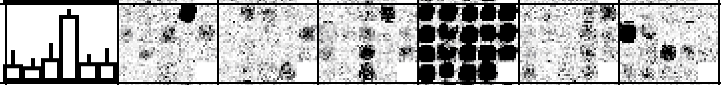							0.99
U83666	cell cycle checkpoint protein kinase bub1 rbub1	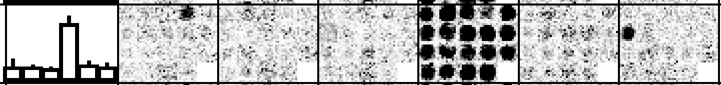							0.99
D14987	hydroxysteroid sulfotransferase	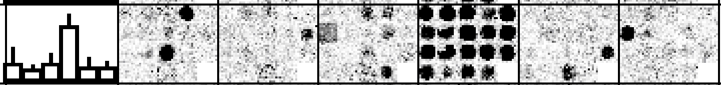							0.99
V01255	steroid-binding protein c1; reading frame; c-1 polypeptide	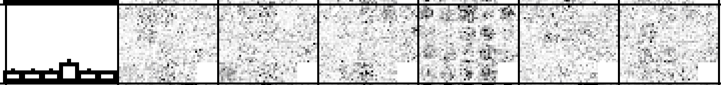							0.99
M27440	apolipoprotein b apob	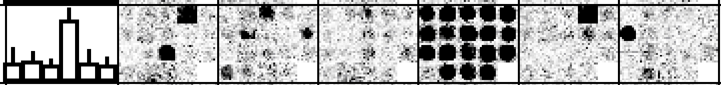							0.99
mwgrat10K#8717	expression: liver; strains: wistar_kyoto; similar to	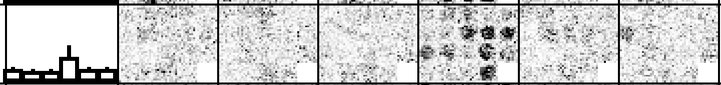							0.99
NM_017096	c-reactive protein, member of the pentraxin family crp; protein	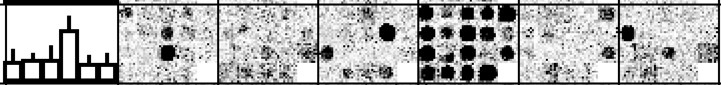							0.99
mwgrat10K#9168	expression: liver; strains: sprague_dawley; similar to	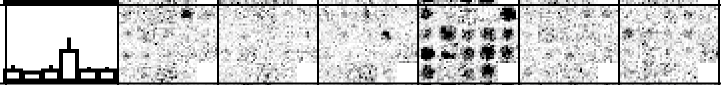							0.99
NM_012657	serine protease inhibitor; spin2b	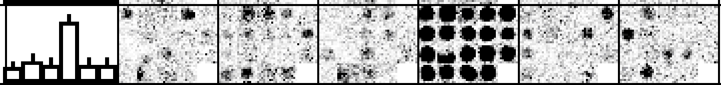							0.99
NM_017189	asialoglycoprotein receptor 2 asgr2; rhl2; hepatic lectin aa 1-301	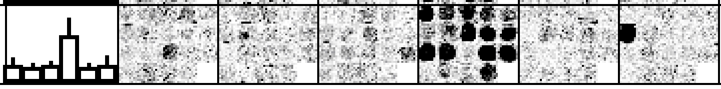							0.99

GeneID	GeneName	Signals	Brain	Heart	Kidney	Liver	Lung	Spleen	OSI
NM_012646 _1	rt1 class ib gene, h2-tl-like, grc region rt1-n1; germline mhc i; cell	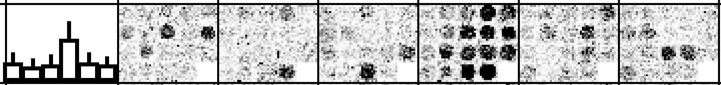							0.99
NM_017334	transcriptional repressor crem transcriptional repressor crem, icer	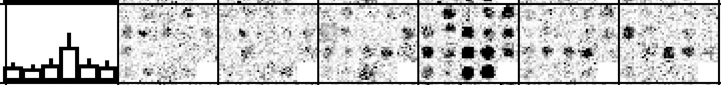							0.99
mwgrat10K# 8703	expression: liver; strains: wistar_kyoto; similar to pir	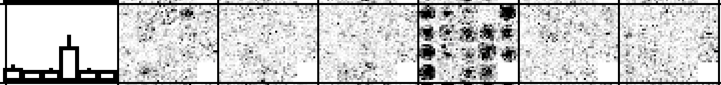							0.99
AB055895	histidine-rich glycoprotein 1 rnhrg1; 2 rnhrg2	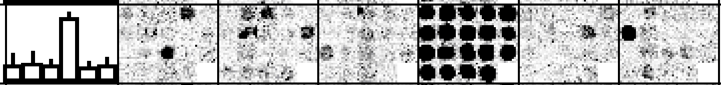							0.99
mwgrat10K# 8707	expression: liver; strains: wistar_kyoto; similar to pir	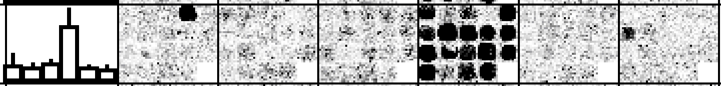							0.99
M63991	thyroxine-binding globulin tbg	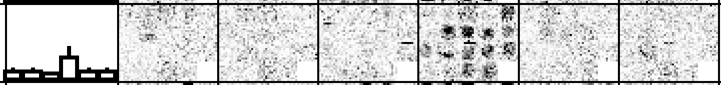							0.99
NM_012898	59-kda bone sialic acid-containing protein precursor; phosphorylated	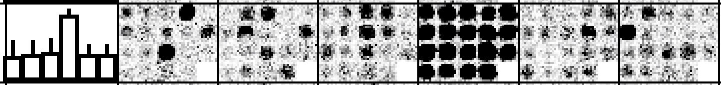							0.99
NM_012695	rat senescence marker protein 2a gene, exons 1 and 2; smp2a	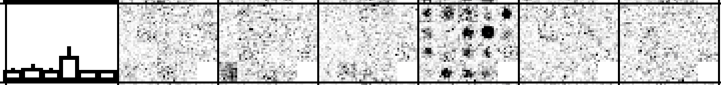							0.99
AB040609	cytoplasmic acetyl-coa hydrolase rach	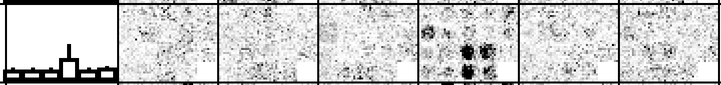							0.99
mwgrat10K# 9191	expression: brain; strains: shrsp; similar to gbp|bc015288|bc015288_1	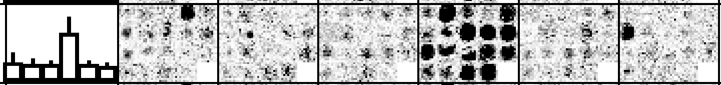							0.99
NM_012693	cytochrome p450 iia1 hepatic steroid hydroxylase iia1 gene cyp2a1;	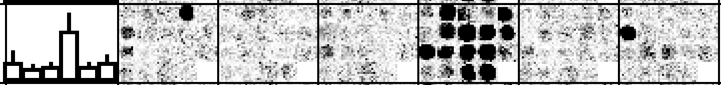							0.99
NM_012690	atp-binding cassette, sub-family b mdr/tap, member 4 p-glycoprotein 3/	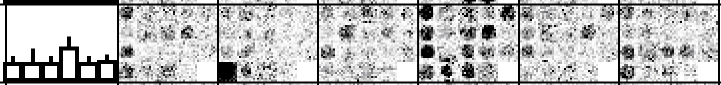							0.99
NM_017017	hepatocyte growth factor scatter factor hgf; prepropeptide; precursor	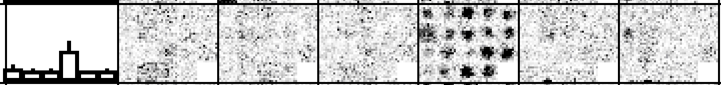							0.99
NM_013217	afadin af-6; l-afadin	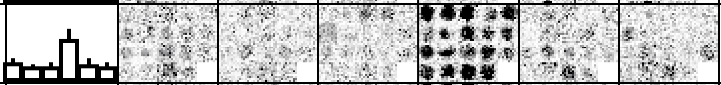							0.99
M58041	cytochrome p-450md aa 1-489; p-450	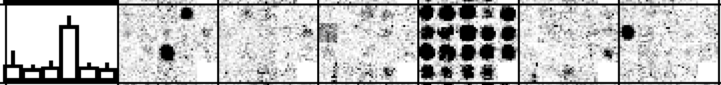							0.99
NM_013112	pre-pro-apolipoprotein -23 to 79; apolipoprotein a-ii apoa-ii; apoa2	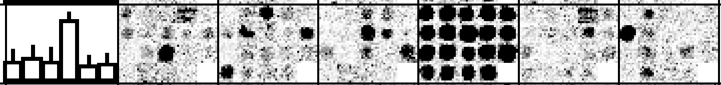							0.99
NM_019158	aquaporin-pancreas and liver aqp8; aquaporin 8	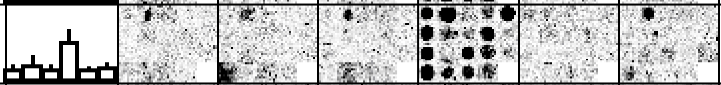							0.99
NM_019191	mad homolog 2 drosophila madh2; smad2; protein	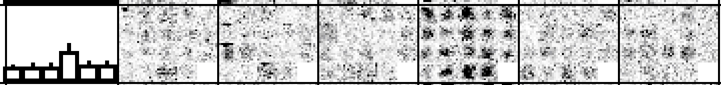							0.99
mwgrat10K# 8682	expression: liver; strains: sprague_dawley; similar to	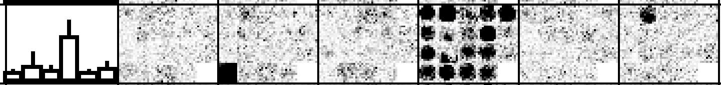							0.99
M13711	cytochrome p450 pb1	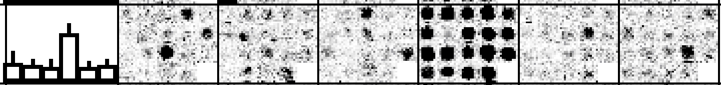							0.99
mwgrat10K# 6858	expression: liver; strains: shrsp wistar_kyoto; similar to	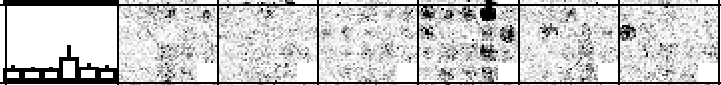							0.99
M22329	cytochrome p450 iid5 protein p450 iid5; cyp2d1; p-450 iid1;	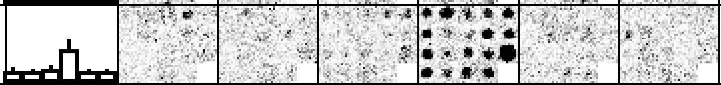							0.99
NM_012532 _1	ceruloplasmin ferroxidase cp; gpi-anchored	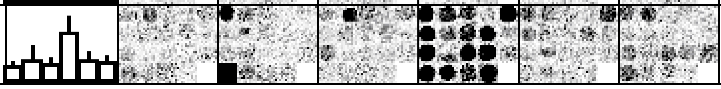							0.99

GeneID	GeneName	Signals	Brain	Heart	Kidney	Liver	Lung	Spleen	OSI
NM_012597	hepatic lipase precursor; lipase, lipc; aa 1-494	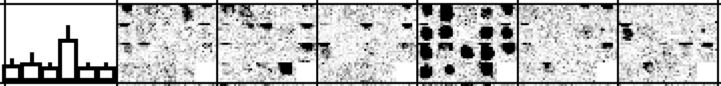							0.99
mwgrat10K# 8653	expression: liver; strains: wistar_kyoto; similar to	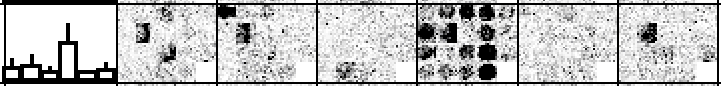							0.99
NM_021696	plasminogen activator inhibitor 2 type a pai2a	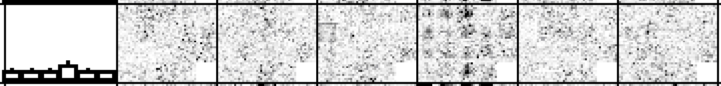							0.99
mwgrat10K# 6839	expression: liver; strains: shrsp sprague_dawley wistar_kyoto;	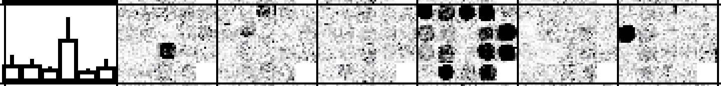							0.99
X80535	thyrotropin-releasing hormone degrading enzyme	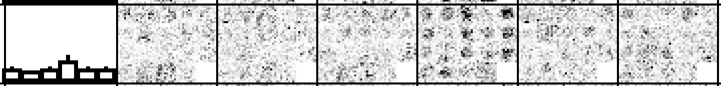							0.98
AF177687	protocadherin alpha 4 homolog	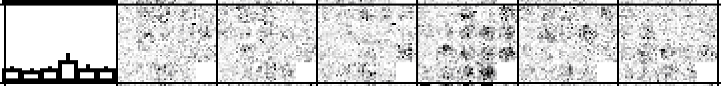							0.98
mwgrat10K# 6791	expression: liver kidney; strains: shrsp sprague_dawley wistar_kyoto;	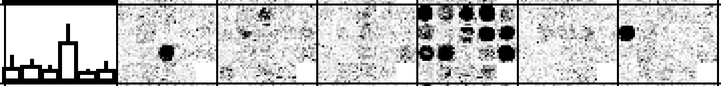							0.98
mwgrat10K# 7379	expression: liver; strains: shrsp sprague_dawley wistar_kyoto;	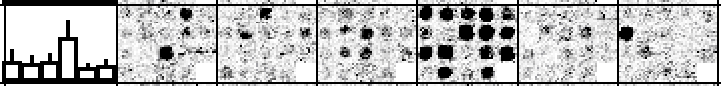							0.98
mwgrat10K# 7374	expression: liver; strains: shrsp sprague_dawley wistar_kyoto;	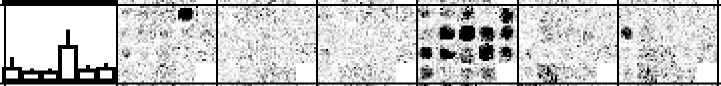							0.98
AB000199	cca2 protein cca2	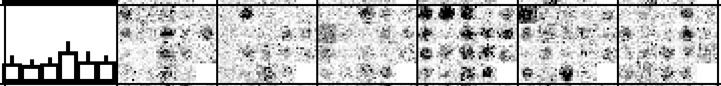							0.98
U39208	cytochrome p450 4f6 cyp4f6	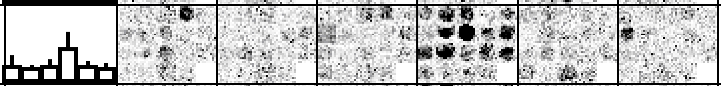							0.98
mwgrat10K# 9180	expression: liver; strains: sprague_dawley; similar to	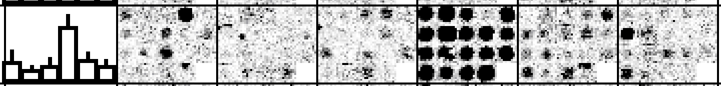							0.98
S79292_2	rat hypoxanthine-guanine phosphoribosyltransferase hprt;	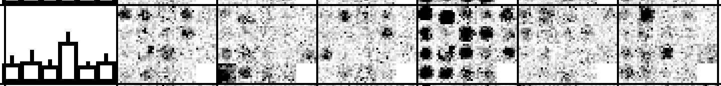							0.98
M17593	pre-pro-insulin-like growth factor i	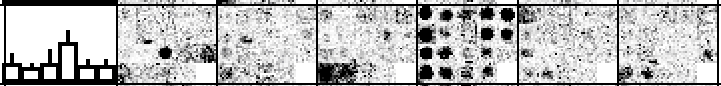							0.98
Y18571	mannose-binding protein associated serine protease-2 masp-2	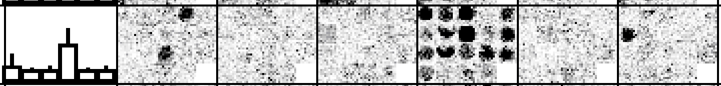							0.98
NM_053995	3-hydroxybutyrate dehydrogenase heart, mitochondrial bdh;	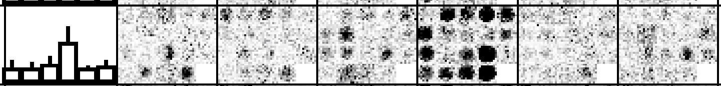							0.98
mwgrat10K# 8686	expression: liver; strains: wistar_kyoto; similar to pir	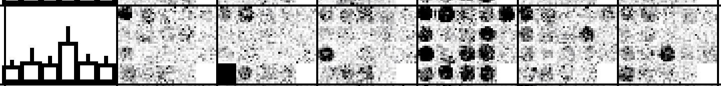							0.98
M24239	product of unknown function	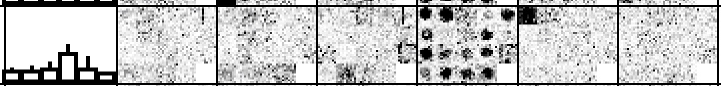							0.98
mwgrat10K# 7922	expression: liver; strains: shrsp sprague_dawley; similar to	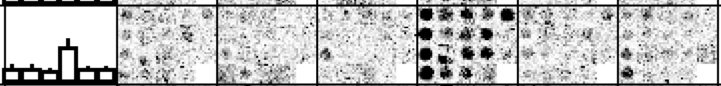							0.98
NM_053348	fetuin-like protein irl685 irl685; fetuin beta fetub	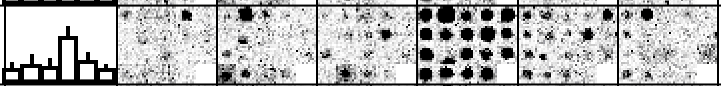							0.98
mwgrat10K# 8295	expression: liver; strains: sprague_dawley wistar_kyoto; mwg	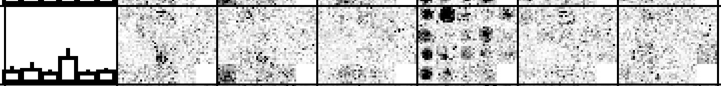							0.98
mwgrat10K# 9178	expression: liver; strains: sprague_dawley; similar to pir	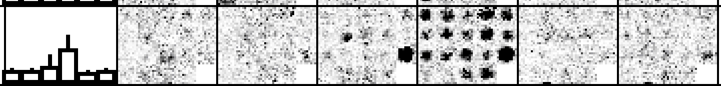							0.98
NM_052980	nuclear receptor subfamily 1, group i, member 2 nr1i2; pregnane x	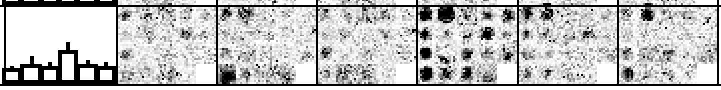							0.98

GeneID	GeneName	Signals	Brain	Heart	Kidney	Liver	Lung	Spleen	OSI
NM_022704	mannose-binding protein c liver mbl2; precursor; mbp aa -18 to 226	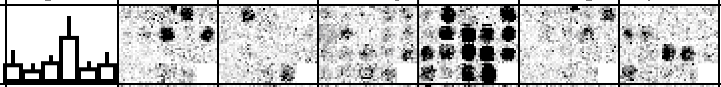							0.98
AF092091	cp431	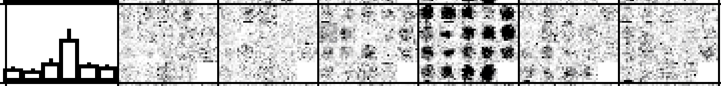							0.98
mwgrat10K#7919	expression: liver; strains: shrsp sprague_dawley; similar to	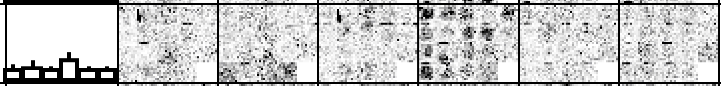							0.98
M38566	vitamin d 3 25-hydroxylase precursor cyp27; cytochrome p450 p450c27;	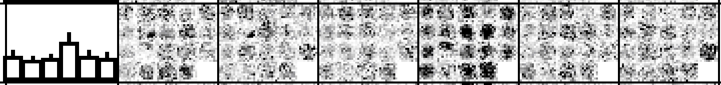							0.98
NM_030656	mitochondrial serine:pyruvate aminotransferase precursor;	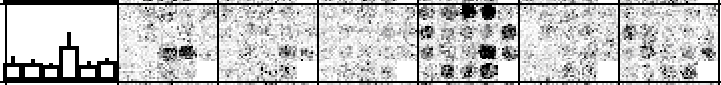							0.98
NM_022380	signal transducer and activator of transcription 5b stat5b	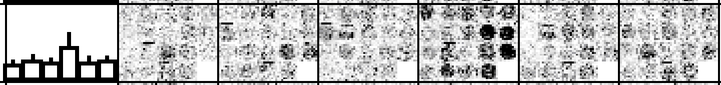							0.98
D00577	follicle-stimulating hormone beta-subunit; fsh	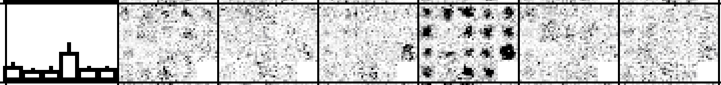							0.98
NM_023963	cdx2 protein cdx2	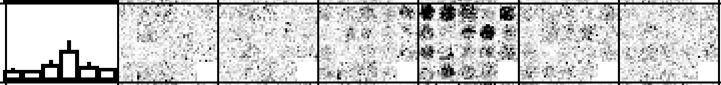							0.97
mwgrat10K#9177	expression: liver; strains: sprague_dawley; similar to pir	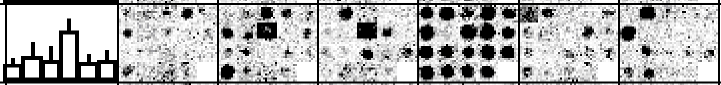							0.97
mwgrat10K#6924	expression: liver brain; strains: shrsp sprague_dawley wistar_kyoto;	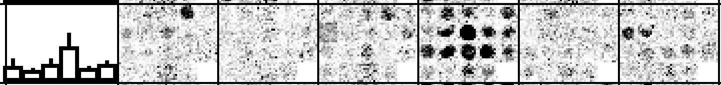							0.97
NM_016994	pre-pro-complement c3 aa 1663; complement component; 3	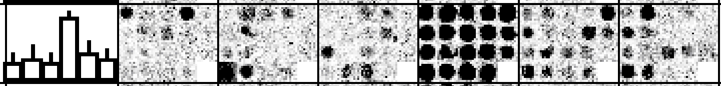							0.97
NM_022614	activin/inhibin beta c inhbc; activin	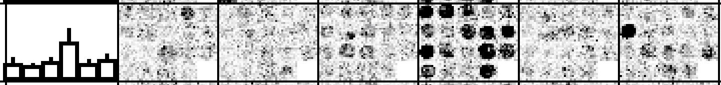							0.97
NM_057101	cytochrome p450, 21, steroid 21 hydroxylase cyp21; 21-hydroxylase	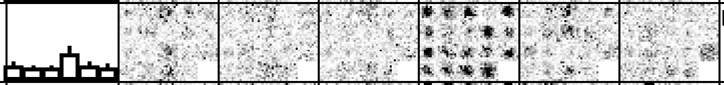							0.97
M26125	epoxide hydrolase	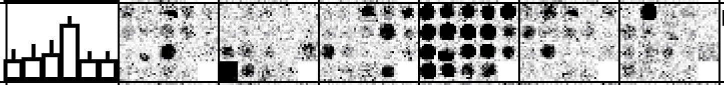							0.97
mwgrat10K#9345	expression: brain; strains: shrsp; similar to gbp|ak019371|ak019371_1	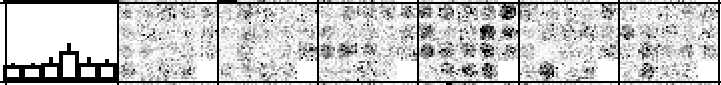							0.97
mwgrat10K#6845	expression: liver; strains: shrsp sprague_dawley; similar to	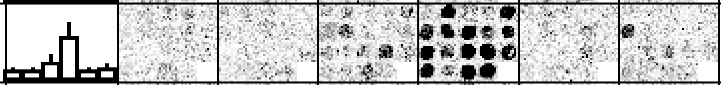							0.97
NM_053375	hyperpolarization-activated, cyclic nucleotide-gated potassium channel	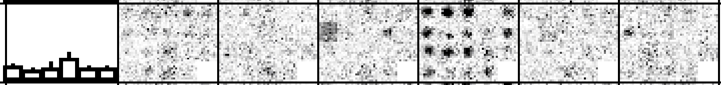							0.97
mwgrat10K#7924	expression: liver brain heart; strains: shrsp sprague_dawley; similar to	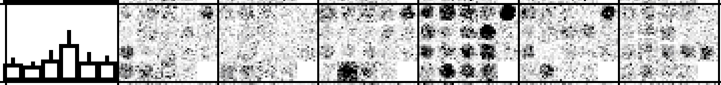							0.97
AF436847	complement factor h-related protein	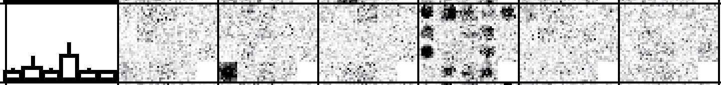							0.97
M95762	gaba transporter	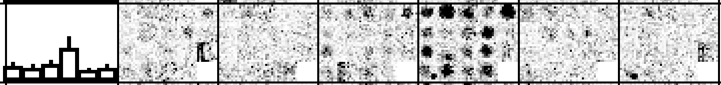							0.97
U20194	complement c8 beta c8b	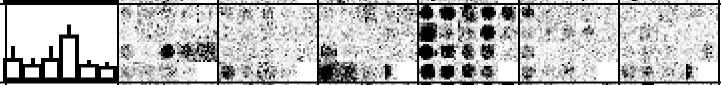							0.97
mwgrat10K#6768	expression: liver kidney; strains: shrsp sprague_dawley wistar_kyoto;	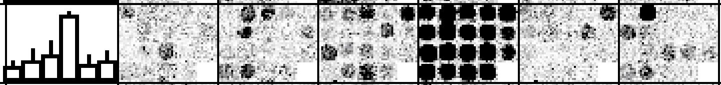							0.97
M33994	cytochrome p-450 iic13 cyp2c13; p-450g; g	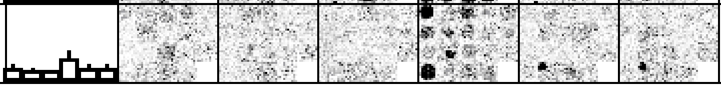							0.97

GeneID	GeneName	Signals	Brain	Heart	Kidney	Liver	Lung	Spleen	OSI
NM_022215	glycerol 3-phosphate dehydrogenase gpd3								0.97
mwgrat10K# 6769	expression: liver; strains: wistar_kyoto; similar to								0.97
NM_012741	k-kininogen, differential splicing leads to hmw kngk kngk; kininogen;								0.97
NM_012803	precursor protein c; proc								0.97
mwgrat10K# 6760	expression: liver brain; strains: shrsp sprague_dawley wistar_kyoto;								0.96
AB052846	sterol c5-desaturase c5d								0.96
mwgrat10K# 7354	expression: liver; strains: shrsp sprague_dawley wistar_kyoto;								0.96
AJ001641	brain-1 brn-1 protein brn-1								0.96
mwgrat10K# 7482	expression: liver; strains: sprague_dawley wistar_kyoto;								0.96
NM_053445	delta-5 fatty acid desaturase d5d; 1 fads1								0.96
mwgrat10K# 8722	expression: liver; strains: wistar_kyoto; similar to								0.96
mwgrat10K# 9539	expression: heart; strains: shrsp; similar to gbp|m93275|m93275_1								0.96
AF329091	e-3 epididymal fluid protein; 2d6 glycoprotein								0.96
mwgrat10K# 6794	expression: liver; strains: shrsp wistar_kyoto; similar to								0.96
NM_017251	gap junction membrane channel protein beta 1 gjb1; aa 1-283								0.96
mwgrat10K# 8369	expression: liver; strains: shrsp; similar to gbp|ak012480|ak012480_1								0.96
U78123	zinc finger protein 12 azf12								0.96
mwgrat10K# 9338	expression: brain; strains: wistar_kyoto; similar to								0.96
mwgrat10K# 7375	expression: liver; strains: shrsp sprague_dawley wistar_kyoto;								0.96
NM_013198	monoamine oxidase b maobf3; maob								0.96
mwgrat10K# 7381	expression: liver kidney; strains: shrsp wistar_kyoto; similar to								0.96
S83436	glutahione s-transferase subunit 13 rgstk1-1								0.96
M12021	myosin light chain mlc1-f								0.96

GeneID	GeneName	Signals	Brain	Heart	Kidney	Liver	Lung	Spleen	OSI
NM_017094_1	growth hormone receptor/binding protein ghr/bp; receptor precursor;								0.96
NM_030996_1	opioid receptor, sigma 1 oprs1; brain receptor; sigma1								0.95
AF072439	zinc-finger protein-37								0.95
NM_057137	phenylalkylamine ca2 antagonist emopamil binding protein ebp; sterol								0.95
NM_012901_1	polyprotein 1-microglobulin/bikunin; alpha-1 microglobulin/bikunin ambp								0.95
NM_019363	aldehyde oxidase female form aox1; liver								0.95
NM_021847	vamp-associated protein b vap-b; vapb								0.95
U33500	retinol dehydrogenase type ii								0.95
X68692	glucagon hepatic receptor related to the receptor for the glucagon-like								0.95
L78306	hydroxyindole-o-methyltransferase								0.94
mwgrat10K#6766	expression: liver; strains: shrsp; similar to pir nf00514528 brain cdna,								0.94
U95157	ryanodine receptor type ii ratryr2								0.94
mwgrat10K#7390	expression: liver; strains: sprague_dawley wistar_kyoto;								0.94
D90109	long-chain acyl-coa synthetase								0.94
L34821	succinate semialdehyde dehydrogenase ssadh								0.94
D38069	udp glucuronosyltransferase ugt1								0.94
mwgrat10K#8688	expression: liver; strains: wistar_kyoto; similar to								0.93
mwgrat10K#7388	expression: liver brain; strains: shrsp sprague_dawley wistar_kyoto;								0.93
NM_017294	protein kinase c and casein kinase substrate in neurons 1 pacsin1;								0.93
NM_012701	adrenergic receptor beta 1 adrb1; beta-1; beta-1-adrenergic								0.93
S57790	3 alpha-hydroxysteroid dehydrogenase 3								0.93
S78744	protein s								0.93
mwgrat10K#6844	expression: liver heart; strains: shrsp sprague_dawley wistar_kyoto;								0.93

GeneID	GeneName	Signals	Brain	Heart	Kidney	Liver	Lung	Spleen	OSI
NM_012531	catecholamine-o-methyltransferase comt; catechol-o-methyltransferase	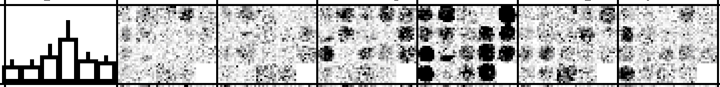							0.92
NM_019298	cholinergic receptor, nicotinic, delta polypeptide chrnd; acetylcholine	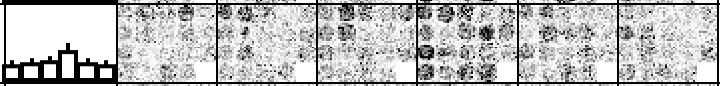							0.92
AF178669	p34	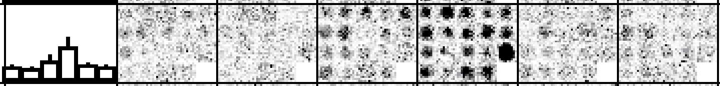							0.92
J05499	l-glutamine amidohydrolase	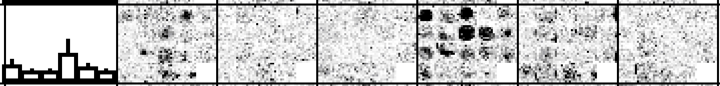							0.90
mwgrat10K#7477	expression: liver brain kidney; strains: shrsp sprague_dawley	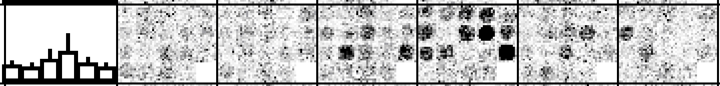							0.90
NM_017123	schwannoma-derived growth factor precursor sdgf; amphiregulin areg	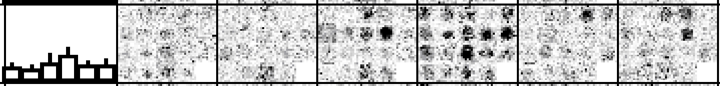							0.90
S58528	integrin alpha v subunit	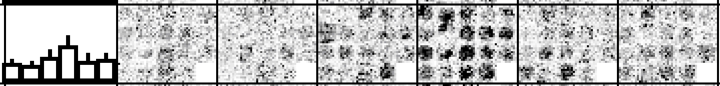							0.90
X06769	c-fos protein aa 1-380	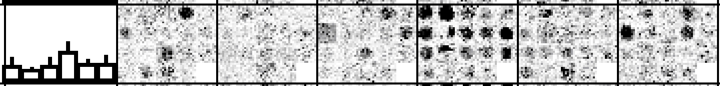							0.90
NM_012883	estrogen sulfotransferase; ste	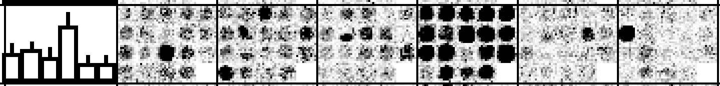							0.90
L26267	nuclear factor kappa b	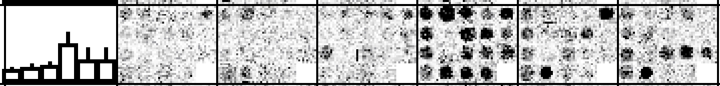							0.89
X81448	keratin 18	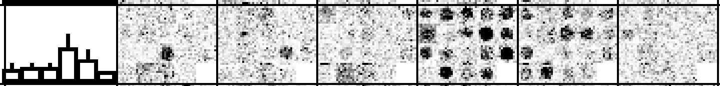							0.89
L00094	angiotensinogen pat	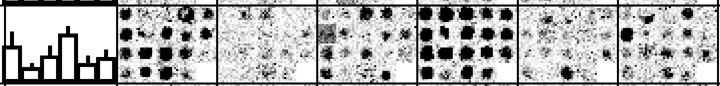							0.89
NM_031664_1	solute carrier family 28; sodium-coupled nucleoside	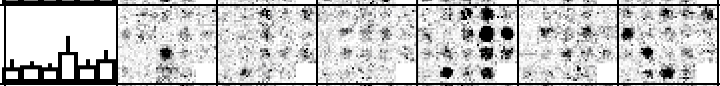							0.88
NM_022179	hexokinase type iii; 3 hk3	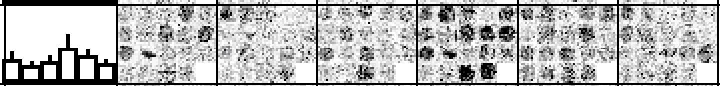							0.88
NM_012594	reading frame lactalbumin protein sequence is in conflict with the	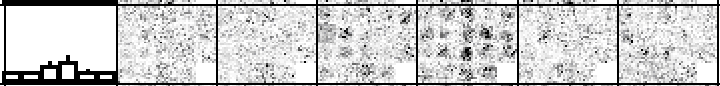							0.88
mwgrat10K#6852	expression: liver; strains: shrsp wistar_kyoto; similar to	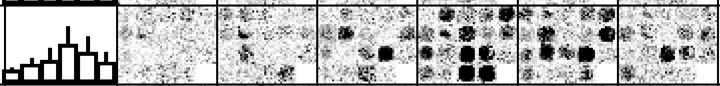							0.88
NM_057133	nuclear receptor subfamily 0, group b, member 2 nr0b2; small	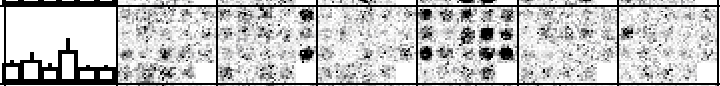							0.88
J02844	carnitine octanoyltransferase	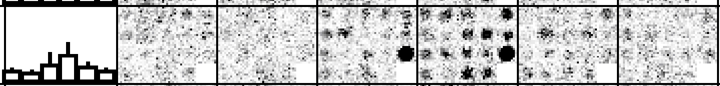							0.87
NM_022228	udp-glucuronosyltransferase 2a1 precursor ugt2a1p; ugt2a1	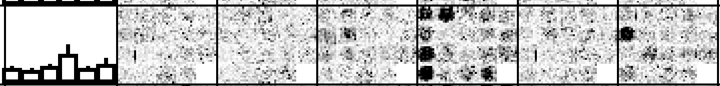							0.87
NM_052809	cytosolic cysteine dioxygenase 1 cdo1; ec 1.13.11.20	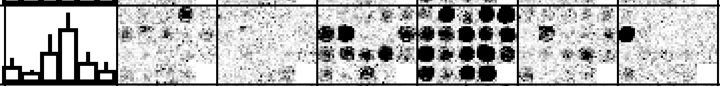							0.87
NM_031587	peroxisomal membrane protein 2, 22 kda pxmp2; pmp22	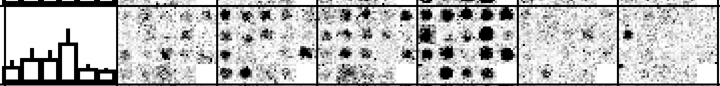							0.86
mwgrat10K#9628	expression: kidney; strains: shrsp; similar to gbp|bc012664|bc012664_1	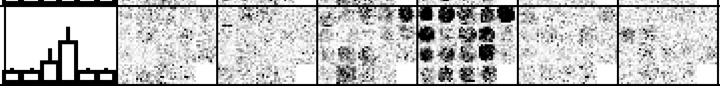							0.86
mwgrat10K#8127	expression: liver; strains: shrsp; similar to pir nf00524545 pals2-beta	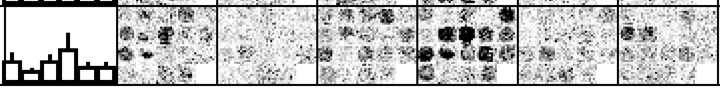							0.86

GeneID	GeneName	Signals	Brain	Heart	Kidney	Liver	Lung	Spleen	OSI
mwgrat10K# 6764	expression: liver kidney; strains: shrsp wistar_kyoto; similar to	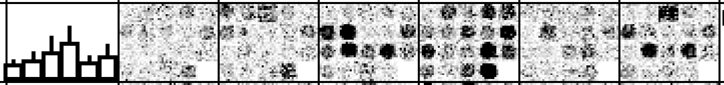							0.86
AJ245707	2-hydroxyphytanoyl-coa lyase	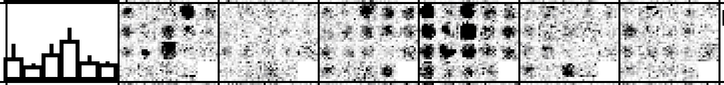							0.86
U01914	akap95	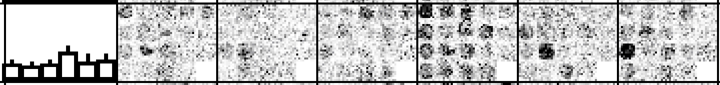							0.86
NM_031705	dihydropyrimidinase dpys	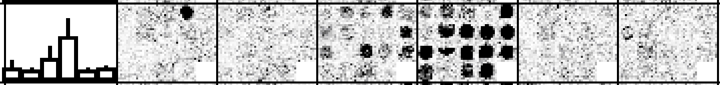							0.86
mwgrat10K# 9636	expression: kidney; strains: shrsp; similar to gbp|bc012664|bc012664_1	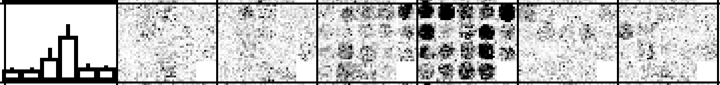							0.85
NM_017256	transforming growth factor, beta receptor iii tgfbr3; betaglycan; type	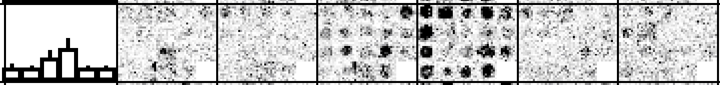							0.85
NM_031760	atp-binding cassette, sub-family b mdr/tap, member 11 abcb11;	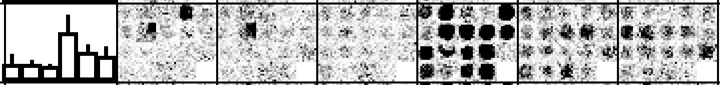							0.85
mwgrat10K# 9149	expression: liver; strains: wistar_kyoto; similar to pir	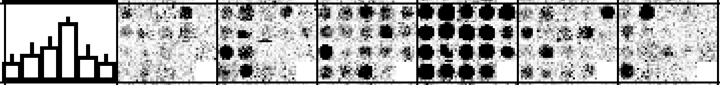							0.84
NM_017303	potassium voltage gated channel, shaker related subfamily, beta	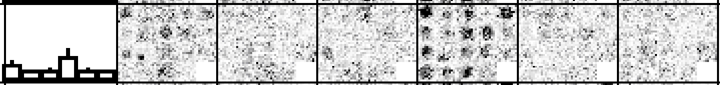							0.84
mwgrat10K# 8022	expression: liver brain; strains: shrsp; similar to pir nf00529385	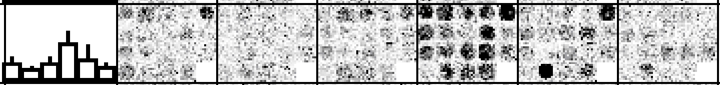							0.84
mwgrat10K# 8410	expression: liver; strains: shrsp; similar to pir nf00519163 aldo-keto	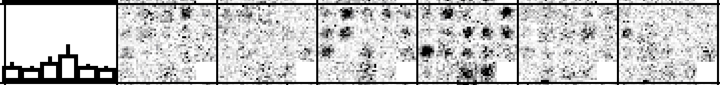							0.83
mwgrat10K# 8692	expression: liver; strains: wistar_kyoto; similar to	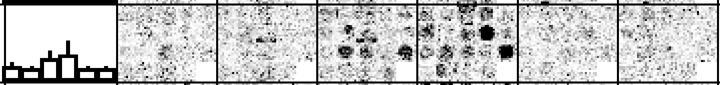							0.82
mwgrat10K# 8413	expression: liver; strains: shrsp; similar to gbp|m57729|m57729_1 c5	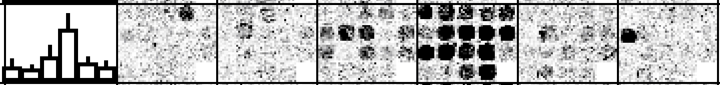							0.81
mwgrat10K# 8377	expression: liver; strains: shrsp; similar to gbp|s47225|s47225_1	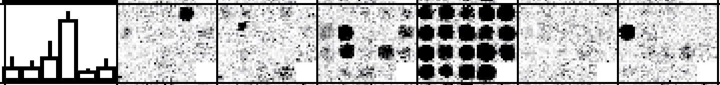							0.81
NM_053567	formiminotransferase cyclodeaminase ftcd;	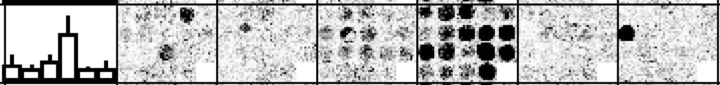							0.80
NM_012516	complement component 4 binding protein, alpha c4bpa; bovine c4bp	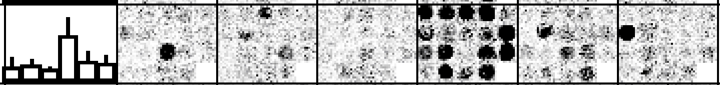							0.79
X94769	choline dehydrogenase rrchdh	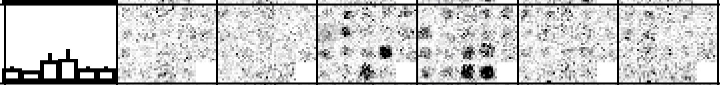							0.79
NM_012568	glycine receptor alpha 2 subunit glycine receptor, neonatal glra2;	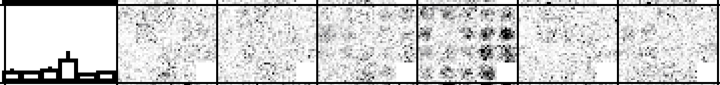							0.78
NM_012582 _1	expression: liver strains: sprague_dawley	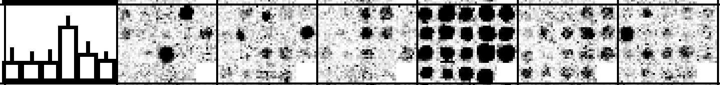							0.78
NM_053902	kynureninase l-kynurenine hydrolase kynu	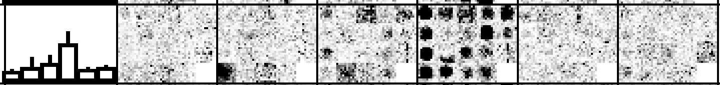							0.78
NM_053853	n-acetyltransferase 1 arylamine n-acetyltransferase nat1;	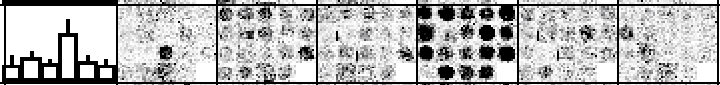							0.78
mwgrat10K# 9662	expression: liver; strains: shrsp; similar to gbp|ab059428|ab059428_1	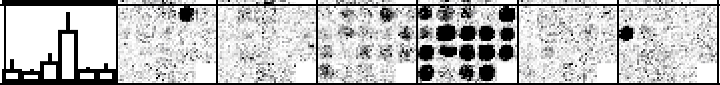							0.77
X52028	cytochrome p450 iid3 protein p450 iid3; cyp2d3; p-450	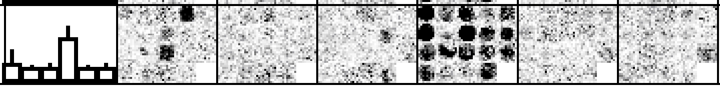							0.76

GeneID	GeneName	Signals	Brain	Heart	Kidney	Liver	Lung	Spleen	OSI
NM_012833	atp-binding cassette, sub-family c cftr/mrp, member 2 cmoat;	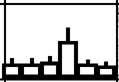	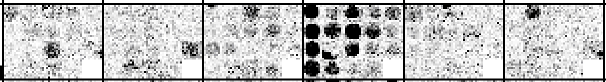						0.75
NM_013120	glucokinase regulatory protein gckr; regulator	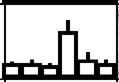	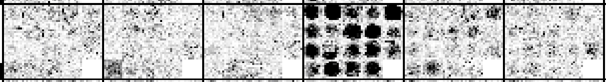						0.74
NM_017225	phosphatidylcholine transfer protein pc-tp; pctp	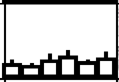	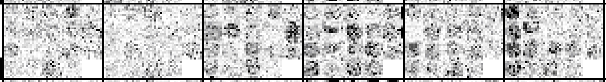						0.73
NM_017024	lecithin-cholesterol acyl transferase aa 1-440; acyltransferase lcat;	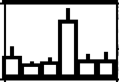	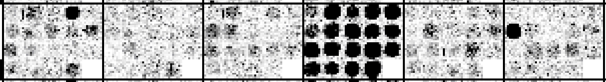						0.72
NM_012871	oxytocin receptor oxtr; otr	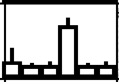	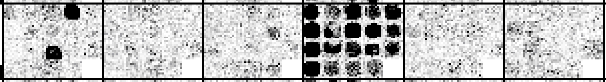						0.72
NM_024352	macrophage stimulating 1 hepatocyte growth factor-like mst1;	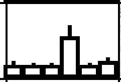	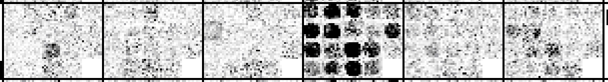						0.72
NM_017143	coagulation factor x f10	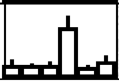	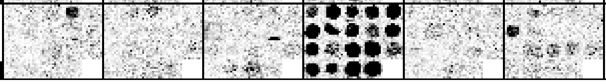						0.70

GeneID	GeneName	Signals	Brain	Heart	Kidney	Liver	Lung	Spleen	OSI
NM_012967	intercellular adhesion molecule 1 icam1; molecule-1	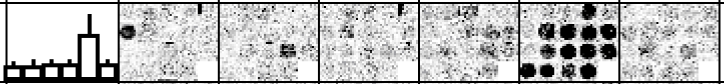							1.00
NM_017232	mitogen inducible cyclooxygenase cyclooxygenase-2;	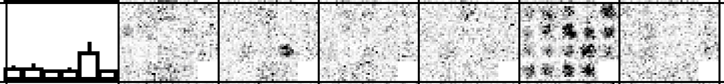							1.00
NM_031764	discoidin domain receptor family, member 2 ddr2; rtk40 homolog	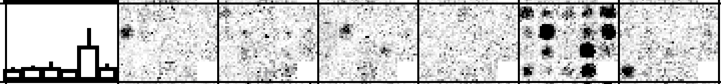							1.00
NM_012521	calcium-binding protein, intestinal, vitamin d-dependent 9-kda cabp	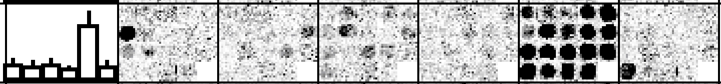							1.00
NM_019358	epithelial cell surface transmembrane protein antigen	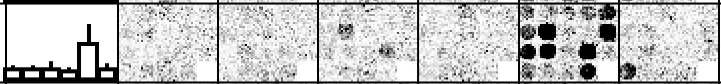							1.00
NM_019231	mitogen activated protein kinase 13 prkm13; mitogen-activated p38delta	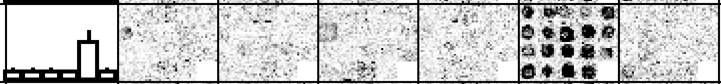							1.00
NM_031055	matrix metalloproteinase 9 gelatinase b, 92-kda type iv	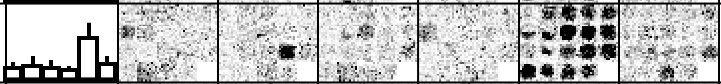							0.99
NM_012613	guanylate cyclase a/atrial natriuretic peptide receptor guanylate cyclase	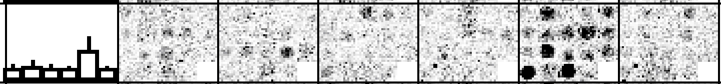							0.99
NM_053380	solute carrier family 34 sodium phosphate, member 2 slc34a2; type	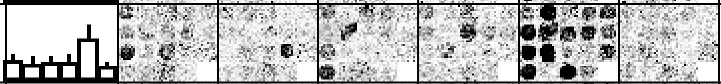							0.99
D88586	ribonuclease 1 precursor r1; 7 r7; eosinophil cationic protein	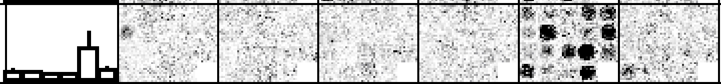							0.99
NM_031684	equilbrative nitrobenzylthioinosine-sensitive	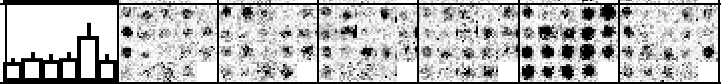							0.99
NM_012761	trans-acting transcription factor 4 sp4; zinc finger protein hf-1b	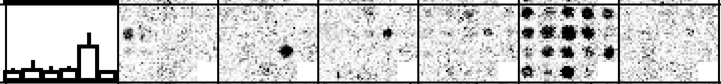							0.99
NM_022687	transcription factor hes-3 hes3	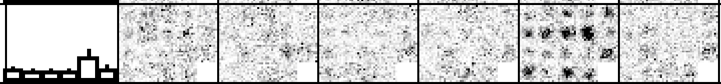							0.99
NM_013187	phospholipase c, gamma 1 plcg1; c	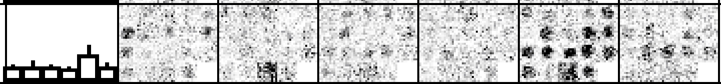							0.99
NM_017329	pulmonary surfactant-associated glycoprotein a sftpa1; prepulmonary	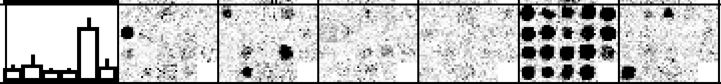							0.99
NM_023983	l-gicerin; mcam	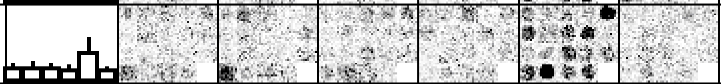							0.99
NM_012878	primary translation product of sp-d sp-d; pulmonary surfactant protein d	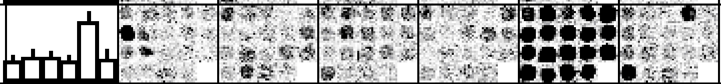							0.99
NM_021654	gap junction membrane channel, protein alpha 4 connexin 37 gja4;	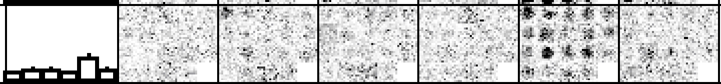							0.99
NM_024142	osteoregulin loc79110	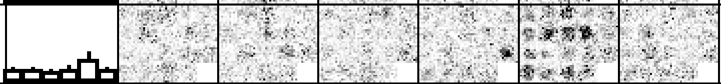							0.99
X16261	myosin heavy chain 21 aa 621; 279	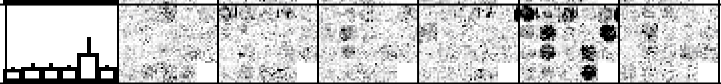							0.99
AF089866	keratin 19 k19	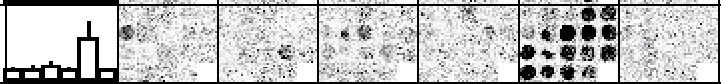							0.99
NM_053952	nucleoporin 155kd nup155	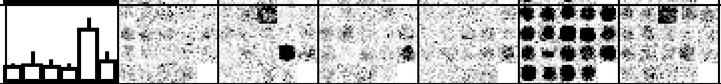							0.99
NM_053895	fgf receptor activating protein frag1 frag1; 1	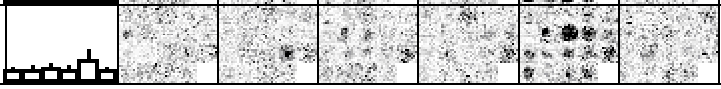							0.99

GeneID	GeneName	Signals	Brain	Heart	Kidney	Liver	Lung	Spleen	OSI
AF346790	melanoma-associated antigen mg50								0.99
AF068861	beta defensin-2								0.99
X13295	alpha-2u protein aa 1-198								0.99
AF155196	mindin precursor								0.99
NM_012605	myosin, light polypeptide 2, alkali ventricular, skeletal, slow myl2;								0.98
mwgrat10K#7685	expression: kidney brain; strains: sprague_dawley wistar_kyoto;								0.98
NM_022282	discs, large drosophila homolog 2 chapsyn-110 dlg2;								0.98
mwgrat10K#6711	expression: liver kidney heart; strains: shrsp sprague_dawley;								0.98
mwgrat10K#6707	expression: liver heart; strains: shrsp sprague_dawley wistar_kyoto;								0.98
Z46614	caveolin								0.98
AF087453	potassium channel kcnq2								0.98
mwgrat10K#6569	expression: heart brain kidney; strains: shrsp wistar_kyoto; similar								0.98
NM_053333	resistin-like molecule alpha; resistin like retnla								0.98
NM_021586	latent tgf-beta binding protein-2 like protein; ltbp-2 ltbp2								0.98
NM_053738	wnt inhibitory factor 1; wif1								0.98
NM_053336	advanced glycosylation end product-specific receptor ager; for								0.98
AJ005424	bmk1/erk5								0.98
M96578	glial voltage-gated sodium channel; scl-11								0.98
NM_030999	corticotropin releasing hormone receptor crhr1;								0.98
NM_031345	glucocorticoid-induced leucine zipper gilz								0.98
mwgrat10K#6710	expression: heart; strains: wistar_kyoto; similar to								0.97
NM_017185	troponin i, skeletal, fast 2 tnni2; i trp1								0.97
NM_017037	peripheral myelin protein pmp22; rat sr13; cd25								0.97

GeneID	GeneName	Signals	Brain	Heart	Kidney	Liver	Lung	Spleen	OSI
D88666	ps-pla1	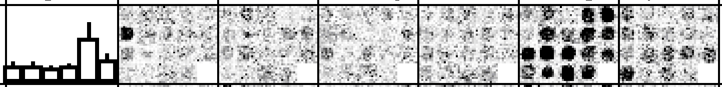							0.97
NM_031836_3	endothelial cell growth factor protein precursor; vascular factor-a188;	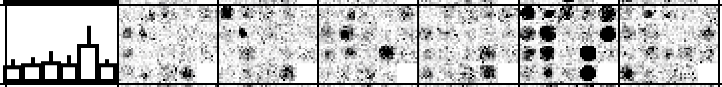							0.97
NM_019143_2	precursor polypeptide aa -32 to 2445; fibronectin; 1 fn1; fn-1	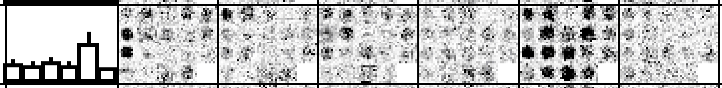							0.97
U28356	cytosolic protein tyrosine phosphatase	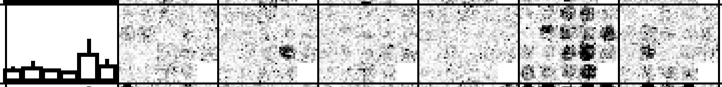							0.97
NM_013051	uteroglobin clara cell secretory protein ugb; pcb binding precursor	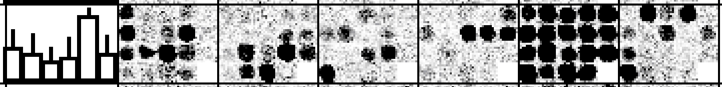							0.97
L19104	heparin-binding fibroblast growth factor receptor 2	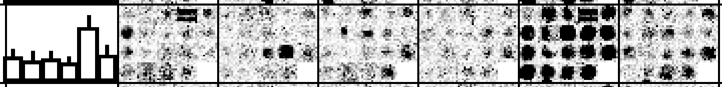							0.97
mwgrat10K#7735	expression: kidney; strains: wistar_kyoto; similar to	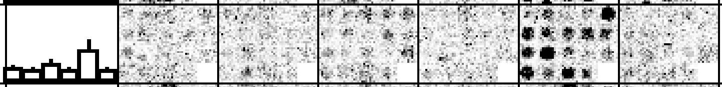							0.96
NM_031642	core promoter element binding protein copeb; zinc finger	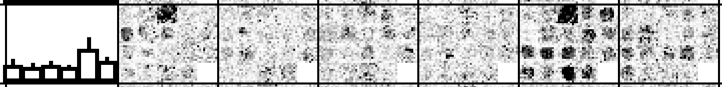							0.96
NM_053565	cytokine inducible sh2-containing protein 3 cish3; suppressor of	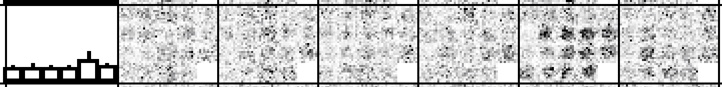							0.96
NM_030858	mad mothers against decapentaplegic, drosophila homolog	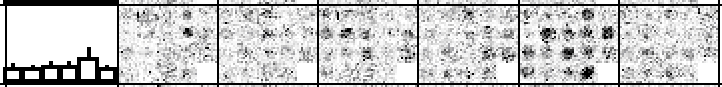							0.96
NM_022264_2	c-kit receptor tyrosine kinase isoform c-kit proto-oncogene; kit	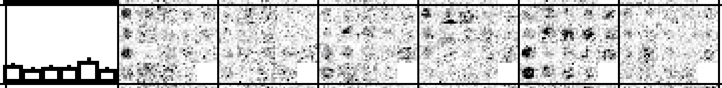							0.96
NM_030829	g protein-coupled receptor kinase 5 gprk5; protein coupled	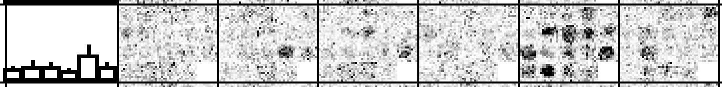							0.96
AF359356	platelet-derived growth factor receptor beta; pdgfrb	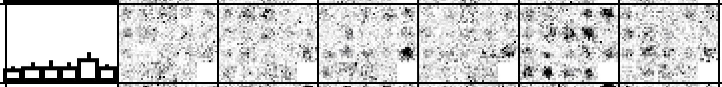							0.96
AF134773	lim protein fhl1	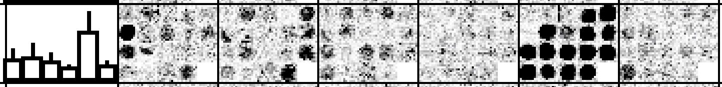							0.96
NM_012827	bone morphogenetic protein 4 bmp4	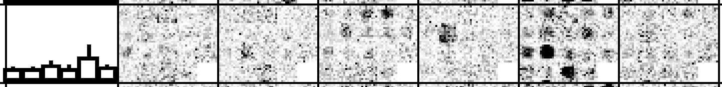							0.96
M29853	cytochrome p-450 isozyme 5	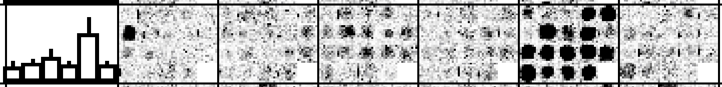							0.96
M15202	troponin t class ic beta-3	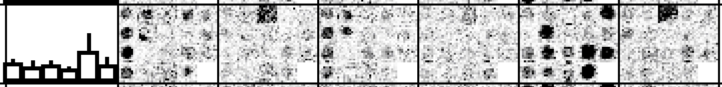							0.96
AF007554	mucin 1 muc1	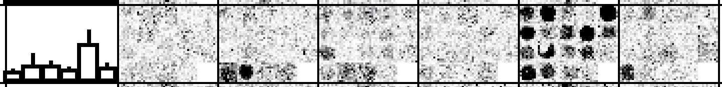							0.96
NM_053920	thyroid hormone receptor interactor 10 trip10; salt-tolerant protein	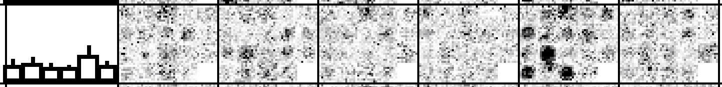							0.95
M88469	f-spondin	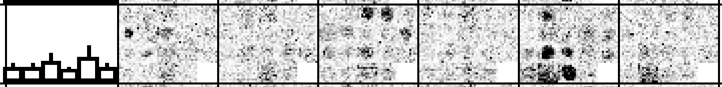							0.95
D89730	t16	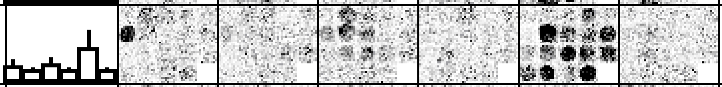							0.95
NM_053508	tektin 1 tekt1	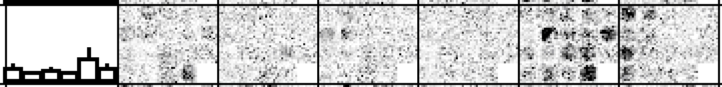							0.95
mwgrat10K#6970	expression: brain; strains: shrsp; similar to gbp|x61452|x61452_1 h5 -	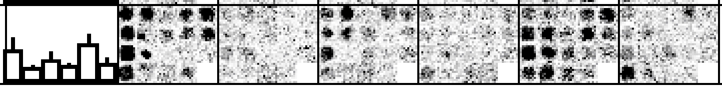							0.95

GeneID	GeneName	Signals	Brain	Heart	Kidney	Liver	Lung	Spleen	OSI
mwgrat10K# 6626	expression: kidney heart; strains: shrsp sprague_dawley wistar_kyoto;								0.95
NM_017090	guanylate cyclase, soluble, alpha 1 gtp pyrophosphate - lyase gucy1a1;								0.95
AF140345	n-methyl-d-aspartate receptor-like subunit								0.95
M18864	bone protein i								0.94
NM_053796	junctional adhesion molecule jam jam; 1 jam1								0.94
AF071003	mink-related peptide 1								0.94
AB059655	adenosine deaminase; ada								0.93
mwgrat10K# 7125	expression: brain kidney; strains: wistar_kyoto; similar to								0.93
AF439779	caveolin-1 beta								0.93
AJ005394	alpha 1 type v collagen								0.93
mwgrat10K# 7122	expression: brain; strains: shrsp wistar_kyoto; similar to pir								0.93
NM_031239	sh3-domain grb2-like 1 loc81922; sh3p8								0.93
NM_017347	extracellular signal-regulated kinase 1b erk1b; 1;								0.93
NM_022441	serine-threonine kinase receptor type i; activin like 1 acvrl1								0.93
NM_031609	neuroblastoma, suppression of tumorigenicity 1 nbl1;								0.92
NM_022693	for sh3-domain binding protein sh3bp4; 4								0.92
NM_053883	dual-specificity protein tyrosine phosphatase rvh6; dual specificity 6								0.92
mwgrat10K# 7874	expression: brain heart; strains: shrsp sprague_dawley; similar to								0.92
D86581	chordin								0.92
NM_022639	neuronal nicotinic acetylcholine receptor subunit alpha10; chrna10								0.91
AF061752	unknown msf-1								0.91
mwgrat10K# 6553	expression: heart brain; strains: shrsp sprague_dawley wistar_kyoto;								0.91
NM_053352	chemokine orphan receptor 1 rdc1; rdc-1 protein								0.91

GeneID	GeneName	Signals	Brain	Heart	Kidney	Liver	Lung	Spleen	OSI
mwgrat10K# 8216	expression: liver brain heart; strains: shrsp sprague_dawley wistar_kyoto;	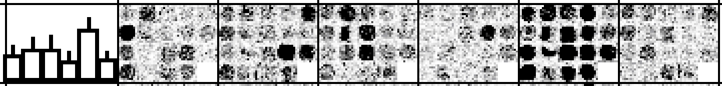							0.91
AB019120	seven transmembrane receptor	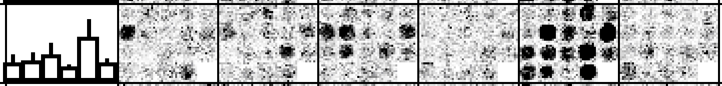							0.91
AF241260	claudin-5	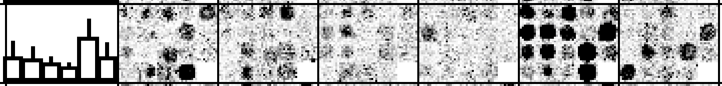							0.91
AF037272	wap four-disulfide core domain protein ps20	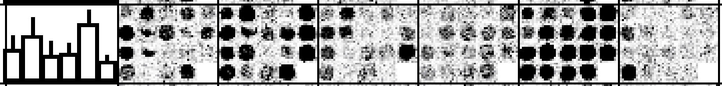							0.91
NM_053596	endothelin-converting enzyme 1 ece1	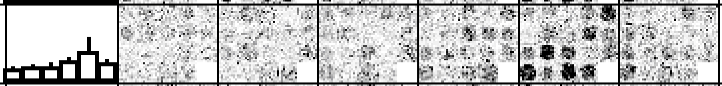							0.91
NM_053713	kruppel-like factor 4; gut; klf4	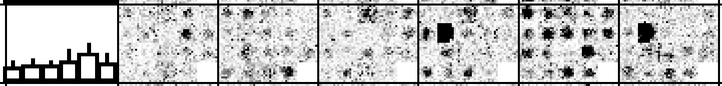							0.90
X58830	vgr	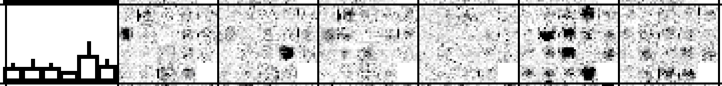							0.90
NM_022523	platelet endothelial tetraspan antigen-3 cd151	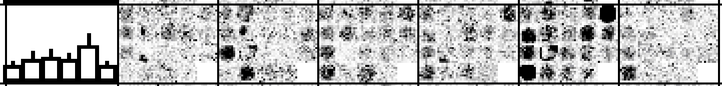							0.90
NM_031321	slit drosophila homolog 3 slit3; megf5	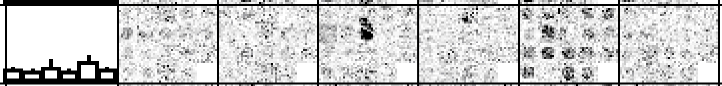							0.90
M27224	t-cell receptor c-beta-0, exon x 5' and 3' ends put. putative	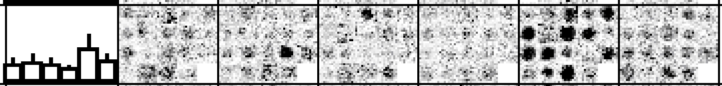							0.90
NM_019310	cxc chemokine receptor1-like protein cxcr1; interleukin 8 receptor, alpha	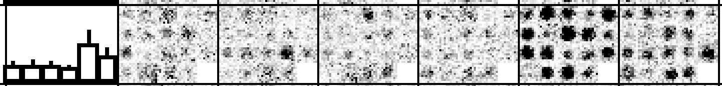							0.90
NM_019243	prostaglandin f2a receptor regulatory protein precursor; f2 negative	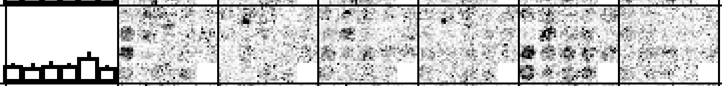							0.90
NM_053302	putative g-protein coupled receptor g10d; adrenomedullin admr	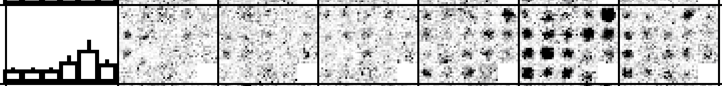							0.90
mwgrat10K# 6329	expression: heart; strains: shrsp sprague_dawley wistar_kyoto;	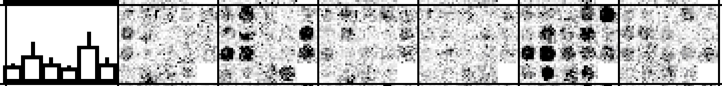							0.90
NM_031825	fibrillin-1 fbn1	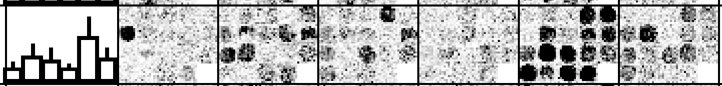							0.90
AF439778	caveolin-1 alpha	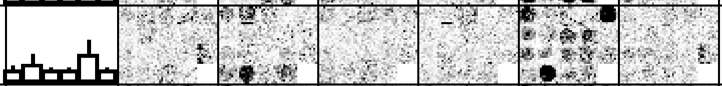							0.90
mwgrat10K# 8999	expression: brain; strains: sprague_dawley; similar to	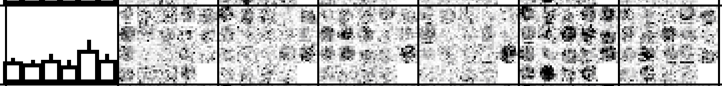							0.89
mwgrat10K# 6568	expression: liver heart kidney brain; strains: shrsp sprague_dawley	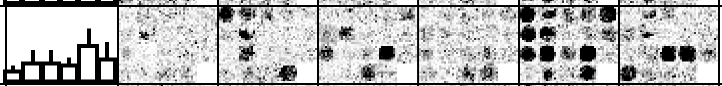							0.89
NM_013034	solute carrier family 6 neurotransmitter transporter,	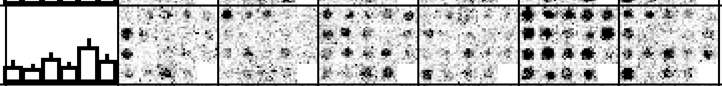							0.89
M37569	homeobox protein	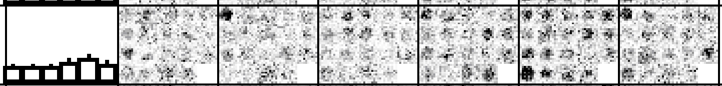							0.89
M64780	agrin	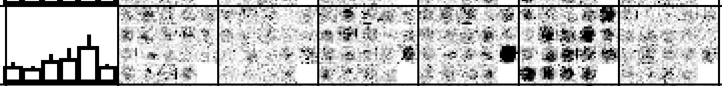							0.89
mwgrat10K# 6641	expression: heart brain; strains: sprague_dawley wistar_kyoto;	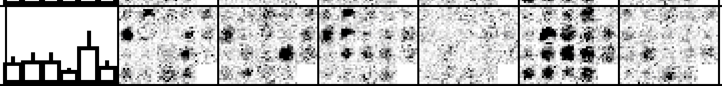							0.88
NM_031585	phospholipase a2, group ib, pancreas pla2g1b; pancreatic a-2	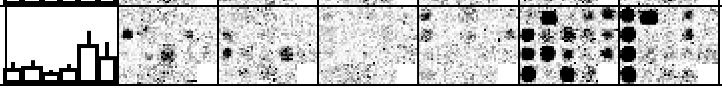							0.88

GeneID	GeneName	Signals	Brain	Heart	Kidney	Liver	Lung	Spleen	OSI
NM_019386	tissue-type transglutaminase tgaseii; tgm2								0.88
NM_031528	retinoic acid receptor alpha 2 isoform rar; receptor, rara								0.88
NM_013026	syndecan syndecan, hspg core protein; 1 synd1								0.88
mwgrat10K#8767	expression: kidney; strains: wistar_kyoto; similar to pir								0.88
NM_031830	reggie1-1 flot2; reggie1-2; reggie1-4; r-reggie-1.1								0.87
NM_030987	guanine nucleotide-binding protein beta 1 gnb1; g subunit; beta1 rgb1								0.87
mwgrat10K#9349	expression: brain; strains: shrsp; similar to pir nf00130893 lim domain								0.87
NM_031318	t-complex testis expressed 1 tctex1; tctex-1; protein								0.87
NM_031809_1	cyclic nucleotide-gated channel beta subunit 1 cngb1; cng4.1								0.87
NM_013104	insulin-like growth factor binding protein 6 igfbp6; igfbp-6 rbp-6								0.85
Y13275	d6.1a protein								0.85
NM_053591	dipeptidase 1 dpep1								0.85
AF324255	global ischemia-inducible protein 11								0.85
NM_031658	mesothelin msln; erc								0.84
mwgrat10K#7841	expression: brain heart; strains: shrsp sprague_dawley; similar to pir								0.84
mwgrat10K#8458	expression: brain; strains: wistar_kyoto; similar to								0.83
NM_031776	guanine aminohydrolase gah; deaminase gda								0.83
NM_053816_1	calcitonin receptor calcr; c1b								0.83
NM_012997_1	purinergic receptor p2x, ligand-gated ion channel, 1 p2rx1; p2x1 isoform;								0.83
NM_031042	general transcription factor iif, polypeptide 2 30kd subunit gtf2f2;								0.82
NM_024138	guanine nucleotide binding protein g protein, gamma 7 subunit gng7;								0.82
NM_012499	adenomatosis polyposis coli apc; protein								0.82
U42581	p85								0.81

GeneID	GeneName	Signals	Brain	Heart	Kidney	Liver	Lung	Spleen	OSI
NM_019371	factor-responsive smooth muscle protein sm-20	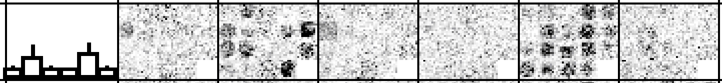							0.81
NM_053634	ficolin b fcnb; ficolin-b	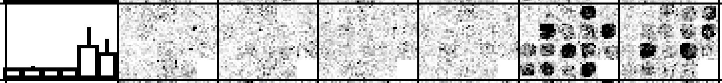							0.81
NM_012797	inhibitor of dna binding 1, helix-loop-helix protein splice	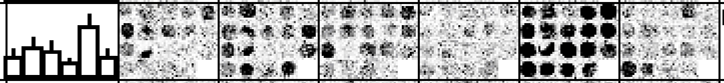							0.80
U61261	laminin-5 alpha 3 chain	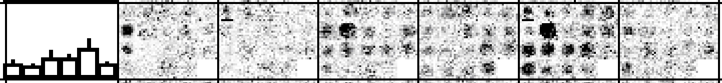							0.80
U77697	platelet-endothelial cell adhesion molecule-1/cd31	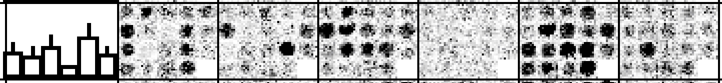							0.78
X81449	keratin 19	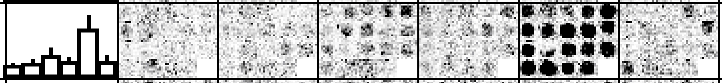							0.77
mwgrat10K#9097	expression: heart; strains: sprague_dawley; similar to	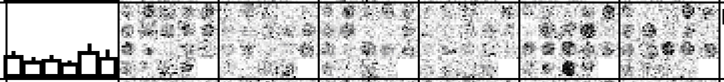							0.76
mwgrat10K#7089	expression: kidney brain; strains: shrsp sprague_dawley; similar to pir	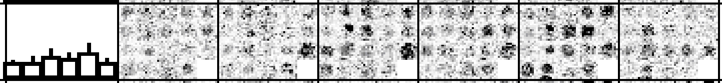							0.76
NM_031116	small inducible cytokine a5 rantes scya5	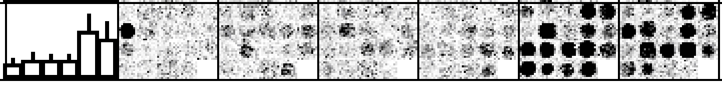							0.75

GeneID	GeneName	Signals	Brain	Heart	Kidney	Liver	Lung	Spleen	OSI
AJ302650	rp59 protein rp59								1.00
NM_012635	trypsinogen 1 is 3rd base in codon; i trp1; ii; pancreatic trypsin prss1;								0.99
NM_031691	integrin alpha x cd11c itgax; d								0.99
M17092	this cds feature is included to show the translation of the corresponding								0.99
NM_023022	50 kd glycoprotein rh50; 50kd rhag								0.99
NM_022505	rh blood group protein; rhesus								0.99
NM_012791	dual-specificity tyrosine- y -phosphorylation regulated kinase 1a								0.99
NM_031348	ficolin a fcna; ficolin-a								0.99
mwgrat10K#6881	expression: liver brain; strains: shrsp sprague_dawley wistar_kyoto;								0.99
NM_024158	deoxycytidine kinase dck								0.98
M15402	immunoglobulin kappa-chain vj precursor								0.98
AF286344	immunoglobulin 4g6 light chain variable region								0.98
NM_031644	prostaglandin d2 synthase 2, hematopoietic ptgds2;								0.98
AF111111	sperm tail protein spag5								0.98
NM_023987	inhibitor of apoptosis protein 1 birc2								0.98
L07406	immunoglobulin kappa-chain igkv								0.98
NM_057209	myosin light chain kinase 2, skeletal muscle mylk2; e.c. 2.7.1.37								0.98
NM_019186	adp-ribosylation-like 4 arl4; arl								0.96
NM_019295	lymphocyte antigen cd5 cd5; glycoprotein								0.96
AB004831	b29/ig-beta/cd79b b29/ig-beta								0.96
X64589	cyclin b								0.96
M22520	igl								0.96
U94913	h-k-atpase alpha 2a subunit hkalpha2a; h,k+atpase; 2b								0.95

GeneID	GeneName	Signals	Brain	Heart	Kidney	Liver	Lung	Spleen	OSI
U17565	intestinal dna replication protein								0.95
X68782	immunoglobulin heavy chain vdjh2 region								0.95
M87786	immunoglobulin light chain								0.95
mwgrat10K# 9129	expression: liver; strains: sprague_dawley; similar to								0.95
L07408	immunoglobulin kappa light chain variable region; kappa-chain igkv								0.94
NM_024485	cholinergic receptor, nicotinic, alpha polypeptide 1 muscle chrna1;								0.94
X62328	t-cell receptor v alpha j alpha rvalphat48a3								0.94
X56190	ecl								0.93
NM_053372	secretory leukocyte protease inhibitor slpi								0.93
NM_019296	cell division cycle control protein 2 cdc2a; cdc2								0.93
L07399	immunoglobulin heavy chain ighv@								0.93
D16237	cdc25b								0.93
M34238	ccaat binding transcription factor-b subunit cbf-b								0.93
NM_017250	5-hydroxytryptamine serotonin receptor 2b htr2b; stomach fundus								0.93
M13800	immunoglobulin mu-chain igm; mu-immunoglobulin								0.92
X60370	microtubule associated protein map; neuraxin aa 1-863								0.92
AF091111	nectin-1								0.91
X62323_1	pan-2; pan-1								0.91
J00741	immunoglobulin delta heavy chain constant region								0.91
M15254	platelet factor 4 precursor								0.91
NM_031657	g protein-coupled receptor kinase 6, splice variant c grk6; 6 gprk6; grk6b								0.90
mwgrat10K# 7342	expression: liver brain; strains: shrsp wistar_kyoto; similar to								0.90
X00336	ifn								0.90

GeneID	GeneName	Signals	Brain	Heart	Kidney	Liver	Lung	Spleen	OSI
AB026903_1	decay accelerating factor soluble-form precursor daf;								0.89
NM_053572	mt-protocadherin; kiaa1775								0.89
J04636	nicotinic acetylcholine receptor beta-3 subunit precursor								0.89
NM_019179	thymidylate synthase tyms								0.89
NM_012603	avian myelocytomatosis viral v-myc oncogene homolog myc; c-myc								0.89
NM_012968	interleukin 1 receptor accessory protein il1rap; interleukin-1 il-1								0.88
NM_012969	insulin receptor substrate 1 irs1; irs-1								0.87
NM_017187	high mobility group protein 2 hmg2								0.87
U22893	yb2								0.87
NM_012637	protein-tyrosine phosphatase ptpn1; protein-tyrosine-phospatase								0.86
NM_013195	interleukin 2 receptor beta-chain p70/75; receptor, il2rb								0.85
NM_031659	protein-glutamine gamma-glutamyltransferase;								0.85
NM_013165_1	cholecystokinin b receptor cckbr; cck -r deltatm3-e 3								0.84
AF394785	cell adhesion regulator car1; ferroportin 1								0.84
NM_017168	phospholipase c type iv plp iv; c, gamma 2 plcg2								0.84
mwgrat10K#7570	expression: heart kidney brain; strains: shrsp sprague_dawley								0.84
NM_053719	embigin protein embigin; emb								0.83
mwgrat10K#9261	expression: brain; strains: sprague_dawley; similar to pir								0.83
X07189	immunoglobulin g heavy chain								0.83
NM_030855	dna ligase i lig1								0.82
NM_080583_1	adaptor-related protein complex 2, beta 1 subunit ap2b1; beta-chain								0.82
NM_053918	pituitary hormone alpha subunit pituitary hormone; glycoprotein								0.82
NM_013075	homeobox-plus hoxa1 protein hoxa1; homeo box a1								0.82

GeneID	GeneName	Signals	Brain	Heart	Kidney	Liver	Lung	Spleen	OSI
NM_013185	hemopoietic cell tyrosine kinase hck; protein-tyrosine	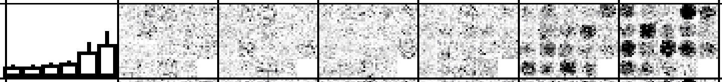							0.79
S79711	cd3 gamma-chain	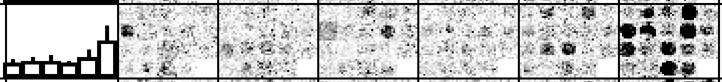							0.79
NM_053688	cone-like cgmp-phosphodiesterase 6 gamma subunit cgmp-pde6 gamma;	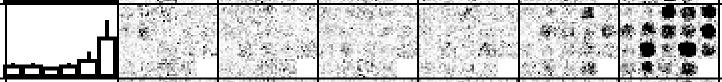							0.78
NM_053983	cd52 antigen cd52; rb7	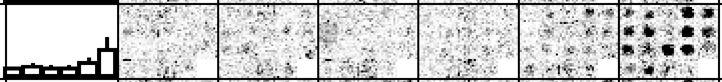							0.77
NM_022244	nadrin loc63994; e2	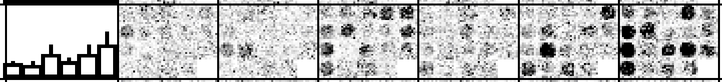							0.75
NM_032063	delta drosophila -like 1 dll1; delta1	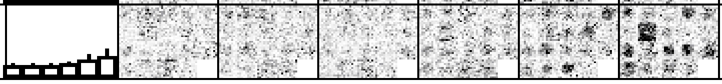							0.72
