# Supplementary material for: Identification of rat lung – prominent genes by a parallel DNA microarray hybridization
Source: BMC Genomics. 2006 Mar 13;7:47. doi: 10.1186/1471-2164-7-47 (PMC1523215; doi:10.1186/1471-2164-7-47)
Supplement: Additional File 3 — Supplementary Table E2B, Two organ-prominent genes in PDF format with spot images. [file 1471-2164-7-47-S3.pdf]

Meaning of columns:

Gene ID: Genbank accession number;

GeneName: name and common symbol of a gene;

Signals: mean scaled fluorescence intensity of brain, heart, kidney, lung, and spleen;

The error bars were based on standard deviation.

OSI: Organ specific index (see text for detail)

Contents

Organs	Page
Heart and brain	1
Heart and kidney	2
Heart and liver	2
Kidney and brain	3
Kidney and liver	4
Liver and brain	11
Liver and spleen	11
Lung and brain	12
Lung and heart	13
Lung and kidney	14
Lung and liver	19
Lung and spleen	21

GeneID	GeneName	Signals	Brain	Heart	Kidney	Liver	Lung	Spleen	OSI
NM_053297	pyruvate kinase 3 pkm2; m1 pk	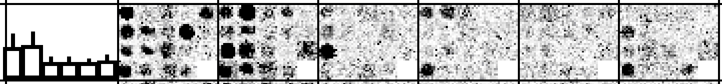							0.99
NM_017049	solute carrier family 4, member 3, anion exchange protein 3 slc4a3;	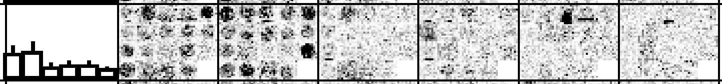							0.97
mwgrat10K# 7159	expression: brain heart; strains: shrsp sprague_dawley wistar_kyoto;	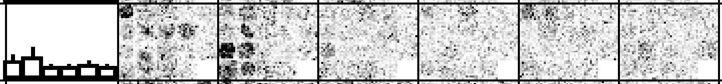							0.97
mwgrat10K# 6205	expression: heart brain; strains: shrsp wistar_kyoto; similar to pir	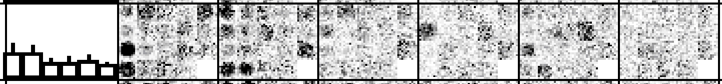							0.96
L19181	receptor-linked protein tyrosine phosphatase	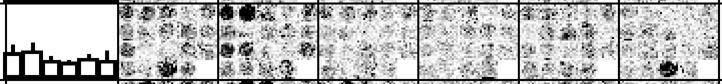							0.96
D16443	rep3b	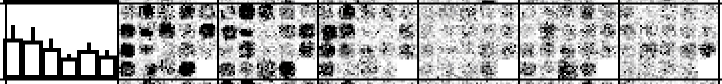							0.87
NM_080690	cask-interacting protein 1; caskin1	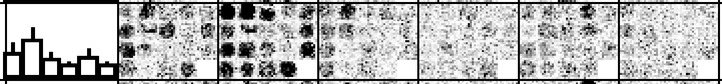							0.86
mwgrat10K# 9078	expression: brain heart; strains: shrsp sprague_dawley wistar_kyoto;	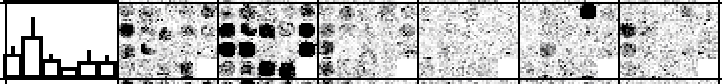							0.81
NM_012660	statin-related protein s1; statin-like stnl	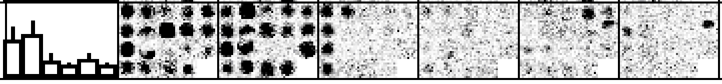							0.73

GeneID	GeneName	Signals	Brain	Heart	Kidney	Liver	Lung	Spleen	OSI
M28654	myosin heavy chain myh	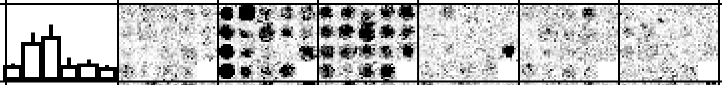							0.99
mwgrat10K# 6735	expression: kidney heart; strains: shrsp sprague_dawley wistar_kyoto;	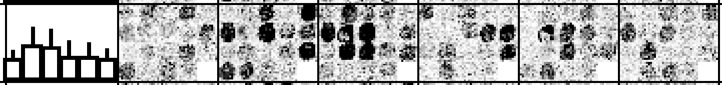							0.98
mwgrat10K# 6486	expression: heart; strains: wistar_kyoto; similar to	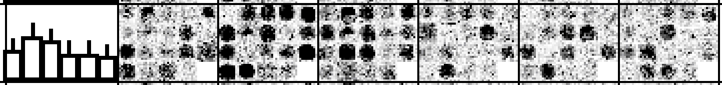							0.97
mwgrat10K# 8630	expression: heart; strains: wistar_kyoto; similar to	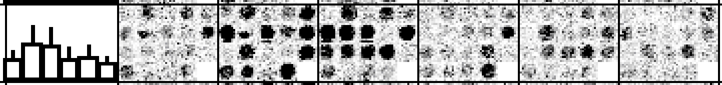							0.96
mwgrat10K# 6723	expression: kidney; strains: wistar_kyoto; similar to	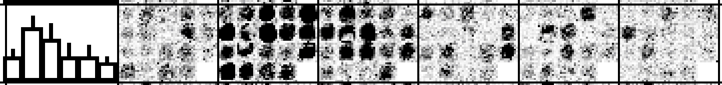							0.95
mwgrat10K# 9150	expression: brain; strains: shrsp; similar to gbp|ak002501|ak002501_1	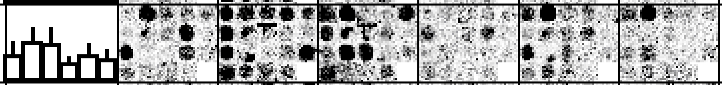							0.94
mwgrat10K# 6166	expression: brain heart; strains: shrsp sprague_dawley wistar_kyoto;	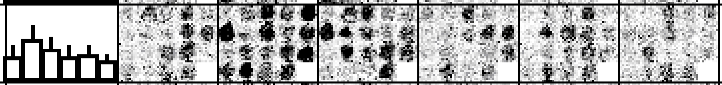							0.91
mwgrat10K# 8145	expression: heart; strains: sprague_dawley wistar_kyoto;	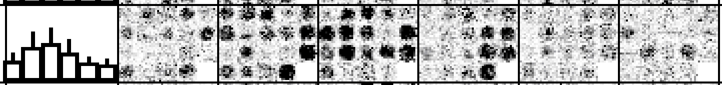							0.88
NM_017163	renal osmotic stress-induced na-cl organic solute cotransporter; x	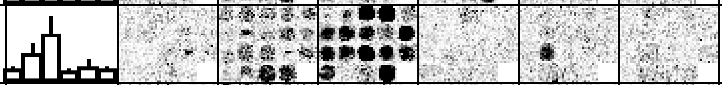							0.88
AF144090	fatty acid-binding protein fabp	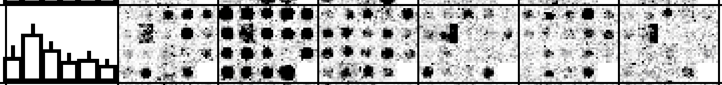							0.87
mwgrat10K# 8124	expression: kidney; strains: shrsp; similar to pir nf00533132 rst - mus	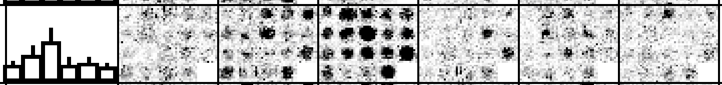							0.87
NM_024162	heart fatty acid binding protein; fabp3	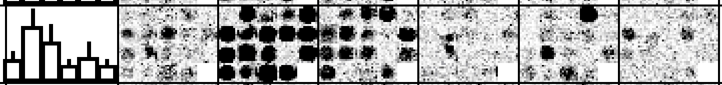							0.81
mwgrat10K# 8080	expression: heart kidney; strains: shrsp wistar_kyoto; similar to	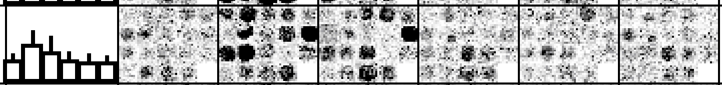							0.80
mwgrat10K# 8148	expression: liver heart; strains: sprague_dawley; similar to pir	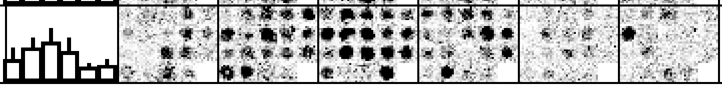							0.71

GeneID	GeneName	Signals	Brain	Heart	Kidney	Liver	Lung	Spleen	OSI
S79214	type x collagen alpha 1 chain	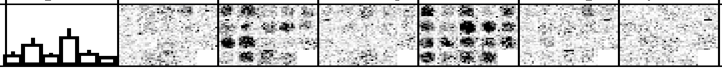							0.83

GeneID	GeneName	Signals	Brain	Heart	Kidney	Liver	Lung	Spleen	OSI
NM_017066_1	pleiotrophin heparine binding factor, hbnf, in the mouse ptn;	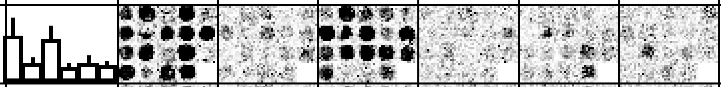							0.99
NM_024396	atp-binding cassette, sub-family a abc1, member 2 abca2; abc2	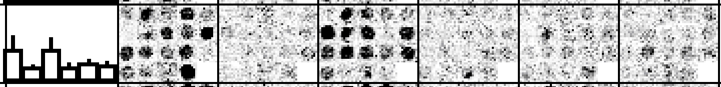							0.98
mwgrat10K#7519	expression: kidney; strains: wistar_kyoto; similar to pir	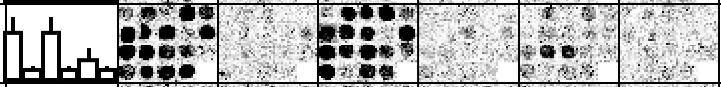							0.98
mwgrat10K#6960	expression: kidney brain; strains: shrsp sprague_dawley wistar_kyoto;	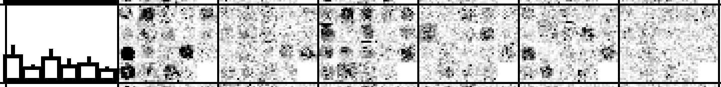							0.94
M64378	olfactory protein; odorant receptor	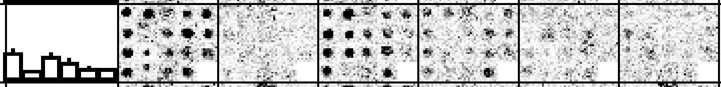							0.93
NM_031984	cerebellar ca-binding protein, spot 35 protein calb1; calbindin-d28k;	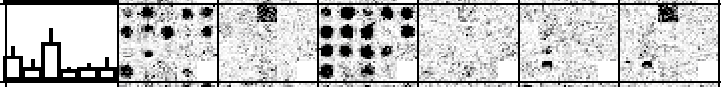							0.89
NM_017212	microtubule-associated protein tau mapt; big; microtubule associated	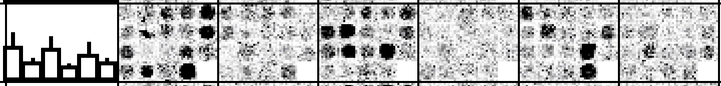							0.88
NM_022676	protein phosphatase 1, regulatory inhibitor subunit 1a ppp1r1a;	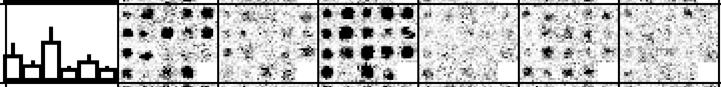							0.87
mwgrat10K#7781	expression: brain; strains: shrsp sprague_dawley; similar to pir	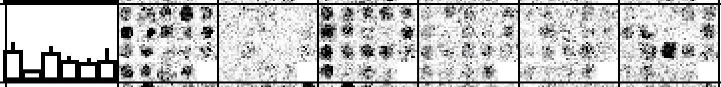							0.86
mwgrat10K#8136	expression: heart brain kidney; strains: shrsp sprague_dawley	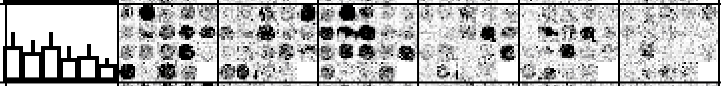							0.85
NM_030997	nerve growth factor-inducible protein vgf; factor inducible	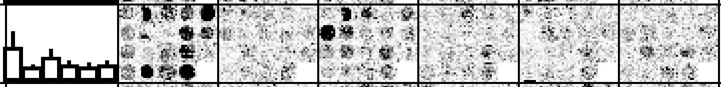							0.84
NM_022533	plasmolipin z49858	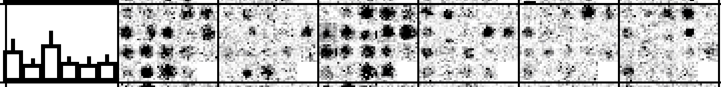							0.83
mwgrat10K#8978	expression: brain; strains: sprague_dawley; similar to pir	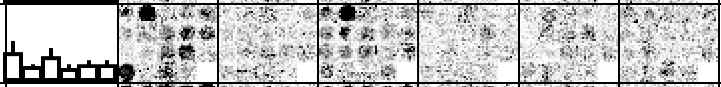							0.77
NM_013015	prostaglandin d synthase ptgds; synthetase; h2 d-isomerase	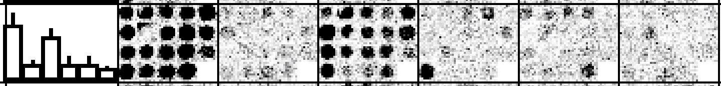							0.77
NM_031620	3-phosphoglycerate dehydrogenase phgdh; d-3-phosphoglycerate	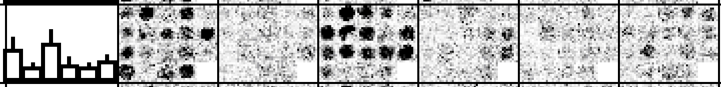							0.76
NM_031688	sensory neuron synuclein synuclein-like; synuclein, gamma	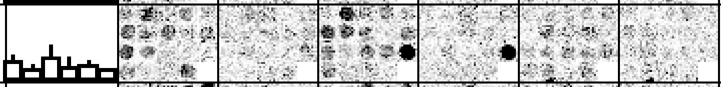							0.76
NM_022507	protein kinase c, zeta subspecies; c; pkcz	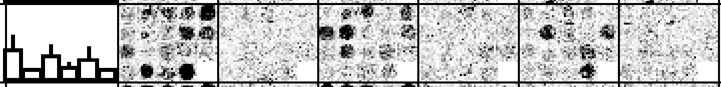							0.73
NM_017109	synapsin iiia synapsin iii; 3 syn3	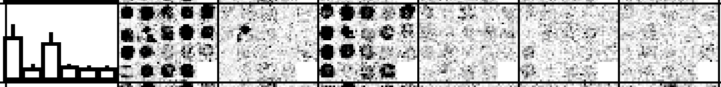							0.71
mwgrat10K#8905	expression: kidney; strains: wistar_kyoto; similar to pir	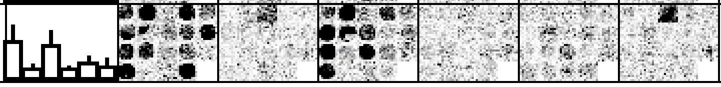							0.70

GeneID	GeneName	Signals	Brain	Heart	Kidney	Liver	Lung	Spleen	OSI
mwgrat10K#6161	expression: liver kidney heart brain; strains: shrsp sprague_dawley								1.00
NM_017111	solute carrier family organic anion transporter member 3 slc21a1; oatp								1.00
mwgrat10K#8349	expression: liver; strains: shrsp; similar to pir nf00847096								1.00
NM_031839	cytochrome p450 arachidonic acid epoxygenase cyp 2c23; cyp2c23;								1.00
U28504	na? cotransporter-1								1.00
mwgrat10K#6841	expression: liver kidney; strains: shrsp sprague_dawley wistar_kyoto;								1.00
NM_017013	glutathione-s-transferase, alpha type yc? gsta2; glutathione s-transferase								1.00
NM_012674	serine protease inhibitor, kanzal type 1/ trypsin inhibitor-like protein,								1.00
mwgrat10K#6834	expression: liver; strains: shrsp; similar to pir nf00160927 arylacetyl								1.00
mwgrat10K#7725	expression: liver kidney brain; strains: shrsp wistar_kyoto; similar								0.99
mwgrat10K#9161	expression: liver; strains: sprague_dawley; similar to								0.99
mwgrat10K#8727	expression: kidney; strains: wistar_kyoto; similar to								0.99
mwgrat10K#8969	expression: brain; strains: sprague_dawley; similar to pir								0.99
NM_017112	hepsin hpn								0.99
NM_020076_1	3-hydroxyanthranilate 3,4-dioxygenase haao								0.99
mwgrat10K#6833	expression: liver kidney; strains: shrsp wistar_kyoto; similar to								0.99
mwgrat10K#6485	expression: liver heart kidney; strains: shrsp sprague_dawley								0.99
mwgrat10K#7617	expression: liver kidney; strains: shrsp wistar_kyoto; similar to pir								0.99
mwgrat10K#7025	expression: liver heart brain kidney; strains: shrsp sprague_dawley								0.99
mwgrat10K#6436	expression: liver heart kidney; strains: shrsp sprague_dawley								0.99
NM_031985	s6 protein kinase; rps6kb1								0.99
mwgrat10K#7657	expression: liver kidney; strains: shrsp wistar_kyoto; similar to								0.99
X55995	dimethylglycine dehydrogenase								0.99

GeneID	GeneName	Signals	Brain	Heart	Kidney	Liver	Lung	Spleen	OSI
mwgrat10K#8694	expression: kidney; strains: wistar_kyoto; similar to								0.99
NM_012558	fructose-1,6-bisphosphatase fru-1,6-p2-ase;								0.99
AB037424	androgen-inducible aldehyde reductase aiar								0.99
mwgrat10K#9601	expression: kidney; strains: shrsp; similar to gbp|ak002457|ak002457_1								0.99
M92920	phosphorylase kinase beta-subunit phk-beta-subunit								0.99
D14046	'dna topoisomerase iib'								0.99
mwgrat10K#9170	expression: liver; strains: sprague_dawley; similar to								0.98
mwgrat10K#6773	expression: liver brain kidney; strains: shrsp sprague_dawley								0.98
NM_031057	methylmalonate semialdehyde dehydrogenase gene mmsdh								0.98
NM_031589	putative glycogen storage disease type 1b protein;								0.98
D11325	pancreatic secretory trypsin inhibitor type ii precursor								0.98
mwgrat10K#5595	expression: liver heart kidney; strains: shrsp sprague_dawley								0.98
M57719	cytochrome p-450 iva2 cyp4a2; p450 iva3								0.98
mwgrat10K#7335	expression: liver kidney brain; strains: shrsp wistar_kyoto; similar								0.98
NM_019373	apolipoprotein m apom								0.98
mwgrat10K#6789	expression: liver; strains: shrsp sprague_dawley wistar_kyoto;								0.97
mwgrat10K#6792	expression: heart; strains: wistar_kyoto; similar to pir								0.97
K03249	peroxisomal enoyl-coa: hydrotase-3-hydroxyacyl-coa								0.97
K03248	phosphoenolpyruvate carboxykinase								0.97
M22359	alpha-1-inhibitor iii								0.97
AF163318	putative n-acetyltransferase camello 1 cml1								0.97
mwgrat10K#7697	expression: liver kidney; strains: shrsp wistar_kyoto; similar to								0.97
AB019693	hp33								0.97

GeneID	GeneName	Signals	Brain	Heart	Kidney	Liver	Lung	Spleen	OSI
mwgrat10K#7513	expression: kidney; strains: shrsp; similar to gbp|u59185|u59185_1 mct	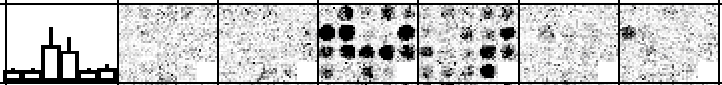							0.97
mwgrat10K#7361	expression: liver kidney; strains: shrsp wistar_kyoto; similar to	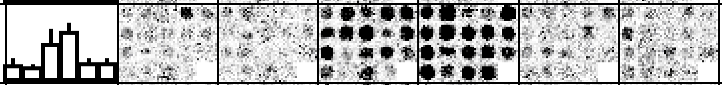							0.97
mwgrat10K#7081	expression: liver brain kidney; strains: shrsp wistar_kyoto; similar	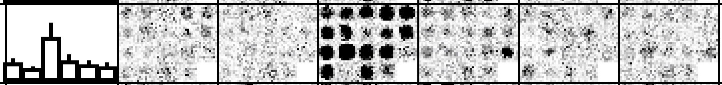							0.97
mwgrat10K#7530	expression: liver brain kidney; strains: shrsp wistar_kyoto; similar	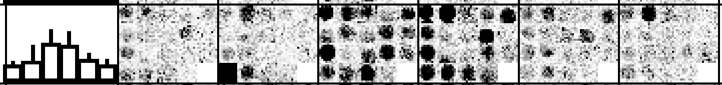							0.97
AB061719	diacetyl/l-xylulose reductase; glb	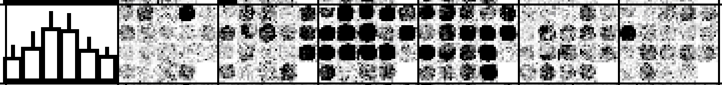							0.96
AJ005542	dimerization cofactor of hnf1 pterin-4a-carbinolamin dehydratase	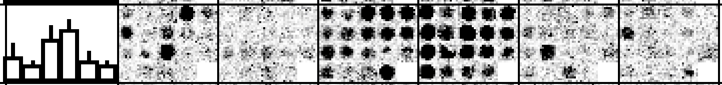							0.96
AB010632	carboxylesterase precursor	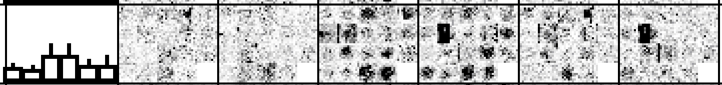							0.96
mwgrat10K#7401	expression: liver kidney; strains: shrsp wistar_kyoto; similar to	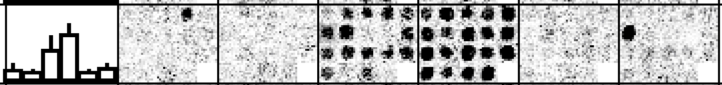							0.95
mwgrat10K#5599	expression: liver kidney; strains: shrsp wistar_kyoto; similar to pir	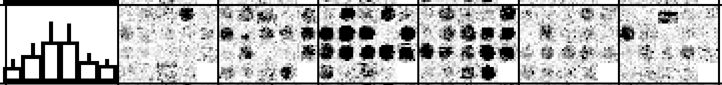							0.95
mwgrat10K#6525	expression: liver kidney; strains: shrsp wistar_kyoto; similar to	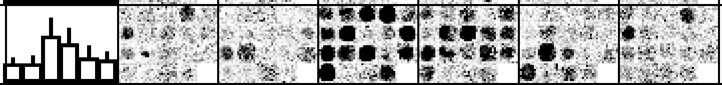							0.95
mwgrat10K#6651	expression: heart brain kidney; strains: shrsp wistar_kyoto; similar	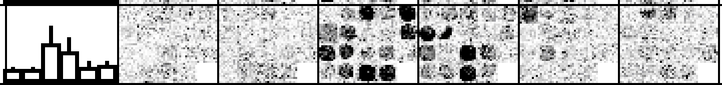							0.95
mwgrat10K#6820	expression: liver brain kidney; strains: shrsp sprague_dawley	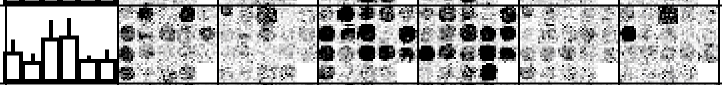							0.95
mwgrat10K#6637	expression: liver heart; strains: shrsp sprague_dawley wistar_kyoto;	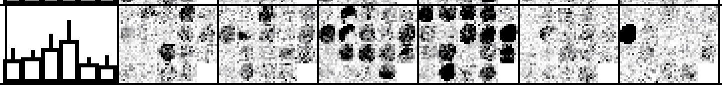							0.95
mwgrat10K#8333	expression: liver kidney; strains: shrsp; mwg own new gene sequence	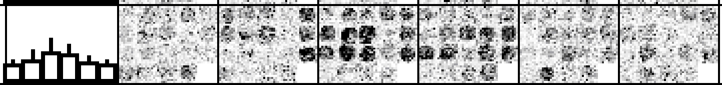							0.94
U20551	udp-glucuronosyltransferase precursor ugt1.1; udp	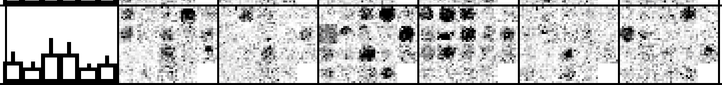							0.94
mwgrat10K#6856	expression: liver kidney; strains: shrsp sprague_dawley wistar_kyoto;	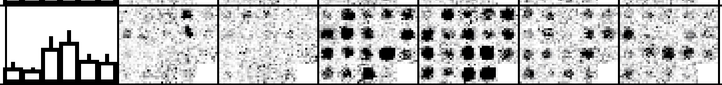							0.94
mwgrat10K#7456	expression: liver; strains: shrsp sprague_dawley wistar_kyoto;	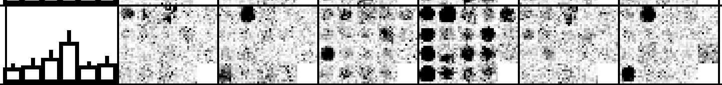							0.94
NM_017181	fumarylacetoacetate hydrolase fah	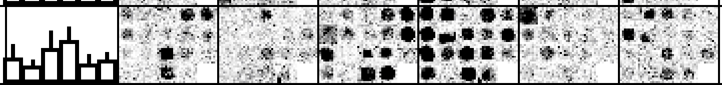							0.94
mwgrat10K#6304	expression: heart kidney; strains: shrsp sprague_dawley wistar_kyoto;	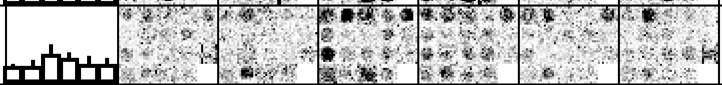							0.93
L04760	nucleotide binding protein	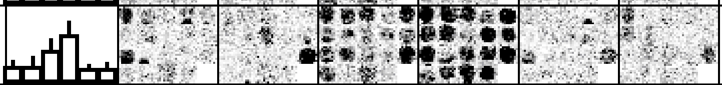							0.93
NM_053293	glutathione s-transferase 1 theta gstt1; transferase	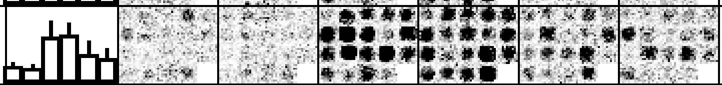							0.93
NM_017321	iron-responsive element-binding protein ratireb	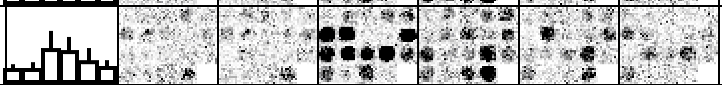							0.93
AF189709	collagen xviii	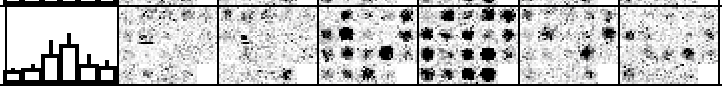							0.93

GeneID	GeneName	Signals	Brain	Heart	Kidney	Liver	Lung	Spleen	OSI
NM_031714	expression: liver kidney strains: shrsp sprague_dawley wistar_kyoto								0.93
NM_022853	solute carrier family 30 zinc transporter, member 1 slc30a1; znt-1								0.92
mwgrat10K#6799	expression: liver kidney; strains: shrsp sprague_dawley wistar_kyoto;								0.92
mwgrat10K#7645	expression: heart kidney; strains: shrsp wistar_kyoto; similar to								0.92
mwgrat10K#7336	expression: liver kidney; strains: shrsp sprague_dawley wistar_kyoto;								0.92
M77183	alpha-1-macroglobulin alpha-1-macrogloculin; alpha1 m								0.92
mwgrat10K#8878	expression: kidney heart brain; strains: shrsp sprague_dawley								0.91
NM_012588	insulin-like growth factor binding protein precursor igfbp-3;								0.91
mwgrat10K#6804	expression: liver kidney; strains: shrsp sprague_dawley wistar_kyoto;								0.91
AF120100	thiopurine s-methyltransferase tpmt								0.90
mwgrat10K#6572	expression: liver brain kidney; strains: shrsp sprague_dawley								0.90
mwgrat10K#6797	expression: liver kidney; strains: shrsp sprague_dawley wistar_kyoto;								0.90
mwgrat10K#6805	expression: liver; strains: shrsp sprague_dawley wistar_kyoto;								0.89
NM_031980_1	udp-glucuronosyltransferase; ugt2b12								0.89
NM_031749	glycoprotein processing glucosidase i; 1 gcs1-pending								0.89
NM_012879	solute carrier family 2 a2 gkucose transporter, type 2 slc2a2; glucose								0.89
NM_022599	outer membrane protein omp25; npw16								0.88
mwgrat10K#8721	expression: liver; strains: wistar_kyoto; similar to								0.88
mwgrat10K#8844	expression: kidney; strains: wistar_kyoto; similar to								0.88
NM_021653	thyroxine deiodinase, type i dio1; deiodinase								0.88
NM_021668	putative n-acetyltransferase camello 2 cml 2; cml5; cml2								0.88
mwgrat10K#7348	expression: liver kidney; strains: shrsp wistar_kyoto; similar to								0.87
NM_031325	udp-glucose dehydrogeanse ugdh								0.87

GeneID	GeneName	Signals	Brain	Heart	Kidney	Liver	Lung	Spleen	OSI
NM_017193	kynurenine/alpha-aminoadipate aminotransferase; kynurenine ii kat2								0.87
mwgrat10K# 7627	expression: kidney brain; strains: shrsp wistar_kyoto; similar to								0.87
mwgrat10K# 8230	expression: liver heart kidney; strains: shrsp sprague_dawley								0.87
mwgrat10K# 8248	expression: liver kidney heart brain; strains: shrsp sprague_dawley								0.86
NM_017170	serum amyloid p-component apcs; p component sap; seum								0.86
Y17321	h protein cdk102								0.86
mwgrat10K# 6810	expression: liver brain kidney; strains: shrsp sprague_dawley								0.85
mwgrat10K# 8402	expression: liver; strains: shrsp; similar to pir nf00512019								0.85
NM_013082	ryudocan/syndecan 2 sdc2; hspg core protein								0.85
mwgrat10K# 9571	expression: kidney; strains: shrsp; similar to gbp|bc013515|bc013515_1								0.85
NM_024381	atp-stimulated glucocorticoid-receptor translocaton								0.85
NM_012552	preproelastase i; elastase; 1 ela1								0.84
NM_017340	acyl-coa oxidase e.c 1.3.3.6; ratacoa1								0.84
NM_012844	epoxide hydrolase 1 microsomal xenobiotic hydrolase ephx1								0.84
NM_017201	s-adenosyl-l-homocysteine hydrolase ec 3.3.1.1;								0.83
mwgrat10K# 8297	expression: liver kidney; strains: shrsp sprague_dawley wistar_kyoto;								0.83
NM_022547	10-formyltetrahydrofolate dehydrogenase fthfd								0.82
NM_012730	cytochrome p450, subfamily iid2 cyp2d2; debrisoquine								0.82
NM_012816	alpha-methylacyl-coa racemase amacr; 2-arylpropionyl-coa								0.81
S82820	glutathione s-transferase yc2 subunit gsta5; transferase gst								0.81
mwgrat10K# 8714	expression: liver; strains: wistar_kyoto; similar to								0.81
NM_017084	glycine methyltransferase gnmt; aa 1-293								0.81
NM_021745	farnesoid x activated receptor loc60351								0.81

GeneID	GeneName	Signals	Brain	Heart	Kidney	Liver	Lung	Spleen	OSI
NM_031736	solute carrier family 27 fatty acid transporter, member 2 slc27a2;								0.80
NM_012697	organic cation transporter slc22a1; oct1a								0.80
NM_013098	glucose-6-phosphatase catalytic subunit g6pase; g6pc								0.80
NM_031543	cytochrome p450, subfamily 2e1 ethanol-inducible cyp2e1; p450;								0.80
NM_031835	beta-alanine-pyruvate aminotransferase beta-alaat ii; rat								0.79
NM_013144	insulin-like growth factor binding protein 1 igfbp1; igfbp-1; igf protein-1;								0.79
mwgrat10K#8814	expression: kidney; strains: wistar_kyoto; similar to								0.78
NM_012564	group-specific component vitamin d-binding protein gc; precursor; d								0.78
mwgrat10K#8674	expression: liver; strains: wistar_kyoto; similar to								0.78
NM_031546	senescence marker protein-30 smp30; regucalcin; rgn								0.77
NM_013048	tocopherol transfer protein alpha ttpa; alpha-tocopherol								0.77
U19485	spp-24 precursor								0.77
NM_031812	endolyn cd164								0.77
mwgrat10K#8729	expression: kidney; strains: wistar_kyoto; similar to pir								0.76
NM_032082	hydroxyacid oxidase 3 medium-chain hao3; s								0.76
U85512	gtp cyclohydrolase i feedback regulatory protein								0.76
NM_053845	ureidopropionase, beta upb1; beta-alanine synthase								0.76
NM_031510	cytosolic nadp-dependent isocitrate dehydrogenase; 1, soluble idh1								0.76
mwgrat10K#8681	expression: liver; strains: wistar_kyoto; similar to pir								0.75
NM_031332	organic anion transporter loc83500; 3 oat3								0.74
NM_031855	ketohexokinase khk								0.74
NM_013215	aflatoxin b1 aldehyde reductase afar								0.73
NM_031003	beta-alanine oxoglutarate aminotransferase; 4-aminobutyrate								0.73

GeneID	GeneName	Signals	Brain	Heart	Kidney	Liver	Lung	Spleen	OSI
NM_017307	solute carrier family 25 mitochondrial carrier citrate transporter precursor	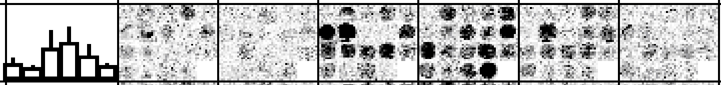							0.73
NM_012489	acetyl-coa acyltransferase, 3-oxo acyl-coa thiolase a, peroxisomal	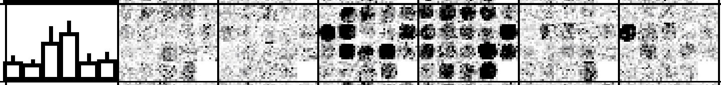							0.73
mwgrat10K# 8701	expression: liver; strains: shrsp; similar to gbp|u90535|u90535_1 fmo5	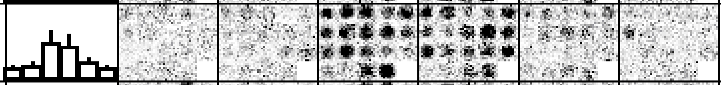							0.73
NM_017052	sorbitol dehydrogenase sord; l-iditol 2-dehydrogenase	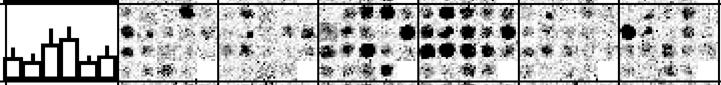							0.72
U04933	sodium/calcium exchanger 1 splice variant naca10 ncx1; na?exchanging	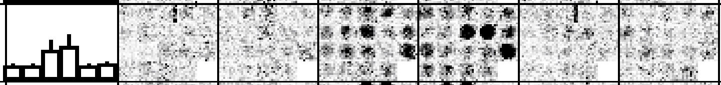							0.70
NM_012619	phenylalanine hydroxylase ec 1.14.16.1; pah	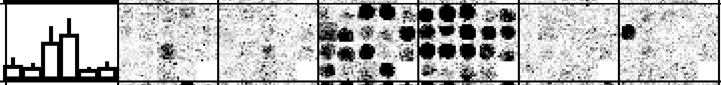							0.69
X81825	kidney microsomal carboxylesterase; rl1 cesrrl1; liver;	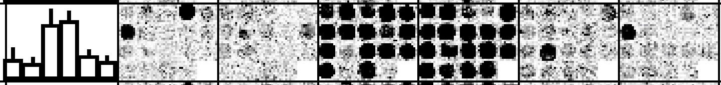							0.68
NM_017233	4-hydroxyphenylpyruvic acid dioxygenase hpd;	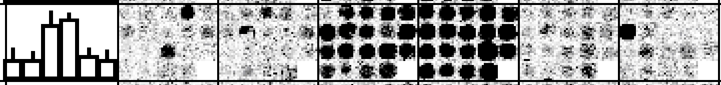							0.67
NM_021701 _1	human brain calcineurin beta subunit homolog calcium binding	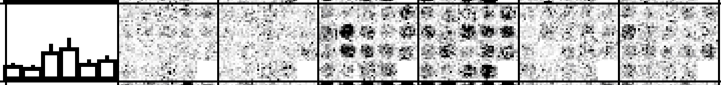							0.67
X02284	aldolase b	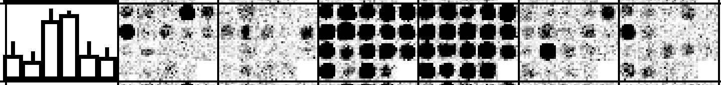							0.66
mwgrat10K# 8341	expression: liver; strains: shrsp; similar to pir nf00959315	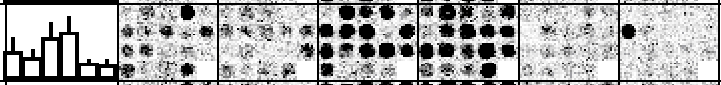							0.66
X12459	argininosuccinate synthetase aa 1-412	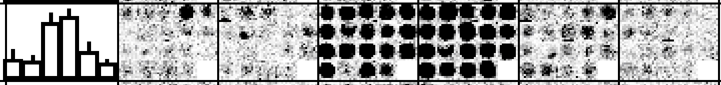							0.66
NM_019335	protein kinase, interferon-inducible double stranded rna dependent prkr;	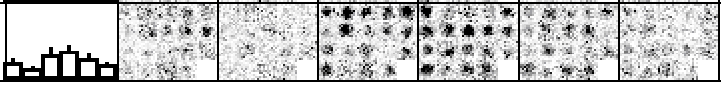							0.65

GeneID	GeneName	Signals	Brain	Heart	Kidney	Liver	Lung	Spleen	OSI
NM_019621	discs, large homolog 4 drosophila dlgh4; postsynaptic density protein	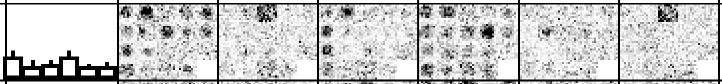							0.99
mwgrat10K#6928	expression: brain; strains: shrsp wistar_kyoto; similar to	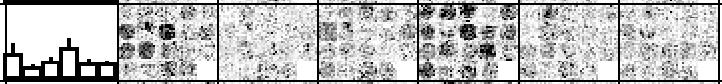							0.92
D38035_1	thyroid transcription factor-1 ttf-1; nuclear factor 1 tff-1	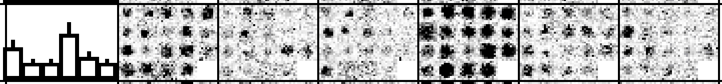							0.90
NM_031732	expression: liver strains: shrsp gbp|l22339|l22339_1 st1c1	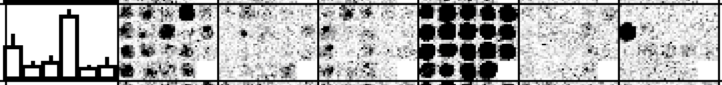							0.89
NM_053746	contactin 5 cntn5; nb-2	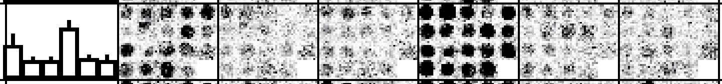							0.88
mwgrat10K#8690	expression: liver; strains: wistar_kyoto; similar to	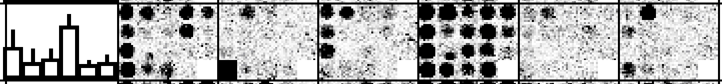							0.88
NM_017055	transferrin tf	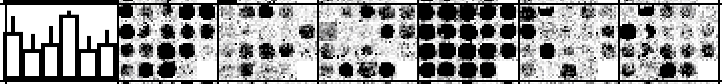							0.86
NM_017351	pre-alpha-inhibitor, heavy chain 3 paihc3	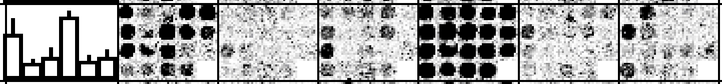							0.84
NM_030832	brain lipid binding protein fabp7; fatty acid	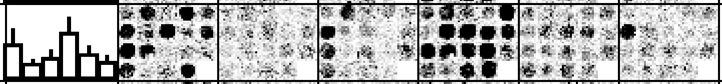							0.82
NM_053988	calbindin 2 calb2; calretinin	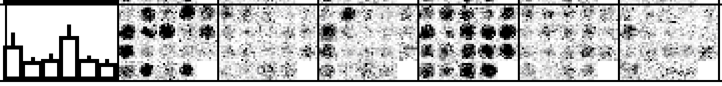							0.80

GeneID	GeneName	Signals	Brain	Heart	Kidney	Liver	Lung	Spleen	OSI
mwgrat10K#8459	expression: brain; strains: wistar_kyoto; similar to	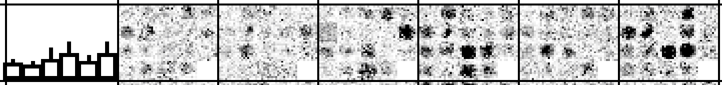							0.92
AF198533	lymphoid enhancer factor-1 lef-1	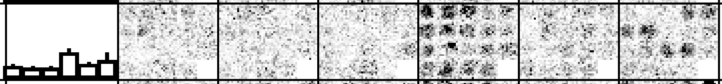							0.92
mwgrat10K#7184	expression: liver kidney brain; strains: shrsp sprague_dawley	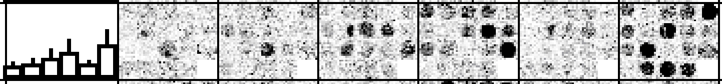							0.90
NM_012938	cathepsin e, two slightly different products a and b may be due to	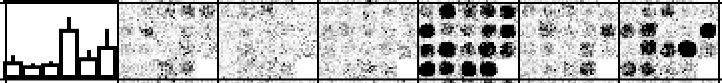							0.88
NM_024356	gtp cyclohydrolase 1 gch; i	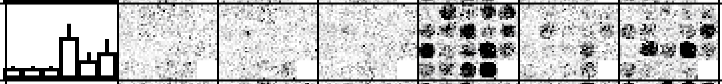							0.86
NM_053907	deoxyribonuclease i-like 3 dnase1l3; dnasey; dnase gamma	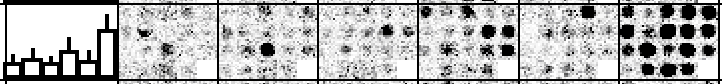							0.84
NM_017336_1	receptor-type protein tyrosine phosphatase d30 ptpro	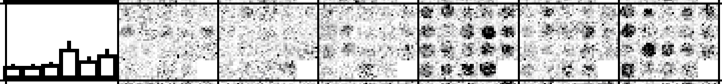							0.80
X73371	fc gamma receptor type 2	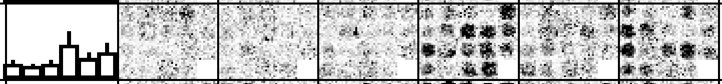							0.78
NM_024157	factor i protein factor i; complement cfi	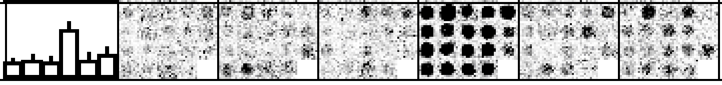							0.77

GeneID	GeneName	Signals	Brain	Heart	Kidney	Liver	Lung	Spleen	OSI
mwgrat10K#8109	expression: kidney brain; strains: shrsp sprague_dawley; similar to pir								0.98
AF060173	sv2 related protein svop								0.98
NM_012794	glycosylation dependent-cell adhesion molecule 1 glycam1;								0.96
X62839	voltage-gated potassium channel								0.96
mwgrat10K#7095	expression: brain; strains: shrsp wistar_kyoto; similar to								0.94
AF062038	glycoprotein-39 precursor								0.89
mwgrat10K#6267	expression: heart brain; strains: shrsp sprague_dawley wistar_kyoto;								0.87
NM_053601_1	neuronatin alpha; nnat								0.86
NM_031786	ring finger protein 22 rnf22								0.85
NM_019250	ral guanine nucleotide dissociation stimulator ralgds								0.79

GeneID	GeneName	Signals	Brain	Heart	Kidney	Liver	Lung	Spleen	OSI
NM_012612	natriuretic peptide precursor a, pronatriodilatin, also anf, pnd nppa;	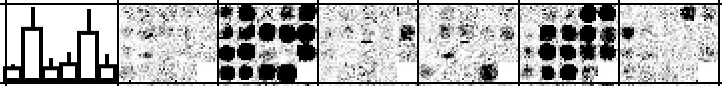							0.99
NM_031050	lumican, secretory intersticial proteoglycan; lumican lum	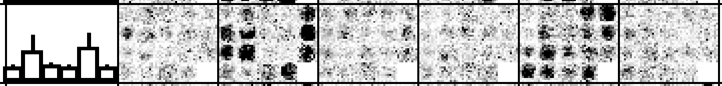							0.99
NM_013062 _1	flk1 kinase insert domain receptor; a type iii receptor tyrosine kinase; vegf	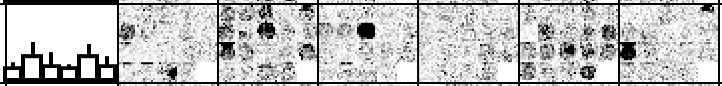							0.98
mwgrat10K# 6137	expression: heart brain kidney; strains: sprague_dawley	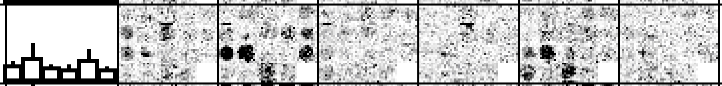							0.97
M92074	troponin i	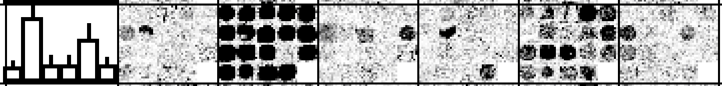							0.94
NM_013101	cyclic amp phosphodiesterase pde4a10; 3',5'-cyclic; camp; 4a	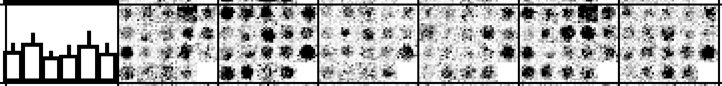							0.93
NM_023090	hypoxia inducible factor 2 alpha hif-2a; endothelial pas domain	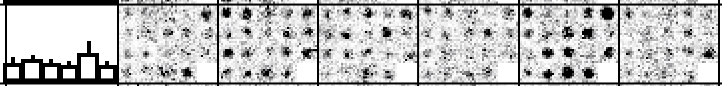							0.88
NM_019185	dna binding protein gata-gt1; gata-binding 6 gata6	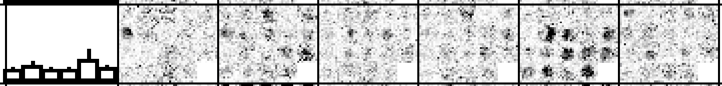							0.88
mwgrat10K# 8201	expression: kidney brain heart; strains: shrsp sprague_dawley	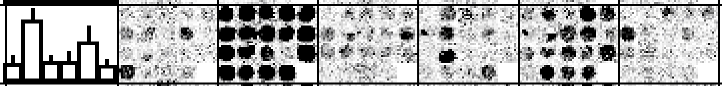							0.87
NM_017328	phosphoglycerate mutase ec 5.4.2.1; 2 pgam2;	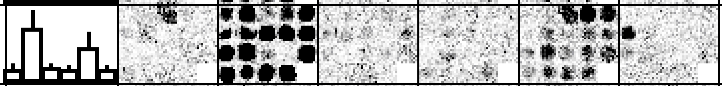							0.86
mwgrat10K# 8606	expression: heart; strains: wistar_kyoto; similar to pir	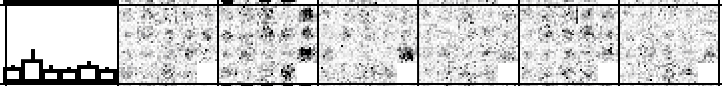							0.86
mwgrat10K# 8197	expression: brain heart; strains: shrsp sprague_dawley wistar_kyoto;	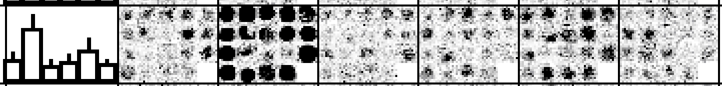							0.85
NM_021858	g protein beta-subunit gene loc60449; g-protein	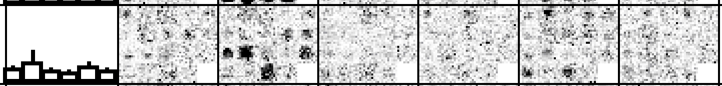							0.85
NM_019229	furosemide-sensitive k-cl cotransporter kcc1; solute carrier	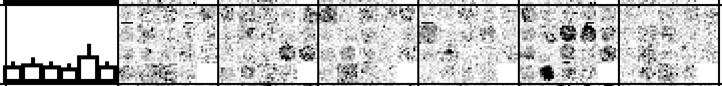							0.84
mwgrat10K# 9493	expression: heart; strains: shrsp; similar to gbp|l76227|l76227_1 von	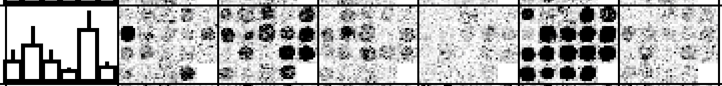							0.84
mwgrat10K# 7550	expression: liver heart kidney; strains: shrsp wistar_kyoto; similar	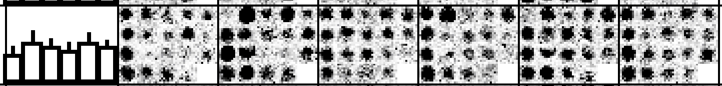							0.82
U46149	coq7	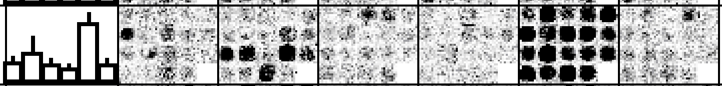							0.81
NM_057208 _1	striated muscle alpha tropomyosin aa 81-284; nonmuscle 5 tpm5;	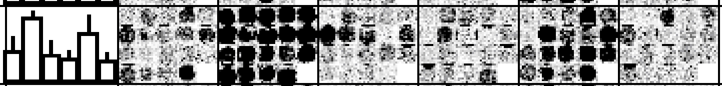							0.81
NM_012812	cytochrome c oxidase subunit via polypeptide 2 heart cox6a2; aa 1 -	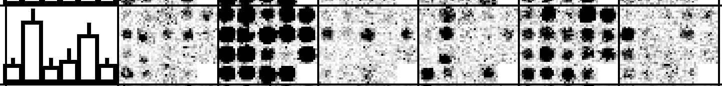							0.80
NM_022501	crp2 cysteine-rich protein 2 crp2; csrp2	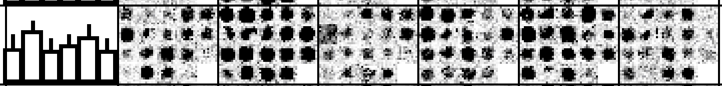							0.80
X70369	pro1 collagen type iii; alpha 1	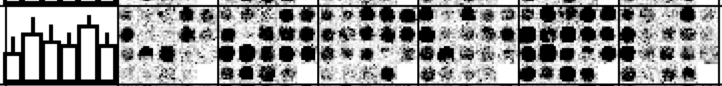							0.76
X00306	alpha-actin cardiac; actin	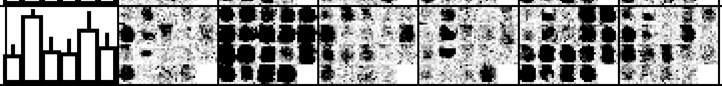							0.76
NM_019212	actin, alpha 1, skeletal muscle acta1; actin	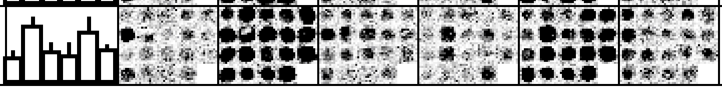							0.72

GeneID	GeneName	Signals	Brain	Heart	Kidney	Liver	Lung	Spleen	OSI
NM_012608	membrane metallo-endopeptidase neutral endopeptidase/enkephalinase	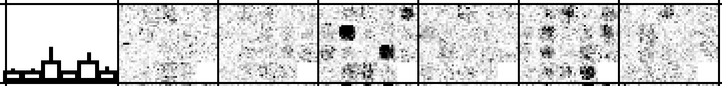							1.00
mwgrat10K# 7678	expression: kidney; strains: shrsp wistar_kyoto; similar to	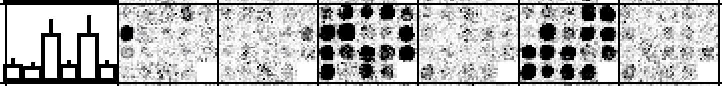							1.00
AJ249229	t-cell receptor delta chain tcrd	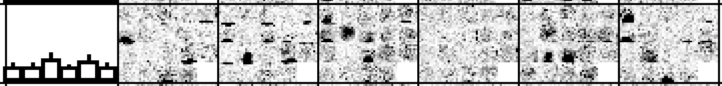							0.99
NM_021751	fudenine loc60357; prominin	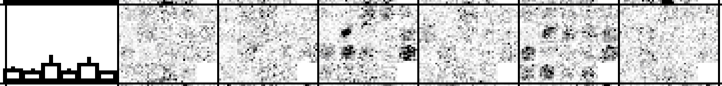							0.99
NM_012925	cd59 protein precursor; antigen	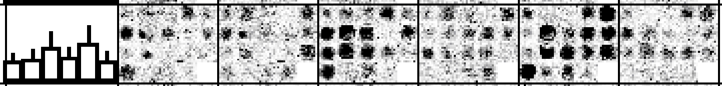							0.98
NM_019232	serum/glucocorticoid regulated kinase sgk; serine/threonine protein	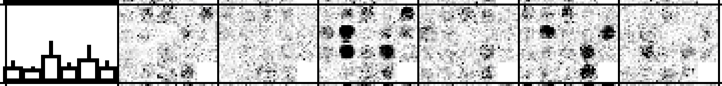							0.97
mwgrat10K# 6376	expression: liver brain kidney; strains: shrsp sprague_dawley	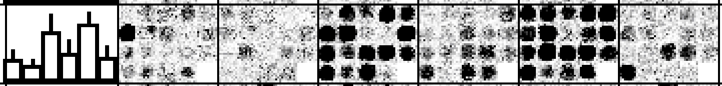							0.97
mwgrat10K# 7269	expression: brain kidney heart; strains: sprague_dawley	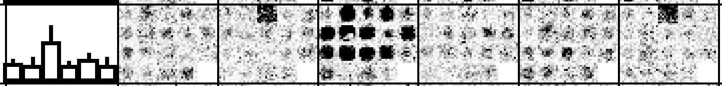							0.97
mwgrat10K# 6933	expression: brain kidney; strains: shrsp wistar_kyoto; similar to	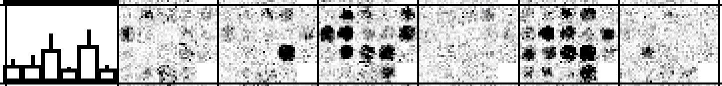							0.97
Y07832	protein tyrosine phosphatase brl-1	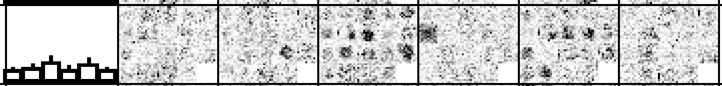							0.96
NM_024155	zap 36/annexin iv anxa4	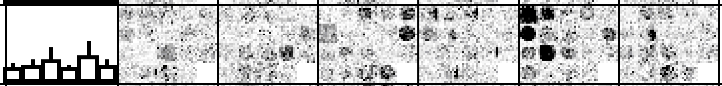							0.96
NM_024369	follistatin-related protein precursor fstl	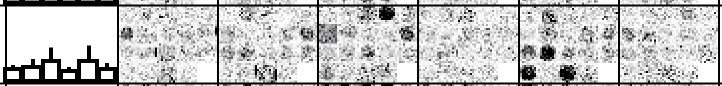							0.96
mwgrat10K# 7450	expression: liver brain kidney; strains: shrsp wistar_kyoto; similar	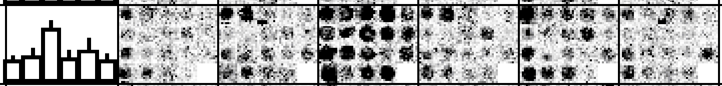							0.96
M30689_1	ly6-b antigen put. putative; ly6-a	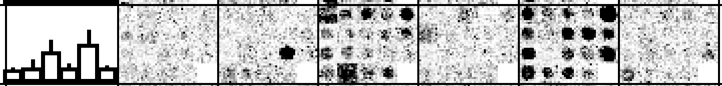							0.96
AF380194	trace amine receptor 6; ta6	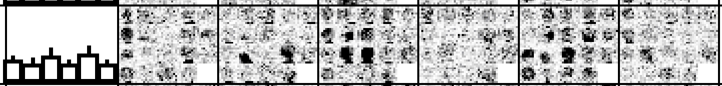							0.96
mwgrat10K# 7961	expression: kidney brain heart; strains: shrsp sprague_dawley	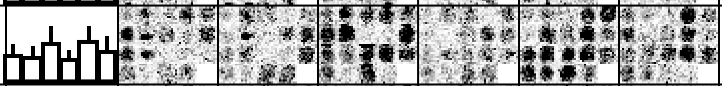							0.95
NM_013151	tissue-type plasminogen activator protein; t-pa; activator, tissue plat	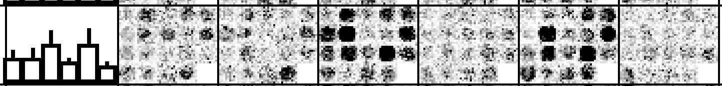							0.95
D87247	fructose-6-phosphate 2-kinase/fructose-2,6-bisphosphatas	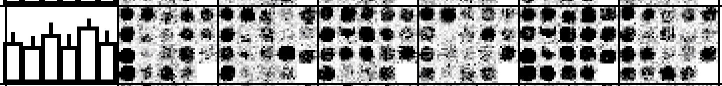							0.95
mwgrat10K# 9297	expression: brain; strains: shrsp; similar to gbp|ak020980|ak020980_1	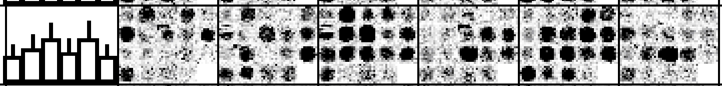							0.95
mwgrat10K# 9325	expression: brain; strains: shrsp; similar to pir nf00526980	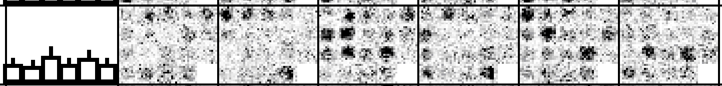							0.95
NM_012886	tissue inhibitor of metalloproteinase 3 timp-3; timp3	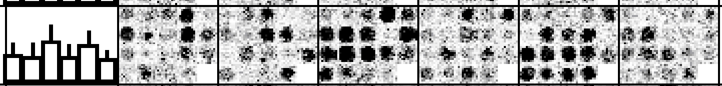							0.95
mwgrat10K# 7960	expression: brain kidney; strains: shrsp; similar to	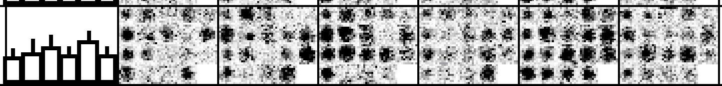							0.95
mwgrat10K# 7410	expression: liver brain kidney; strains: shrsp sprague_dawley	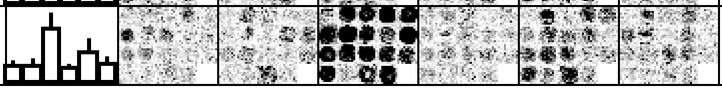							0.94

GeneID	GeneName	Signals	Brain	Heart	Kidney	Liver	Lung	Spleen	OSI
AY028455	inwardly rectifying potassium channel kir4.2a; kcnj15	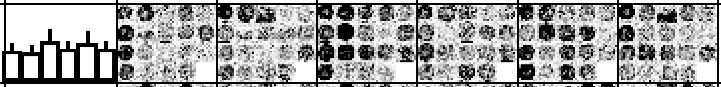							0.94
mwgrat10K#7400	expression: liver brain; strains: shrsp wistar_kyoto; similar to	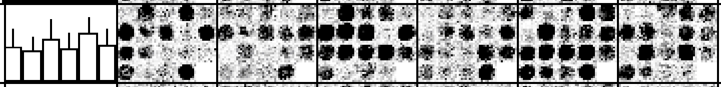							0.94
U26310	tensin tns	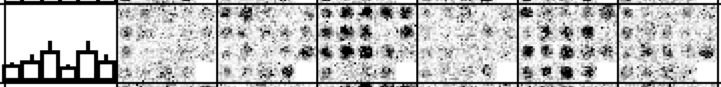							0.94
AJ001044	egp-314 protein homologue	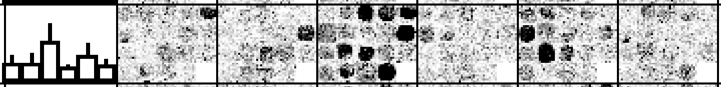							0.93
AF387513	kinase-deficient tgfbeta superfamily receptor subunit	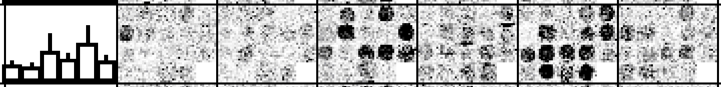							0.93
NM_022407	aldehyde dehydrogenase 1, subfamily a1 aldh1a1; aldh	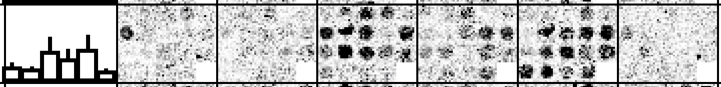							0.93
NM_022534	transcobalamin ii precursor tcn2p; tcii	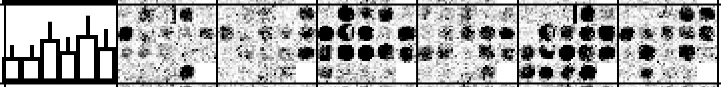							0.93
mwgrat10K#9127	expression: brain; strains: shrsp sprague_dawley; similar to	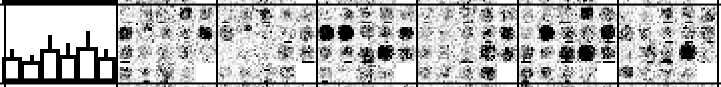							0.93
mwgrat10K#7333	expression: liver brain heart; strains: shrsp sprague_dawley wistar_kyoto;	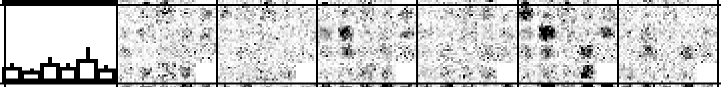							0.93
mwgrat10K#7871	expression: heart; strains: sprague_dawley; similar to	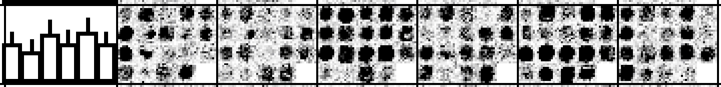							0.92
AJ011811	claudin-7 cldn7	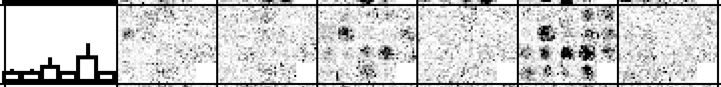							0.92
mwgrat10K#8062	expression: kidney; strains: shrsp wistar_kyoto; similar to	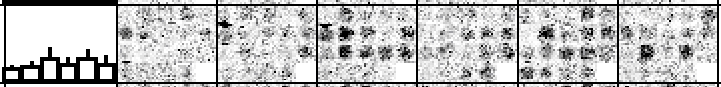							0.92
mwgrat10K#7501	expression: brain kidney; strains: shrsp wistar_kyoto; similar to	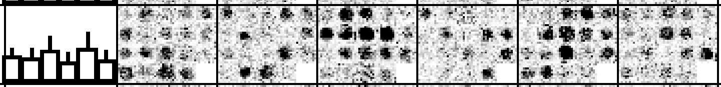							0.92
NM_023977	trans-golgi protein gmx33 gmx33	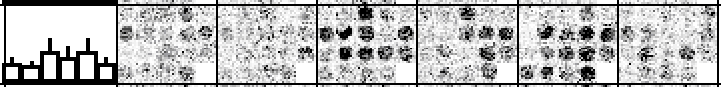							0.92
mwgrat10K#7689	expression: liver kidney brain; strains: sprague_dawley	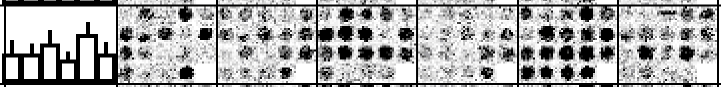							0.91
NM_017125	cd63 antigen cd63; ad1-antigen	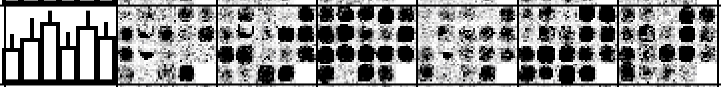							0.91
NM_013160	max interacting protein 1 mxi1; rmxi1	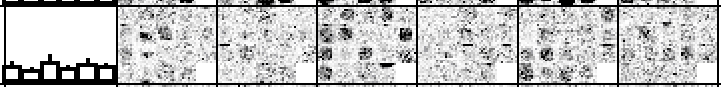							0.91
mwgrat10K#8017	expression: brain kidney; strains: shrsp; similar to	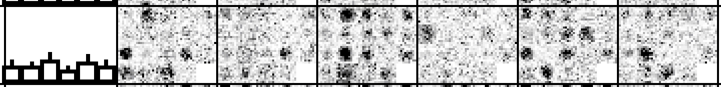							0.90
mwgrat10K#6196	expression: liver brain heart kidney; strains: shrsp sprague_dawley	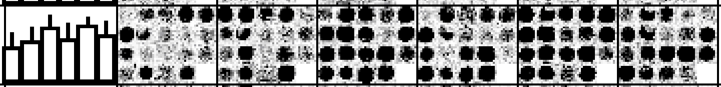							0.90
L20319	developmentally regulated protein tpo1	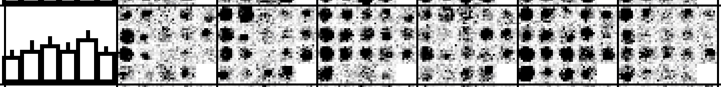							0.90
NM_019246	proprotein convertase subtilisin/kexin type 7 pcsk7; serine proteinase rpc7	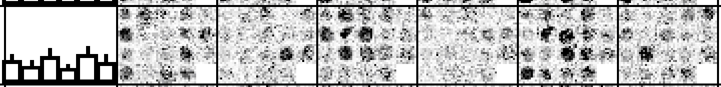							0.90
AJ002940	retinoic acid receptor alpha1	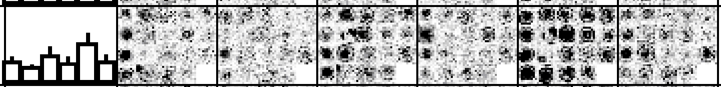							0.90
mwgrat10K#6383	expression: liver heart brain kidney; strains: shrsp wistar_kyoto; similar	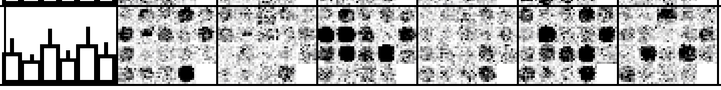							0.90

GeneID	GeneName	Signals	Brain	Heart	Kidney	Liver	Lung	Spleen	OSI
mwgrat10K#7577	expression: brain kidney; strains: shrsp wistar_kyoto; similar to pir	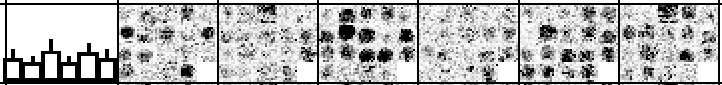							0.89
mwgrat10K#7297	expression: liver brain heart; strains: shrsp sprague_dawley wistar_kyoto;	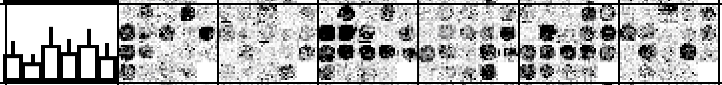							0.89
mwgrat10K#7134	expression: brain; strains: sprague_dawley wistar_kyoto;	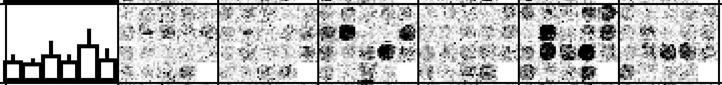							0.89
NM_022951	putative protein phosphatase 1 nuclear targeting subunit ppp1r10;	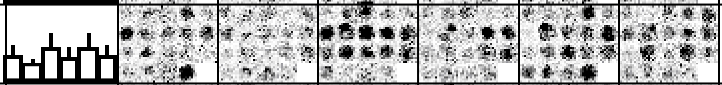							0.89
NM_020087	notch 3 protein; notch3	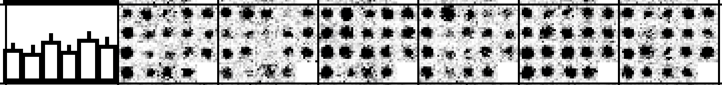							0.89
NM_031560	cathepsin k ctsk	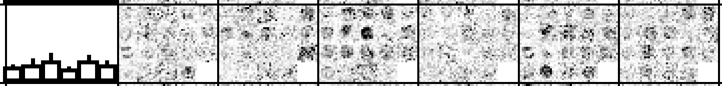							0.88
mwgrat10K#6779	expression: liver heart kidney; strains: shrsp sprague_dawley	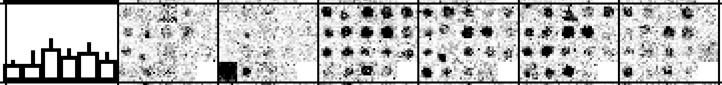							0.88
mwgrat10K#8503	expression: brain; strains: wistar_kyoto; similar to	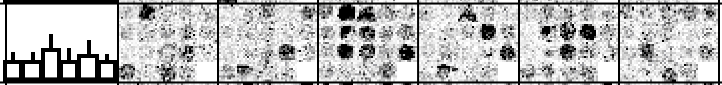							0.88
NM_022525	plasma glutathione peroxidase precursor gpxp	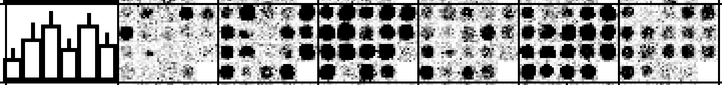							0.88
X78949	prolyl 4-hydroxylase, alpha subunit p4halpha	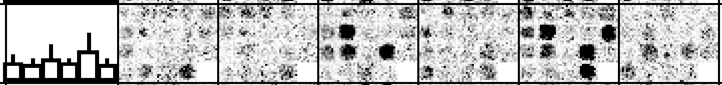							0.88
NM_057194	phospholipid scramblase 1; plscr1	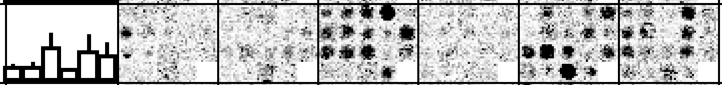							0.88
mwgrat10K#8078	expression: heart kidney; strains: shrsp; similar to	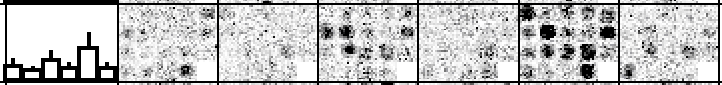							0.88
mwgrat10K#8337	expression: liver brain kidney heart; strains: shrsp wistar_kyoto; mwg	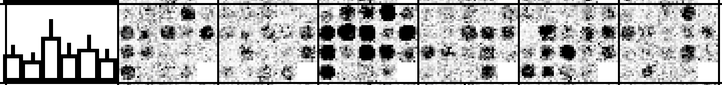							0.87
NM_053996	high affinity l-proline transporter; proline prot	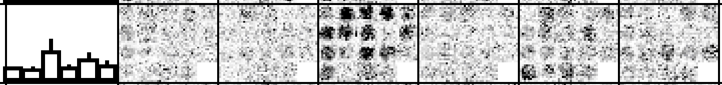							0.87
mwgrat10K#9233	expression: brain; strains: sprague_dawley; similar to pir	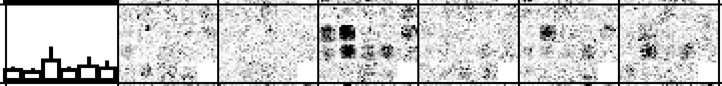							0.87
mwgrat10K#9580	expression: liver; strains: shrsp; similar to pir nf00517475 zyxin	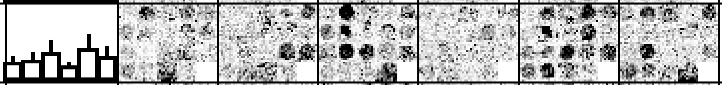							0.87
mwgrat10K#6378	expression: heart; strains: wistar_kyoto; similar to	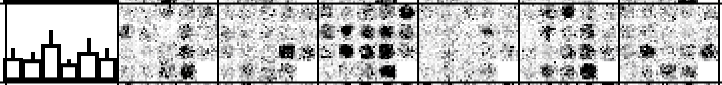							0.87
mwgrat10K#9630	expression: kidney; strains: shrsp; similar to pir nf00523520 mac25 -	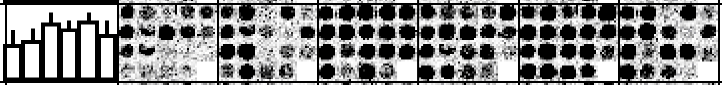							0.86
X67788	ezrin, p81	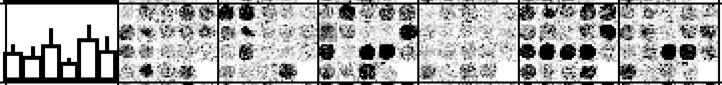							0.86
NM_031670	kidney-derived aspartic protease-like protein kdap; napsin nap	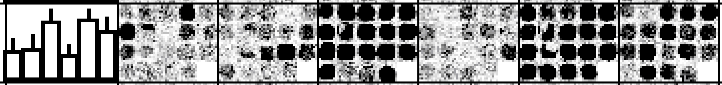							0.86
NM_021587	transforming growth factor-beta tgf-beta masking protein large	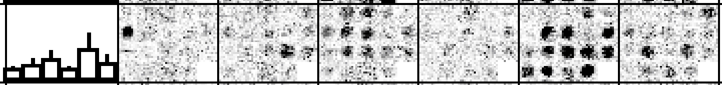							0.86
NM_019202	phospholipase a2, group iic pla2g2c; 14 kda a2	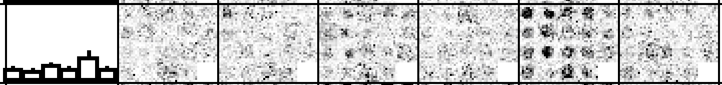							0.85
NM_020073	parathyroid hormone/parathyroid hormone related protein receptor;	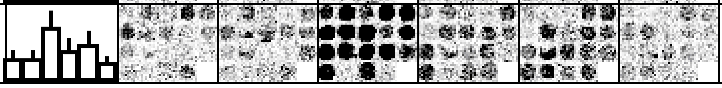							0.85

GeneID	GeneName	Signals	Brain	Heart	Kidney	Liver	Lung	Spleen	OSI
mwgrat10K#8106	expression: brain kidney; strains: shrsp sprague_dawley; similar to								0.84
NM_012792	flavin-containing monooxygenase 1 fmo-1; fmo1								0.84
NM_012908	apoptosis apo-1 antigen ligand 1 fas antigen ligand tnfsf6; for								0.83
NM_031605	cytochrome p450, 4a10 cyp4a10; p450								0.83
mwgrat10K#7979	expression: kidney heart brain; strains: shrsp sprague_dawley;								0.83
NM_019283	antigen identified by monoclonal antibodies 4f2 mdu1; heavy chain								0.83
NM_057193	interleukin-10 receptor, alpha-chain il-10ra; interleukin 10 alpha il10ra								0.82
NM_012615_4	ornithine decarboxylase ec 4.1.1.17; odc; ornitine odc1								0.82
U61772	merlin nf2								0.82
NM_019159	synapsin ii syn2; 2a; 2b								0.81
NM_012858	luteinizing hormone; lutropin subunit beta; lhb								0.80
mwgrat10K#8045	expression: brain kidney; strains: shrsp; similar to								0.80
mwgrat10K#8829	expression: kidney; strains: wistar_kyoto; similar to								0.79
mwgrat10K#8800	expression: kidney; strains: wistar_kyoto; similar to								0.79
mwgrat10K#8997	expression: brain; strains: sprague_dawley; similar to								0.79
S68809	s100a1 protein s100a1; s100 alpha								0.79
NM_013059	tissue-nonspecific alp alkaline phosphatase alpl; precursor								0.79
NM_053819_1	tissue inhibitor of metalloproteinase 1 timp1; timp-1; matrix;								0.78
mwgrat10K#8070	expression: heart brain; strains: shrsp sprague_dawley; similar to pir								0.78
NM_031819	fat tumor suppressor drosophila homolog fat; protocadherin								0.78
mwgrat10K#8750	expression: kidney; strains: wistar_kyoto; similar to								0.78
NM_022585	ornithine decarboxylase antizyme inhibitor oazi								0.78
NM_013143	endopeptidase-24.18 alpha subunit endopeptidase-24.18 alpha subunit,								0.76

GeneID	GeneName	Signals	Brain	Heart	Kidney	Liver	Lung	Spleen	OSI
mwgrat10K#8176	expression: liver heart; strains: wistar_kyoto; similar to pir	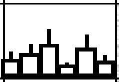	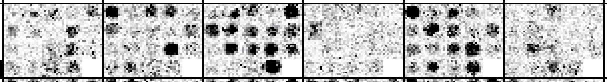						0.75
NM_012578	histone h10 h1 subtype; h1-0 h1f0; 0	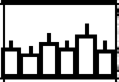	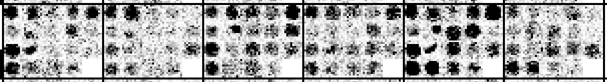						0.74
NM_031329	occludin ocln	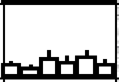	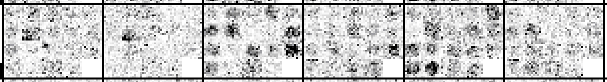						0.71
NM_058213_1	atpase, ca transporting, cardiac muscle, fast twitch 1 atp2a1;	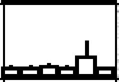	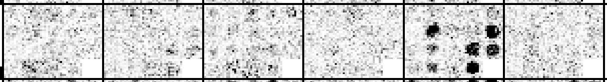						0.69
NM_012904	annexin 1 p35 lipocortin 1 anx1; calpactin ii; i; aa - 346	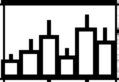	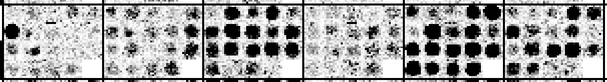						0.64
mwgrat10K#8807	expression: kidney; strains: wistar_kyoto; similar to	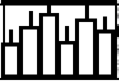	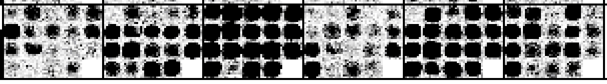						0.62

GeneID	GeneName	Signals	Brain	Heart	Kidney	Liver	Lung	Spleen	OSI
NM_019303	cytochrome p450, subfamily iif, polypeptide 1 cyp2f1; p4502f4								1.00
mwgrat10K# 8388	expression: liver; strains: shrsp; similar to gbp|af220944|af220944_1								0.99
NM_012779	aquaporin 5 aqp5; aquaporin-5								0.96
NM_031237	ubiquitin-conjugating enzyme e2d 3; homologous to yeast ubc4/5; ube2d3								0.94
NM_053611	nuclear proten 1 nupr1; p8								0.94
mwgrat10K# 6774	expression: liver kidney; strains: shrsp sprague_dawley wistar_kyoto;								0.93
mwgrat10K# 8266	expression: liver kidney brain heart; strains: shrsp sprague_dawley								0.92
mwgrat10K# 6670	expression: liver kidney heart brain; strains: shrsp sprague_dawley								0.92
mwgrat10K# 6312	expression: liver heart brain; strains: sprague_dawley wistar_kyoto;								0.90
mwgrat10K# 7479	expression: liver; strains: shrsp sprague_dawley wistar_kyoto;								0.90
L46791	precursor polypeptide aa -18 to 547; carboxylesterase; cholesterol								0.90
mwgrat10K# 7085	expression: liver heart brain; strains: sprague_dawley wistar_kyoto;								0.88
AF022090	guanine nucleotide binding protein gamma 10 subunit								0.88
mwgrat10K# 7326	expression: liver; strains: sprague_dawley wistar_kyoto;								0.86
NM_022298 _12	alpha-tubulin tuba1								0.86
mwgrat10K# 6957	expression: liver brain; strains: shrsp sprague_dawley wistar_kyoto;								0.85
mwgrat10K# 7415	expression: liver; strains: shrsp wistar_kyoto; similar to								0.85
NM_053528	dna polymerase gamma mip1; polg								0.85
mwgrat10K# 6803	expression: liver kidney heart; strains: shrsp sprague_dawley								0.84
mwgrat10K# 8331	expression: heart brain; strains: shrsp sprague_dawley; mwg own								0.84
NM_031698	ribophorin rpn2; ii								0.84
U75305	perlecan								0.83
mwgrat10K# 6580	expression: heart brain; strains: shrsp sprague_dawley wistar_kyoto;								0.82

GeneID	GeneName	Signals	Brain	Heart	Kidney	Liver	Lung	Spleen	OSI
NM_053599	ephrin a1 efna1; b61	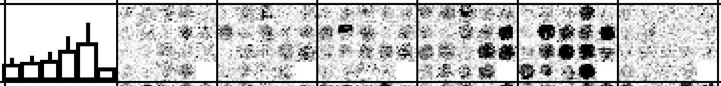							0.80
AF110267	golgi stacking protein homolog grasp55	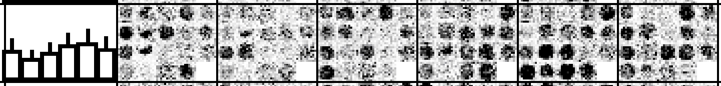							0.80
M63482	cytokeratin 8 polypeptide	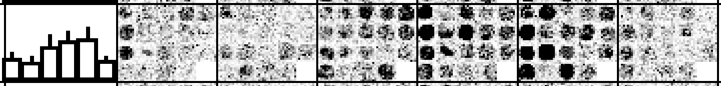							0.80
NM_012688	cholecystokinin type-a receptor cckar; a	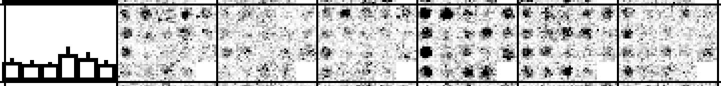							0.80
mwgrat10K#9109	expression: heart; strains: sprague_dawley; similar to	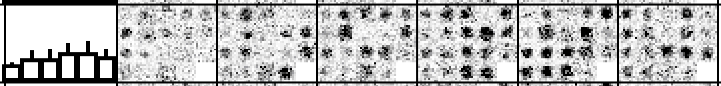							0.79
mwgrat10K#8702	expression: liver; strains: wistar_kyoto; similar to pir	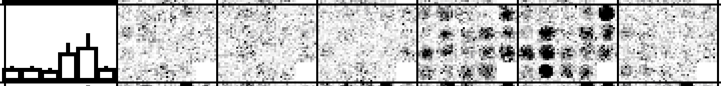							0.78
mwgrat10K#8442	expression: brain; strains: wistar_kyoto; similar to	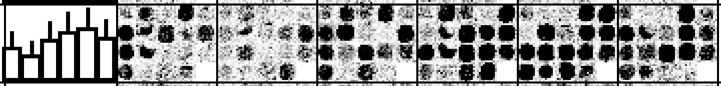							0.78
mwgrat10K#9495	expression: heart; strains: shrsp; similar to gbp|bc003947|bc003947_1	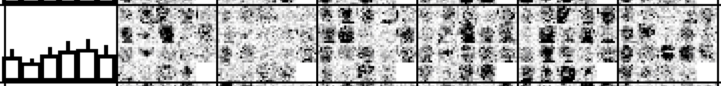							0.77
X62888	fatty-acid synthase; fas	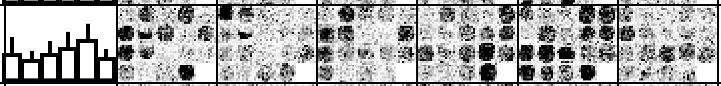							0.77
NM_053526	carboxypeptidase n cpn	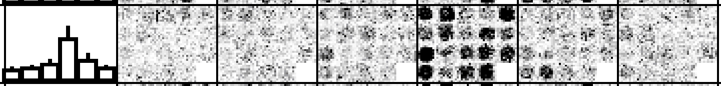							0.77
NM_017156	cytochrome p450, 2b19 cyp2b15; p450 2b15; p450iib12	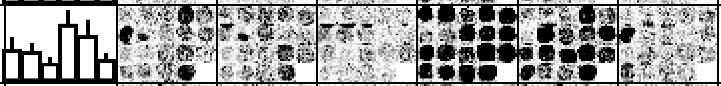							0.74
mwgrat10K#8706	expression: liver; strains: wistar_kyoto; similar to	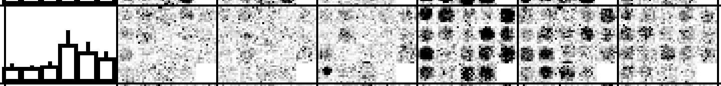							0.74
NM_019377	14-3-3 protein beta subtype 14-3-3 protein beta subtype, 14-3-3 beta;	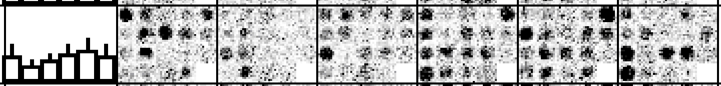							0.69
mwgrat10K#8670	expression: liver; strains: wistar_kyoto; similar to	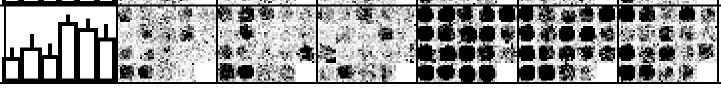							0.68

GeneID	GeneName	Signals	Brain	Heart	Kidney	Liver	Lung	Spleen	OSI
X83094	heat shock transcription factor 1 hsf1								1.00
NM_021694	lsc protein lsc								0.99
NM_019222	coronin, actin binding protein 1b coro1b; coronin-like								0.99
AF136231	interleukin-1-beta-converting enzyme and ced-3 homolog-1, long isoform;								0.99
D87926	cinc-2 beta cytokine-induced neutrophil chemoattractant-2 beta;								0.99
NM_012762	interleukin-1 beta converting enzyme il1bce; caspase-1 ice; interleukin								0.99
NM_053822	s100 calcium-binding protein a8 calgranulin a s100a8; intracellular								0.99
L22654_1	immunoglobulin gamma-2a chain immunoglobulin gamma-2a; heavy;								0.99
NM_012523	leukocyte antigen mrc-ox44 ox44; ox-44 cd53								0.98
AB028626	r-ras gtpase activating protein r-ras gap								0.98
NM_031772	rna polymerase i 194 kda subunit rpo1-4; rpa1								0.98
mwgrat10K#6894	expression: liver brain; strains: sprague_dawley wistar_kyoto;								0.98
NM_031512	interleukin 1 beta il1b; 1-beta								0.98
mwgrat10K#9483	expression: heart; strains: shrsp; similar to pir nf00528641 protein								0.98
AF452647	nesprin-1								0.98
NM_022194	interleukin 1 receptor antagonist gene il1rn; il1ra								0.98
mwgrat10K#9582	expression: kidney; strains: shrsp; similar to gbp|af002715|af002715_1								0.98
AB002466	rat nbp60								0.98
NM_013221	hmg-box containing protein 1 hbp1								0.98
AB023781	cathepsin y								0.98
mwgrat10K#8785	expression: kidney; strains: wistar_kyoto; similar to pir								0.97
AB015308	gtp binding protein alpha 15								0.97
mwgrat10K#7844	expression: brain heart; strains: shrsp sprague_dawley; similar to								0.97

GeneID	GeneName	Signals	Brain	Heart	Kidney	Liver	Lung	Spleen	OSI
AB026665	peptide histidine transporter 1 homolog rpht2; rpht2								0.97
NM_012924_1	cell surface glycoprotein cd44; hyaluronate binding protein; cd44								0.97
AF169637	paired ig-like receptor-a1 pir-a1; receptor-a2 pir-a2								0.97
M80367	orf								0.97
AF181251	lung kruppel-like factor lklf								0.97
NM_012736	glycerol-3-phosophate dehydrogenase 2 mitochondrial								0.96
NM_031695	gal beta 1,3-galnac alpha-2,3-sialyltransferase;								0.96
mwgrat10K#6718	expression: heart brain kidney; strains: shrsp wistar_kyoto; similar								0.96
AB023065	o-sialoglycoprotease prsmg1/gcpl1								0.96
NM_031777	upstream transcription factor 1 usf1; usf-1								0.96
L08446	fc gamma receptor								0.96
NM_012892	amiloride-sensitive cation channel 1, neuronal degenerin, two different								0.96
AF307852	actin-related protein 3 arp3								0.96
NM_012555	ets avian erythroblastosis virus e2 oncogene homolog 1 tumor								0.96
AF277899	adapter protein ath-55								0.96
NM_021866	chemokine receptor ccr2 gene ccr2								0.95
NM_013174	transforming growth factor, beta 3 tgfb3; factor beta-3								0.95
mwgrat10K#8048	expression: brain kidney; strains: shrsp; similar to pir nf00134977								0.95
NM_021764	protein kinase c-binding protein beta15 loc60383; beta 15								0.95
NM_022921	rt1 class ib gene rt1-m3; m3 protein								0.95
mwgrat10K#7214	expression: brain heart; strains: shrsp; similar to pir nf00525571								0.95
mwgrat10K#7747	expression: liver kidney heart; strains: shrsp sprague_dawley								0.95
mwgrat10K#7393	expression: liver kidney; strains: wistar_kyoto; similar to pir								0.95

GeneID	GeneName	Signals	Brain	Heart	Kidney	Liver	Lung	Spleen	OSI
mwgrat10K#6358	expression: heart brain; strains: sprague_dawley wistar_kyoto;								0.95
L07409	immunoglobulin kappa-chain igkv								0.95
AF022774	rabphilin-3a related protein								0.95
AF280423	annexin vii								0.94
NM_019301	complement receptor related protein cr1; 5i2 antigen precursor; type 1;								0.94
NM_053812	bcl2-antagonist/killer 1 bak1; bak protein								0.94
AF323608	prothrombinase fgl2								0.94
AF130341	melatonin receptor mt1								0.94
X63594	rl/if-1								0.94
AF246120	hephaestin; heph								0.94
AF020618	progression elevated gene 3 protein								0.94
NM_012553	reading frame preproelastase ii; elastase precursor; 2, pancreatic								0.94
NM_019195	integrin-associated protein form 4 iap; cd47								0.94
NM_012591	interferon regulatory factor 1 irf-1; irf1								0.94
mwgrat10K#7859	expression: heart brain; strains: sprague_dawley; similar to								0.94
L08134	glycoprotein sgp158								0.94
NM_053502	atp-binding cassette, sub-family g white, member 1 abcg1; abc								0.94
D13555	t-cell receptor zeta chain								0.94
NM_017113	epithelin 1 _2; granulin; grn								0.93
X62528	ribonuclease inhibitor								0.93
mwgrat10K#6817	expression: liver; strains: shrsp; similar to pir nf00500913 lim								0.93
NM_023965	endothelial type gp91-phox gp91-phox; nadph oxidase beta								0.93
NM_019289	actin-related protein complex 1b arpc1b; p41-arc								0.93

GeneID	GeneName	Signals	Brain	Heart	Kidney	Liver	Lung	Spleen	OSI
AF295360	cd44 isoform v6	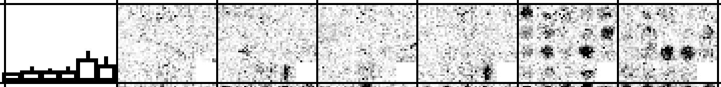							0.93
AF295301	snail	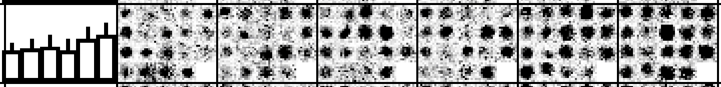							0.93
AF005929	cadherin-14	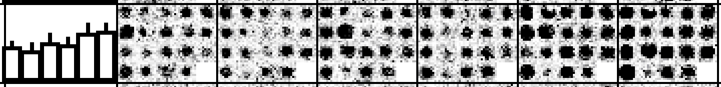							0.92
mwgrat10K# 8139	expression: liver brain; strains: wistar_kyoto; similar to	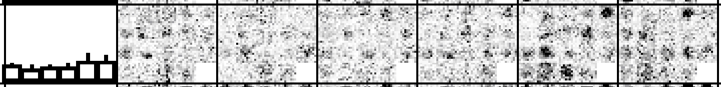							0.92
NM_053563	nuclear rna helicase, decd variant of dead box family ddxl; helicase	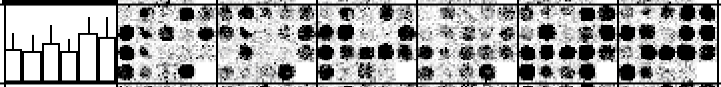							0.92
D10729	proteasome subunit rc1	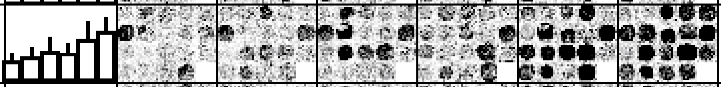							0.92
NM_031799	paired box gene 4 pax4; pax4a; pax4b	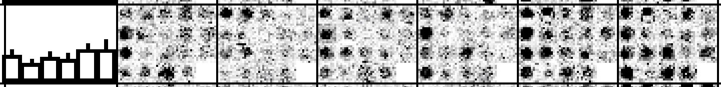							0.92
mwgrat10K# 6953	expression: brain; strains: sprague_dawley wistar_kyoto;	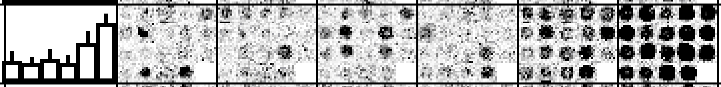							0.92
NM_012984	myosin heavy chain myr5; ixb	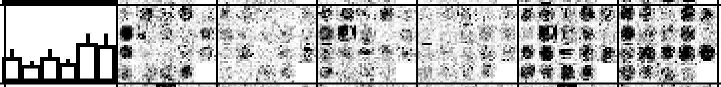							0.92
AF159356	munc13-4 protein munc13-4	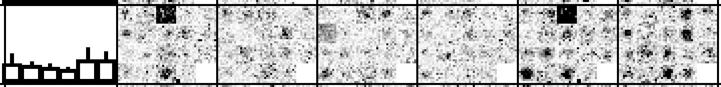							0.92
X16262	myosin heavy chain 21 aa 621	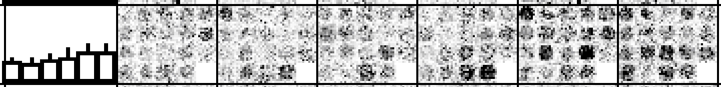							0.91
X63281	n-myc protein n-myc	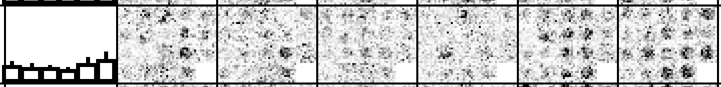							0.91
mwgrat10K# 7031	expression: brain kidney; strains: wistar_kyoto; similar to pir	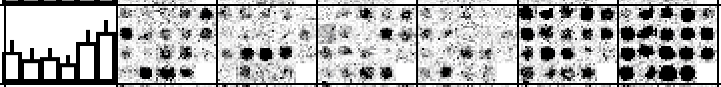							0.91
NM_022634	leucocyte specific transcript 1 lst1; b144	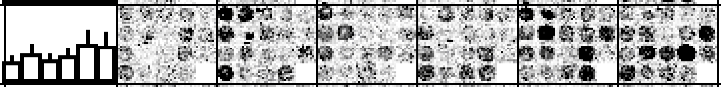							0.91
U60085	cytochrome p450olf3 cyp3a9; p450 3a9	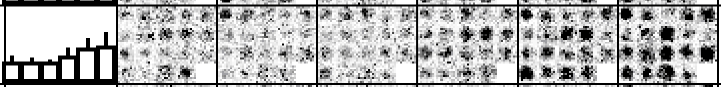							0.91
mwgrat10K# 8312	expression: kidney brain heart; strains: shrsp sprague_dawley	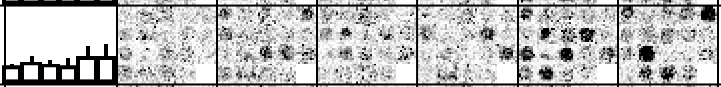							0.91
AF349115	cxc chemokine rtck1 rtck1	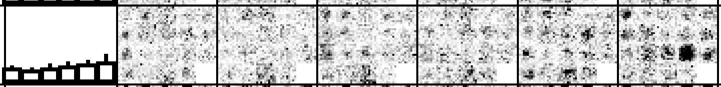							0.91
mwgrat10K# 8741	expression: kidney; strains: wistar_kyoto; similar to	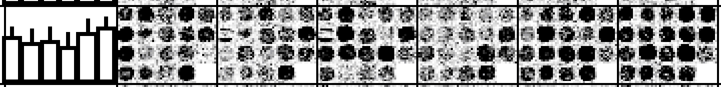							0.91
NM_017006	glucose-6-phosphate dehydrogenase g6pd; gd aa 1-515	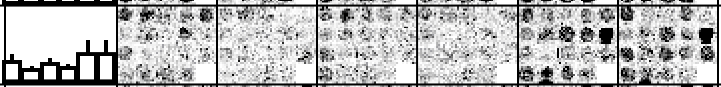							0.91
AF329827	zyxin	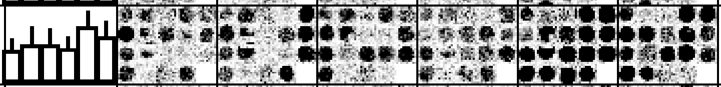							0.91
AF074609	mhc class i antigen rt1.ec3	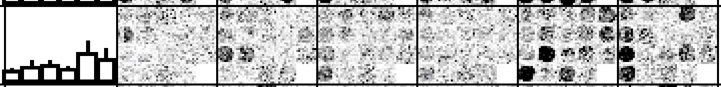							0.91
mwgrat10K# 7205	expression: brain; strains: shrsp wistar_kyoto; similar to	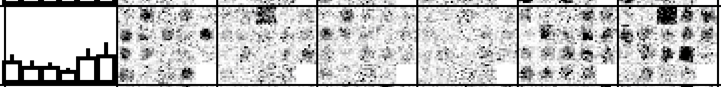							0.90
AF036335	nono/p54nrb homolog	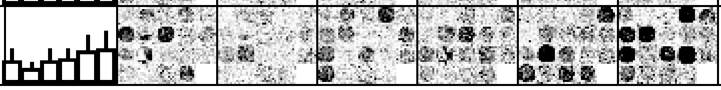							0.90

GeneID	GeneName	Signals	Brain	Heart	Kidney	Liver	Lung	Spleen	OSI
mwgrat10K# 7217	expression: brain heart; strains: shrsp; similar to								0.90
NM_057124	pyrimidinergic receptor p2y, g-protein coupled, 6 p2ry6; novel g								0.90
NM_053970	neurotensin-degrading neutral metalloendopeptidase; neurolysin								0.90
L20992	t cell receptor								0.90
NM_053340	opioid growth factor receptor ogfr								0.90
mwgrat10K# 7487	expression: liver brain; strains: shrsp sprague_dawley wistar_kyoto;								0.90
NM_012900	ameloblastin precursor; ambn; amelin 2								0.89
AF036537	homocysteine respondent protein hcyp2								0.89
NM_022955	megf6 egfl3								0.89
NM_030585	fos-related antigen fra								0.89
mwgrat10K# 9595	expression: kidney; strains: shrsp; similar to gbp|bc011981|bc011981_1								0.89
mwgrat10K# 7739	expression: kidney brain; strains: shrsp wistar_kyoto; similar to pir								0.89
mwgrat10K# 7279	expression: brain kidney; strains: sprague_dawley wistar_kyoto;								0.89
AJ132008	phocein protein phocein								0.89
AF385833	rac1								0.89
mwgrat10K# 7424	expression: liver brain; strains: shrsp wistar_kyoto; similar to								0.89
mwgrat10K# 7574	expression: brain kidney; strains: shrsp wistar_kyoto; similar to								0.89
L08814	ciidbp								0.89
mwgrat10K# 6998	expression: brain heart; strains: shrsp wistar_kyoto; similar to								0.89
J02712	protease ii prt								0.88
NM_021909	related to ion channel; fxyd5								0.88
NM_053926	phosphatidylinositol-4-phosphate 5-kinase, type ii, alpha pip5k2a;								0.88
mwgrat10K# 9717	expression: kidney; strains: shrsp; mwg own new gene sequence								0.88

GeneID	GeneName	Signals	Brain	Heart	Kidney	Liver	Lung	Spleen	OSI
AF062594	nucleosome assembly protein	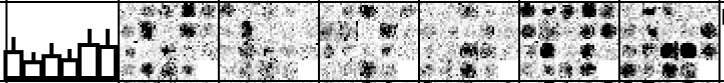							0.88
NM_013069	cd74 antigen invariant polpypeptide of major histocompatibility class ii	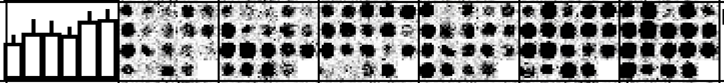							0.88
NM_012889	vascular cell adhesion molecule 1 vcam-1; molecule-1; vcam1	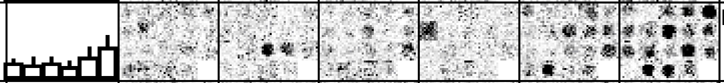							0.88
NM_017194	acetylcholine receptor epsilon subunit preprotein aa -20 to 473;	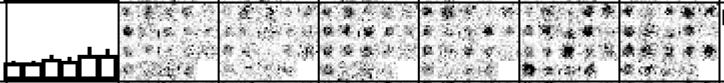							0.87
NM_022384	achaete-scute complex drosophila homolog-like 1 ascl1; put. mash-1	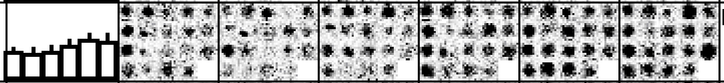							0.87
mwgrat10K# 8068	expression: liver heart; strains: shrsp; similar to	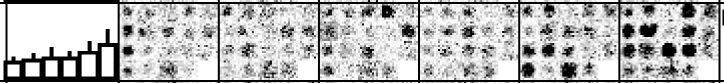							0.87
D88461	n-wasp	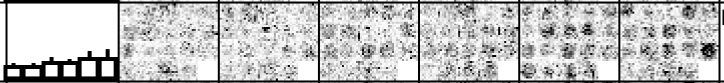							0.87
NM_022391	pituitary tumor transforming gene protein pttg; tumor-transforming 1	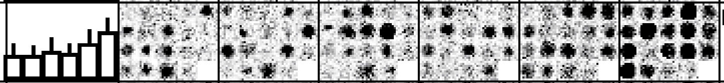							0.87
M55075	type iii adenylyl cyclase adenylyl cyclase	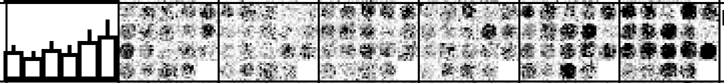							0.87
AF082535	type iii multi-pass transmembrane protein	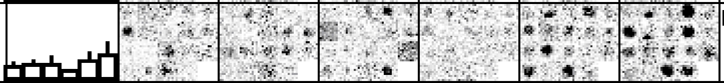							0.87
U31168	sh3 domain binding protein cr16	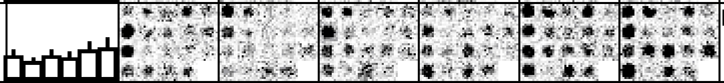							0.87
U56822	ly-49.12 antigen	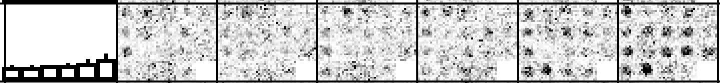							0.87
NM_031797	kangai 1 suppression of tumorigenicity 6, prostate kai1;	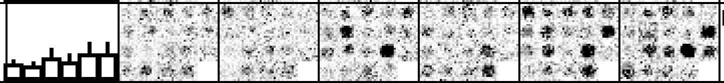							0.87
NM_021687 _1	receptor tyrosine kinase erbb4	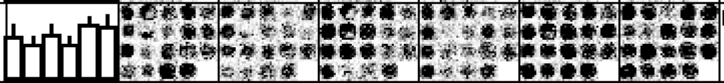							0.86
mwgrat10K# 7777	expression: brain; strains: shrsp sprague_dawley; similar to	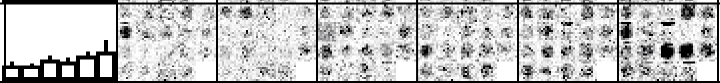							0.86
M25347	cyclic nucleotide phosphodiesterase	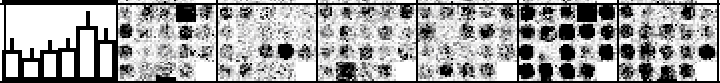							0.86
mwgrat10K# 8907	expression: liver kidney brain heart; strains: shrsp sprague_dawley	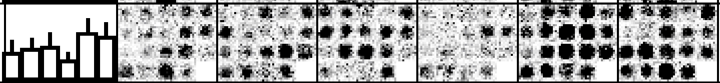							0.86
mwgrat10K# 8422	expression: liver; strains: shrsp; similar to gbp|bc014688|bc014688_1	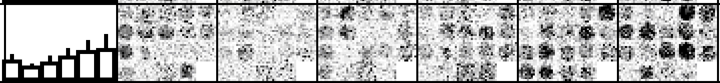							0.86
J05087_1	unknown protein	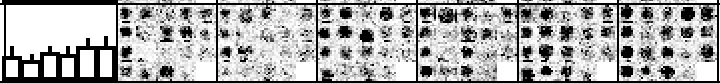							0.86
NM_031538	cd8 antigen, alpha-chain cd8a; ox-8 antigen	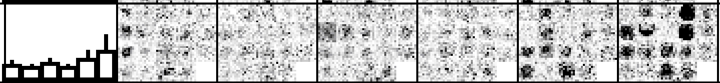							0.85
NM_023994	candidate taste receptor t2r3	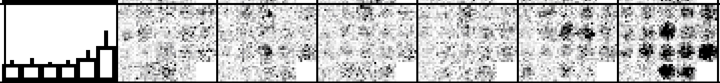							0.85
mwgrat10K# 7282	expression: liver brain kidney; strains: sprague_dawley	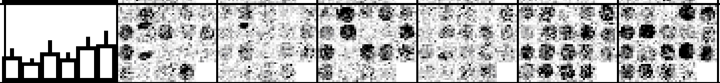							0.85
NM_013169	cd3 antigen delta polypeptide cd3d; precursor protein aa -21 to 152	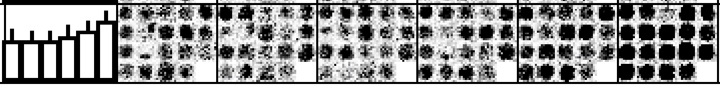							0.85

GeneID	GeneName	Signals	Brain	Heart	Kidney	Liver	Lung	Spleen	OSI
X53949	udp-galactose 4-epimerase aa 1-347								0.85
X62329	t-cell receptor v-alpha j-alpha rvalphaq4a13								0.85
AB008110_1	rt1-dob								0.84
U62315	alpha globin gloa								0.84
NM_022502	palmitoyl-protein thioesterase ppt								0.84
NM_013139	colipase pancreatic clps								0.84
NM_017207	vanilloid receptor-like protein 1 vrl-1; ion channel; stretch activated 2b								0.84
mwgrat10K#9019	expression: brain; strains: sprague_dawley; similar to								0.84
mwgrat10K#6911	expression: kidney brain; strains: sprague_dawley wistar_kyoto;								0.84
NM_023092	unconventional myosin myr2 i heavy chain myr2; myr 2								0.84
U10188	polo like kinase plk								0.84
NM_017248	heterogeneous nuclear ribonucleoprotein a1 hnrpa1; helix								0.84
NM_031539	cd8 antigen, beta-chain cd8b; 37k protein aa -21 to 187								0.83
NM_030989_1	tumor protein p53 li-fraumeni syndrome tp53; alternative splice								0.83
NM_057195	brain-enriched wd-repeat protein bwd; homolog of c. elegans smu-1								0.83
NM_053675	spermatogenesis associated 2; spata2								0.83
NM_022591	telomerase protein component 1 tep1; tlp1								0.83
NM_012873	protamine 2 prm2								0.83
D28557	ryb-a; yb2								0.83
NM_022207	transmembrane receptor unc5h2 unc5h2								0.83
NM_013036	somatostatin receptor subtype 4 major hippocampal somatostatin								0.83
U50355	neutrophil defensin 4 ratnp-4; ratnp-3 precursor; 3a ratnp-3a; 3b ratnp-3b								0.83
NM_053538	lysosomal-associated protein transmembrane 5 laptm5; gcd-10								0.82

GeneID	GeneName	Signals	Brain	Heart	Kidney	Liver	Lung	Spleen	OSI
NM_031035	guanine nucleotide-binding protein alpha subunit; gtp-binding; g-alpha-i2								0.82
mwgrat10K# 8973	expression: brain; strains: sprague_dawley; similar to pir								0.82
mwgrat10K# 7665	expression: kidney; strains: wistar_kyoto; similar to								0.82
AF084932	class ii mhc rt1.d a beta-chain precursor rt1.d a; n; u; e-beta								0.81
NM_012627	camp-dependent protein kinase catalytic subunit binding inhibtor 2								0.81
V01257	unidentified reading frame								0.81
AF003944	ovalbumin upstream promoter beta nuclear receptor rcoupb								0.81
NM_022283 _1	allograft inflammatory factor-1 splice variant g1; mrf-1; iba1, ionized								0.81
NM_053520 _1	e74-like factor 1 ets domain transcription factor elf1; elf-1;								0.81
X56596_1	mhc a-beta rt1.b-b-beta cell surface glycoprotein; integral membrane								0.80
NM_022715	major vault protein mvp								0.80
X15836	oncomodulin								0.80
mwgrat10K# 7638	expression: liver kidney brain; strains: shrsp sprague_dawley								0.80
NM_019297	cholinergic receptor, nicotinic, beta polypeptide 2 chrnb2; nicotinic								0.79
NM_024133	huntingtin-associated protein 1 hap1; rhap1-a								0.79
NM_017175	cardiolipin/protease-activated protein kinase-1 pak-1; pkn; kinase c-like 1								0.78
mwgrat10K# 8261	expression: brain heart kidney; strains: shrsp wistar_kyoto; mwg								0.78
S73971	muscarinic acetylcholine receptor m1								0.78
mwgrat10K# 7366	expression: liver brain; strains: sprague_dawley wistar_kyoto;								0.78
NM_031328	b-cell cll/lymphoma 10 bcl10; r-rcd1								0.78
X05472	orf1								0.78
mwgrat10K# 6913	expression: kidney brain; strains: shrsp wistar_kyoto; similar to pir								0.78
NM_017124	cd37 antigen cd37								0.77

GeneID	GeneName	Signals	Brain	Heart	Kidney	Liver	Lung	Spleen	OSI
NM_019165	interferon-gamma inducing factor precursor igif; interleukin-18 il-18;	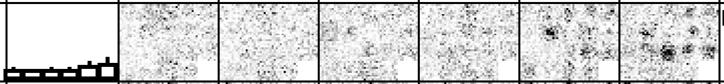							0.77
NM_017095	ccaat / enhancer binding protein c/ebp, epsilon cebpe;	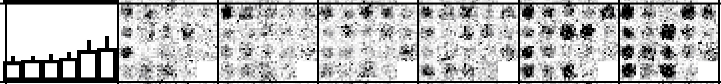							0.77
mwgrat10K#9076	expression: heart; strains: sprague_dawley; similar to pir	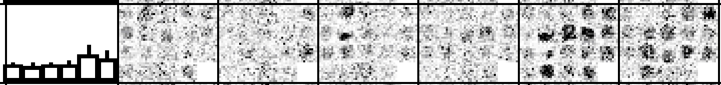							0.77
NM_012894	adenosine deaminase, rna-specific, b1 adarb1; double-stranded	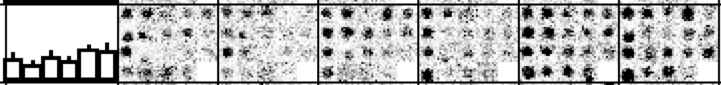							0.76
NM_022205	chemokine receptor cxcr4 cxcr4; cxc ccr4; lcr1	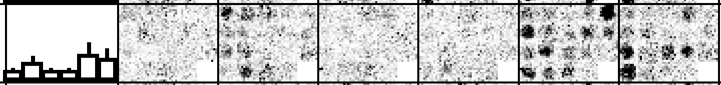							0.76
NM_020074	chondroitin sulfate proteoglycan core protein precursor; peptide; pgsg	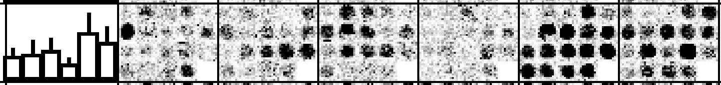							0.75
U95920	pcm-1	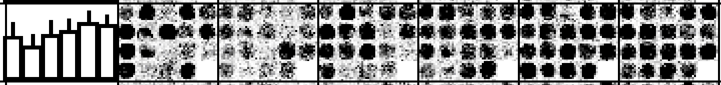							0.75
NM_031832	ige binding protein lgals3	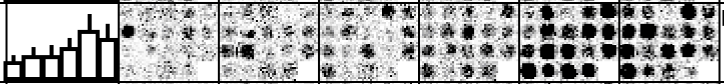							0.75
NM_019285	adenylyl cyclase type iv; 4 adcy4	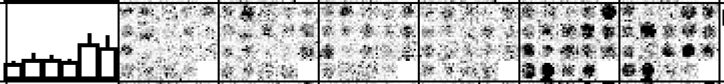							0.75
X14254	invariant chain aa 1-280	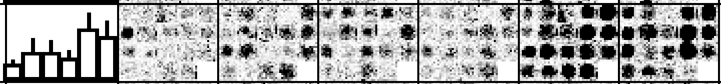							0.74
NM_019235	gamma-glutamyltransferase-like activity 1 ggtla1; gamma glutamyl	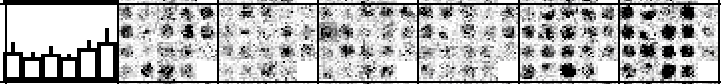							0.74
NM_012773	a-kinase anchoring protein akap; 220	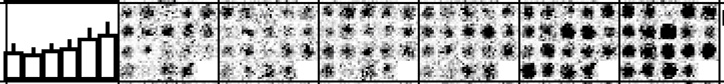							0.73
X58631	protein-tyrosine kinase csk	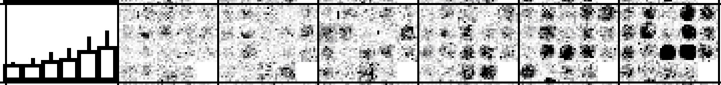							0.73
NM_022265	programmed cell death 4 pdcd4; death-upregulated gene dug	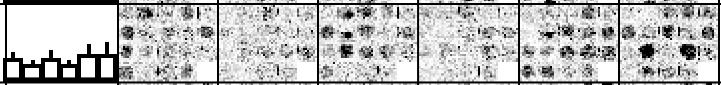							0.73
NM_054008	rgc32 protein rgc32; rgc-32	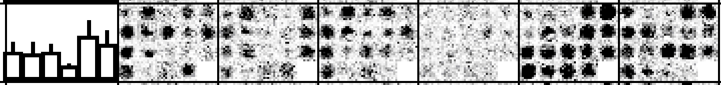							0.72
NM_053587	s100 calcium-binding protein a9 calgranulin b s100a9; intracellular	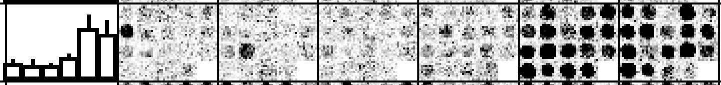							0.72
mwgrat10K#8856	expression: kidney; strains: wistar_kyoto; similar to	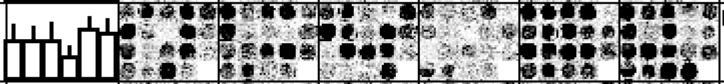							0.70
mwgrat10K#7194	expression: kidney brain; strains: shrsp wistar_kyoto; similar to pir	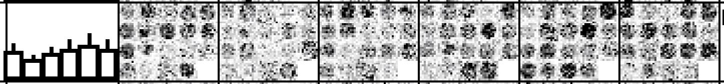							0.70
NM_053879	neural cell adhesion protein big-2 precursor big-2; axonal-associated	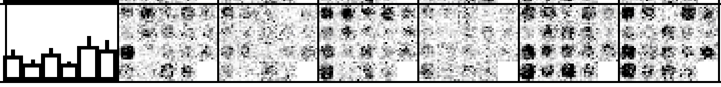							0.64
