# Supplementary material for: Identification of rat lung – prominent genes by a parallel DNA microarray hybridization
Source: BMC Genomics. 2006 Mar 13;7:47. doi: 10.1186/1471-2164-7-47 (PMC1523215; doi:10.1186/1471-2164-7-47)
Supplement: Additional File 4 — Supplementary Table E3, Main functional categories of one organ-prominent genes. [file 1471-2164-7-47-S4.doc]

**Table E3:**  Main functional categories of organ-prominent genes

| Meaning | Brain | Heart | Kidney | Liver | Lung | Spleen |
| --- | --- | --- | --- | --- | --- | --- |
| Function unclear | 98 | 67 | 107 | 169 | 55 | 35 |
| Molecular_function | 70 | 22 | 48 | 115 | 64 | 26 |
| Binding | 40 | 12 | 16 | 50 | 35 | 17 |
| Metal ion binding | 17 |  | 5 | 11 | 6 |  |
| Nucleic acid binding |  |  |  | 7 | 5 | 5 |
| Nucleotide binding |  |  |  | 9 | 8 | 7 |
| **Protein binding *** | **13** |  |  |  | **10** |  |
| **Receptor binding** |  |  |  |  | **5** |  |
| Catalytic activity | 19 | 7 | 24 | 63 | 18 | 14 |
| Hydrolase activity | 8 |  | 11 | 22 | 8 | 7 |
| **Oxidoreductase activity** |  |  | **6** | **18** |  |  |
| Transferase activity | 5 |  |  | 17 | 6 | 5 |
| **Enzyme regulator activity** |  |  |  | **7** |  |  |
| Signal transducer activity | 13 | 7 | 7 | 22 | 15 | 9 |
| Receptor activity | 10 |  |  | 10 | 9 | 5 |
| Transporter activity | 8 |  | 14 | 21 | 9 |  |
| Biological_process | 64 | 22 | 38 | 111 | 67 | 25 |
| Cellular process | 40 | 12 | 18 | 39 | 38 | 13 |
| Cell communication | 20 | 6 |  | 18 | 22 | 7 |
| Signal transduction | 13 | 5 |  | 10 | 15 | 6 |
| Cellular physiological process | 19 | 8 | 16 | 24 | 18 | 6 |
| Cell growth and/or maintenance | 17 | 5 | 14 | 23 | 15 | 6 |
| Development | 14 |  |  | 6 | 14 |  |
| **Morphogenesis** | **14** |  |  |  | **12** |  |
| Physiological process | 30 | 15 | 31 | 92 | 50 | 20 |
| Cellular physiological process | 12 |  | 12 | 18 | 17 | 5 |
| Cell growth and/or maintenance | 11 |  | 11 | 16 | 14 |  |
| **Coagulation** |  |  |  | **7** |  |  |
| Metabolism | 13 | 8 | 20 | 55 | 26 | 15 |
| Organismal physiological process | 10 | 5 |  | 16 | 9 |  |
| **Response to stimulus** |  |  |  | **17** | **11** |  |

* Bold font indicts that the respective functional categories existed in two or less groups of organ-prominent genes. An indented functional category is a child of a topper one with less proceeding space.
