# Supplementary material for: Identification of rat lung – prominent genes by a parallel DNA microarray hybridization
Source: BMC Genomics. 2006 Mar 13;7:47. doi: 10.1186/1471-2164-7-47 (PMC1523215; doi:10.1186/1471-2164-7-47)
Supplement: Additional File 5 — Supplementary Table E4, Main functional categories of two organ-prominent genes. [file 1471-2164-7-47-S5.doc]

**Table E4:** Main functional categories of co-expressed genes between two organs

| Functional category | Lung- heart | lung- kidney | lung- liver | lung-  spleen | lung- brain | brain- kidney |
| --- | --- | --- | --- | --- | --- | --- |
| Function unclear | 7 | 34 | 14 | 67 | 6 | 86 |
| Molecular_function | 7 | 25 | 8 | 45 | 4 | 59 |
| Binding | 3 | 14 | 3 | 29 | 3 | 22 |
| **Nucleic acid binding *** |  |  |  | **8** |  |  |
| **Nucleotide binding** |  |  |  | **5** |  |  |
| Protein binding |  | 3 |  | 6 |  | 6 |
| Catalytic activity |  | 12 | 5 | 11 |  | 40 |
| Hydrolase activity |  | 7 | 3 | 6 |  | 9 |
| **Oxidoreductase activity** |  |  |  |  |  | **17** |
| **Transferase activity** |  |  |  |  |  | **10** |
| **Signal transducer activity** |  |  |  | **12** |  |  |
| **Transporter activity** |  |  |  | **6** |  | **9** |
| Biological_process | 8 | 25 | 4 | 49 | 3 | 44 |
| Cellular process |  | 10 |  | 25 | 3 | 15 |
| **Cell communication** |  | **5** |  | **17** |  |  |
| **Signal transduction** |  | **5** |  | **12** |  |  |
| Cellular physiological process |  | 4 |  | 9 |  | 13 |
| **Cell growth and/or maintenance** |  |  |  | **7** |  | **13** |
| Development | 4 | 4 |  | 9 |  |  |
| Morphogenesis | 3 | 3 |  | 5 |  |  |
| Physiological process | 4 | 21 | 3 | 34 |  | 34 |
| **Cellular physiological process** |  | **4** |  | **10** |  |  |
| **Cell growth and/or maintenance** |  | **4** |  | **8** |  |  |
| Metabolism | 3 | 13 |  | 16 |  | 30 |
| **Organismal physiological process** |  |  |  | **10** |  |  |
| **Immune response** |  |  |  | **6** |  |  |
| **Response to stimulus** |  |  |  | **10** |  |  |

* Bold font indicts that the respective functional categories existed in two or less groups of co-expression genes. An indented functional category is a child of a topper one with less proceeding space.
