# Supplementary material for: Identification of rat lung – prominent genes by a parallel DNA microarray hybridization
Source: BMC Genomics. 2006 Mar 13;7:47. doi: 10.1186/1471-2164-7-47 (PMC1523215; doi:10.1186/1471-2164-7-47)
Supplement: Additional File 6 — Table 2: DNA microarray signal intensities and spot images of 13 verified genes [file 1471-2164-7-47-S6.pdf]

Table 2: DNA microarray signal intensities and spot images of 13 verified genes*

GeneID	GeneName	Signals	Brain	Heart	Kidney	Liver	Lung	Spleen	OSI
NM_012967	Intercellular adhesion molecule 1 (ICAM-1)	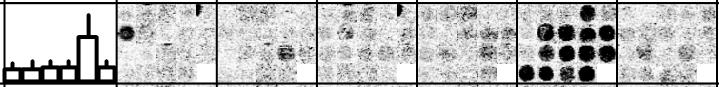							1.00
NM_019231	Mitogen activated protein kinase 13 (Mapk13)	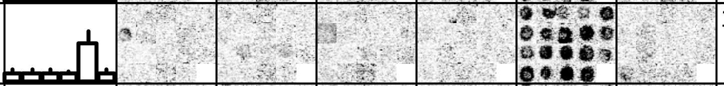							1.00
NM_031764	Discoidin domin receptor family, member 2 (Ddr2)	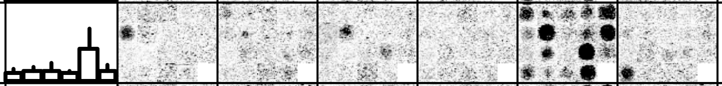							1.00
NM_012521	Vitamin D-dependent calcium-binding protein (Calb3)	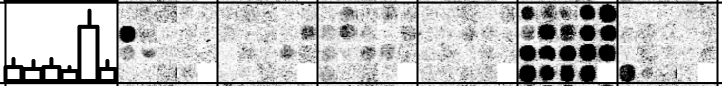							1.00
AF068861	Beta defensin-2 (BD-2)	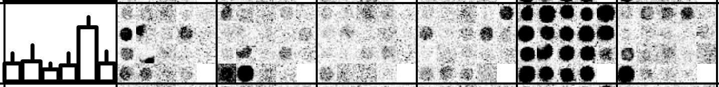							0.99
NM_031684	Solute carrier family 29 transporters, member 1 (Slc29a1)	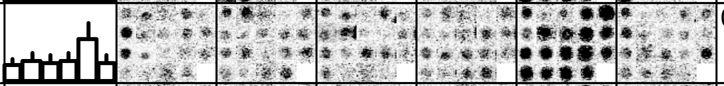							0.99
X13295a	Lipocalin 2 (alpha-2u protein, Lcn2)	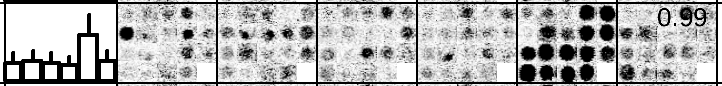						0.99	
NM_053380	Solute carrier family 34 sodium phosphate, member 2 (Slc34a2)	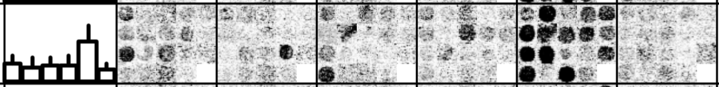							0.99
AF346790	Melanoma-associated antigen (Mg50)	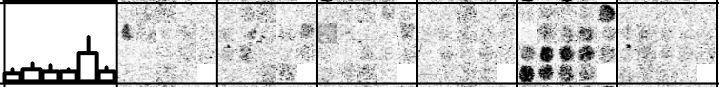							0.99
AF089866	Keratin 19 (K19)	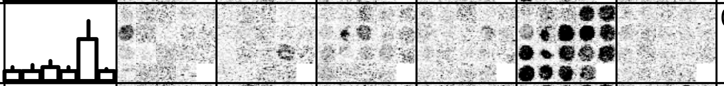							0.99
NM_012878	Pulmonary surfactant protein D (SP-D)	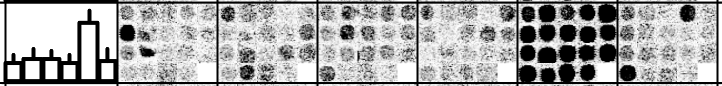							0.99
NM_030999	Corticotropin releasing hormone receptor (Crhr1)	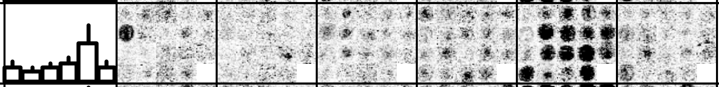							0.98
NM_053336	Advanced glycosylation end product-specific receptor (Ager)	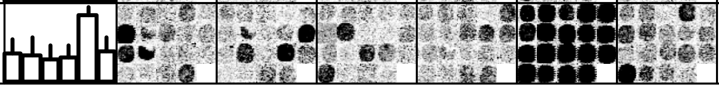							0.98

* Gene ID: Genbank accession number; GeneName: name and common symbol of a gene; signals: mean scaled fluorescence intensity of brain, heart, kidney, lung, and spleen; the error bars were based on standard deviation. OSI: Organ specific index (see text for detail)
